# Taxonomic Advances from Fungal Flora Associated with Ferns and Fern-like Hosts in Northern Thailand

**DOI:** 10.3390/plants12030683

**Published:** 2023-02-03

**Authors:** Elaheh Seifollahi, Antonio Roberto Gomes de Farias, Ruvishika Shehali Jayawardena, Kevin D. Hyde

**Affiliations:** 1 Center of Excellence in Fungal Research, Mae Fah Luang University, Chiang Rai 57100, Thailand; 2 School of Science, Mae Fah Luang University, Chiang Rai 57100, Thailand

**Keywords:** *Colletotrichum*, *Curvularia*, *Diaporthe*, *Fusarium*, *Lasiodiplodia*, pestalotiod fungi

## Abstract

Ferns are one of the most significant plant groupings that comprise a substantial proportion of the plant flora due to the fact of their great diversity, especially in tropical areas. The biodiversity of fungi associated with ferns and fern-like hosts has also received little attention in studies. Plant samples were collected from diseased and dead plants of ten fern or fern-like species from Chiang Rai in northern Thailand. Forty-one isolates were selected from the obtained isolates for molecular and morphological analysis, with a focus on pathogenic fungal genera and consideration of the diversity in host and geographical location. Twenty-six species belonging to seven genera (*Colletotrichum*, *Curvularia*, *Diaporthe*, *Fusarium*, *Lasiodiplodia*, *Neopestalotiopsis*, and *Pestalotiopsis*) in six families were identified. Thirty new hosts, eight new geographical hosts, and one new species, *Colletotrichum polypodialium*, are described. *Nepestalotiopsis phangngaensis*, *N. pandancola*, *Diaporthe tectonendophytica*, *D. chiangraiensis*, and *D. delonicis* were isolated for the first time from leaf spots. Additionally, new reservoirs and geographical locations for species previously isolated from leaf spots or whose pathogenicity was established were found. However, more studies are necessary to prove the pathogenicity of the fungi isolated from the leaf spots and to identify the fungi associated with other species of ferns.

## 1. Introduction

Ferns are considered an important component of plant diversity in Thailand, which consists of approximately 5–7% of the total flora in this country, and 670 taxa have been estimated [1]. Ferns also comprise a large proportion of the world’s plant flora (9000–12,000 species) [2,3]. They are used as ethnomedicine, pests controllers, food, and ornamental plants [4]. Additionally, they play a crucial role in ecology and interact with a variety of organisms, including insects and saprobic, endophytic, pathogenic, and mycorrhizal fungi [5].

Endophytes related to ferns have not been extensively studied. An investigation of the endophytes of seven fern species in Costa Rica revealed that more than 95% of the fungi belonging to *Ascomycota*, with *Dothideomycetes*, *Eurotiomycetes*, and *Sordariomycetes* dominating [6], with a prevalence of *Xylariales* [6]. Pathogenic *Ascomycota* and *Basidiomycota* have been isolated from ferns worldwide [7]. *Inocyclus angularis* was reported to cause tar spots on *Pleopeltis astrolepis* in Brazil [8] and *Milesina dryopteridis* to cause rust on *Rumohra adiantiformis* and *Pteris fauriei* in Japan [9]. *Pestalotiopsis maculans* causes leaf spots on *Lygodium venustum* in Argentina [10] and *Colletotrichum gloeosporioides* on *Lygodium microphyllum* and *L. japonicum* in the USA [11]. Additionally, *C. acutatum* causes anthracnosis in leather ferns in the USA [12] and *Fusarium thapsinum* brown rot in *Azolla microphylla* in India [13].

In contrast to endophytes and pathogens, saprobes have been frequently identified in ferns [5], accounting for 21 ascomycetes on the fronds and rachises of various ferns in Mexico [14]. More recently, new species have been introduced from this plant group: *Venustosynnema reniformisporum* from dead leaves of *Selaginella moellendorffii* [15], *Pestalotiopsis magna* from dead leaves of *Pteridium* sp. [16], and *Monilochaetes pteridophytophila* from the dead stalk of *Alsophila costularis* [17].

Given that Thailand is a center of fern diversity and few studies have investigated their diversity, it is assumed that this plant category is colonized by a wide range of fungal species. The current study aimed to identify saprobes and leaf spot fungi associated with ferns and fern-like species using molecular and morphological methods.

## 2. Results

This study identified 26 fungal species from fern and fern-like hosts in Chiang Rai, northern Thailand. The phylogenetic results and species descriptions for all species are provided below. 

***Dothideomycetes*** O.E. Erikss. & Winka;

***Botryosphaeriales*** C.L. Schoch, Crous & Shoemaker, Mycologia 98 (6): 1050 (2007).

This order includes six families: *Botryosphaeriaceae*, *Melanopsaceae*, *Phyllostictaceae*, *Planistromellaceae*, and *Saccharataceae* [18]. 

***Botryosphaeriaceae*** Theiss. & Syd (as “*Botryosphaeriacae*”), Annls mycol. 16(1/2): 16 (1918).

Members of this family are pathogens, endophytes, or saprobes on a wide range of hosts. They can also function as opportunists and primary plant pathogens [19,20]. Twenty-two genera are accepted in this family [18].

***Lasiodiplodia*** Ellis & Everh., *Botanical Gazette Crawfordsville*, 21: 92 (1896).

Based on molecular data, 35 species have been identified for this genus [18]. In the present study, two isolates obtained from ferns were identified as *L. thailandica* based on morphological and phylogenetic analyses of ITS, *tef1*, and *tub2* loci. 

**Figure 1:**
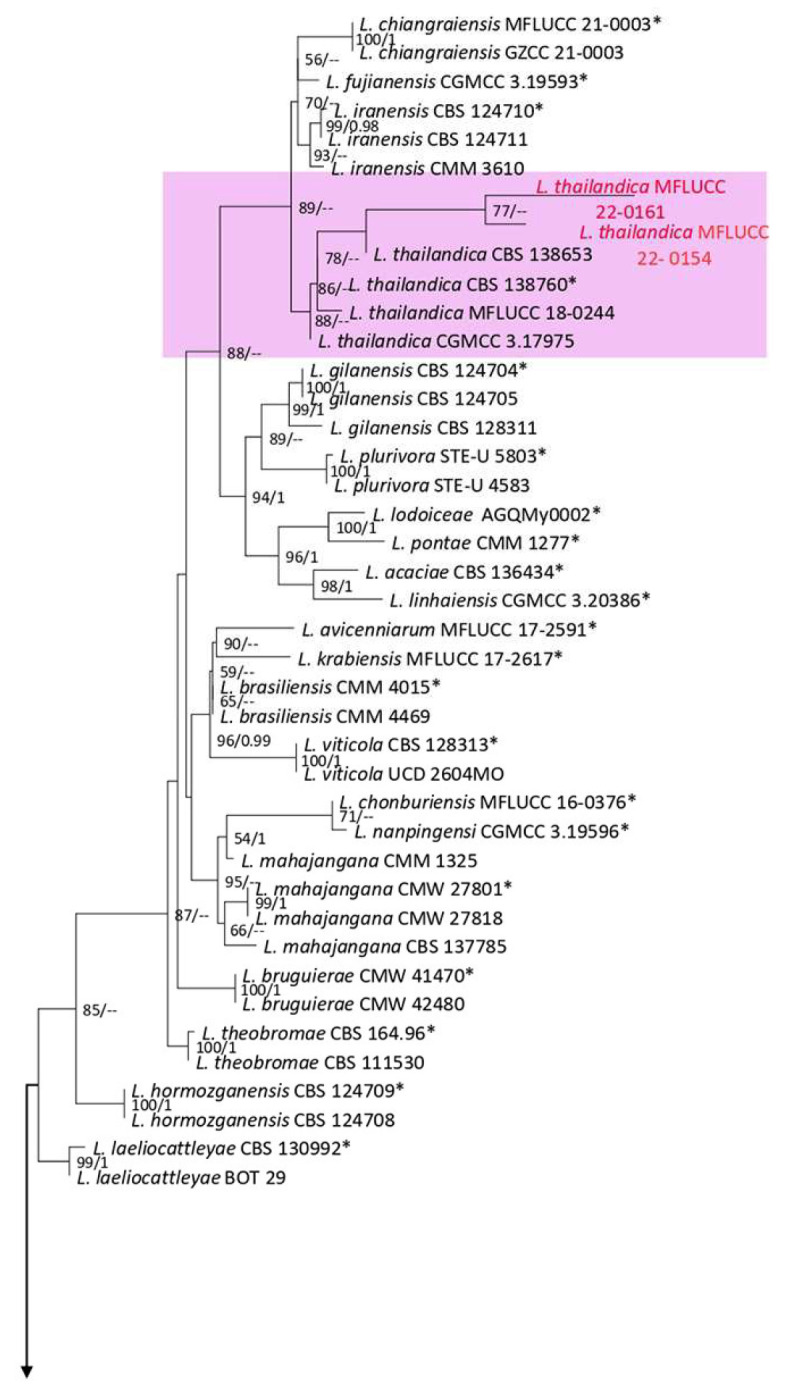

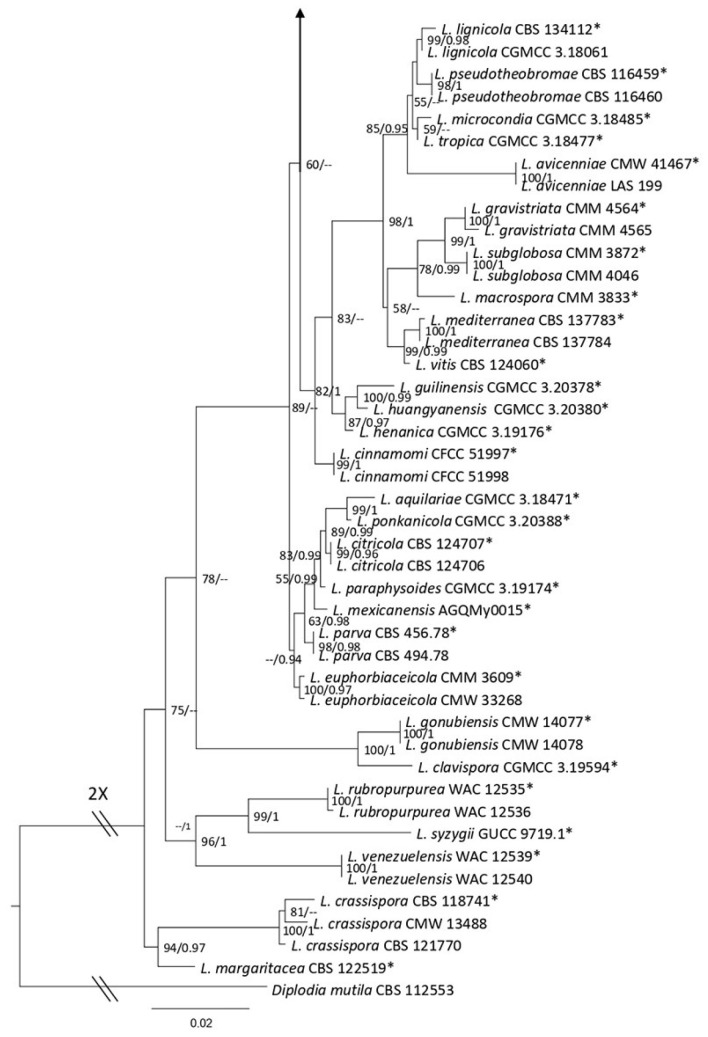
Maximum likelihood (ML) phylogenetic tree of *Lasiodiplodia* spp. obtained from the combined ITS, *tef1*, and *tub2* sequence data. The ultrafast maximum likelihood bootstrap support (%) ≥ 50 and BYPP ≥ 0.95 are shown at the nodes. The ex-type strains are marked with an asterisk. The tree is rooted in *Diplodia mutila* (CBS 112553).

***Lasiodiplodia thailandica*** Trakun., L. Lombard & Crous, 2014.

**Figure 2:**
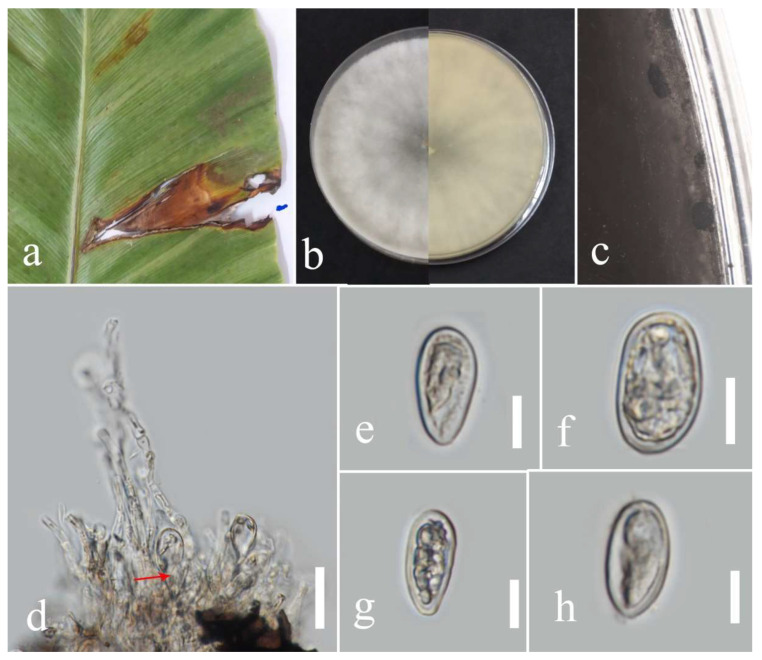
Morphology of *Lasiodiplodia thailandica* (MFLUCC 22-0161): (**a**) blight spots on *Asplenium nidus;* (**b**) upper reverse view of the colony after seven days of growth on PDA at 28 °C; (**c**) conidiomata; (**d**) conidiogenous cells; (**e**–**h**) conidia. Scale bars (**d**) = 20 µm and (**e**–**h**) = 10 µm.

Facesoffungi number: FoF 09333; Figure 2.

Associated with *Asplenium nidus* leaf spot. Sexual morph: not observed. Asexual morph: Conidiomata pycnidium, a few, scattered, singular, unilocular, superficial, and semi-immersed, globes, black with dark-brown outer layer and hyaline inner layer with hyphae tips around the outer wall of the pycnidium, brown, septate and rounded tips. Conidiophores reduced to conidiogenous cells. Conidiogenous cells cylindrical, hyaline, holoblastic, and thin walled. Paraphyses originated from the inner cells of pycnidium, cylindrical, hyaline, 1–3 septate, the basal cell swollen, thin walled. Conidia elliptic with granular content, hyaline at the immature stage, pale brown at maturity, aseptate, thick walled (1.1–1.7 µm), 15–26 × 9–16 µm (mean = 21.0 × 12.9, n = 30). 

Culture characteristics: Colonies filled a 90 mm Petri dish at seven days on PDA at 28 °C; fluffy, circular, and dull surface; medium density, and without pigmentation in the medium and fruiting body. The upper view was white, and the reverse primrose. 

Material examined: Thailand, Chiang Rai Province, Muang District, Huai Sak, on leaf spots of *Asplenium nidus* (*Aspleniaceae*); 17 December 2021, Elaheh Seifollahi, dried culture (MFLU 22-0207) and living culture MFLUCC 22-0161; Thailand, Chiang Rai Province, Muang District, Thasud, on dead leaves of *Nephrolepis cordifolia* (*Nephrolepidaceae*); 3 December 2021, Elaheh Seifollahi, dried culture (MFLU 22-0208) and living culture MFLUCC 22-0154. 

Notes: Isolated from blight spots on *Asplenium nidus*. The isolates obtained in this study (MFLUCC 22-0161 and MFLUCC 22-0154) were clustered with *L. thailandica* with 78% ML bootstrap support (Figure 1). A comparison of the ex-type strain *L. thailandica* (CBS 138760) with strains MFLUCC 22-0161 and MFLUCC 22-0154 revealed identical sequences in ITS (for both isolates) and *tef1* (only for MFLUCC 22-0154). The sequences of *tub2* for the type strain and *tef1* for strain MFLUCC 22-0161 are not available. This species was first reported from *Mangifera indica* and *Phyllanthus acidus* in Thailand [21], and here it was isolated from *Asplenium nidus* and *Nephrolepis cordifolia*. Hence, we provide new host records for this species. 

***Pleosporales*** Luttr. ex M.E. Barr, Prodromus to class *Loculoascomycetes*: 67 (1987).

*Pleosporales* is the largest order of *Dothideomycetes*, which covers a quarter of the fungi in this class and, currently, it comprises 92 families (OUTLINE OF FUNGI 2023). According to these authors, *Pleosporales* species are reported as pathogens, saprobes, endophytes, epiphytes, pathogen-like organisms on fungi or insects, and lichenized fungi. 

***Pleosporaceae*** Nitschke, Verh. naturh. Ver. preuss. Rheinl. 26: 74 (1869).

Based on molecular and morphological data, 23 genera are accepted in *Pleosporaceae*, including human pathogens and plant saprobes [22]. 

***Curvularia*** Boedijn, *Bull. Jard. bot*. Buitenz, 3 Sér. 13(1): 123 (1933).

*Curvularia* includes more than 170 epithets (Index Fungorum 2022). In this study, one isolate obtained from ferns was identified as *C. lunata* based on phylogenetic analyses of the ITS, *gapdh*, and *tef1* sequences and morphological data. 

**Figure 3:**
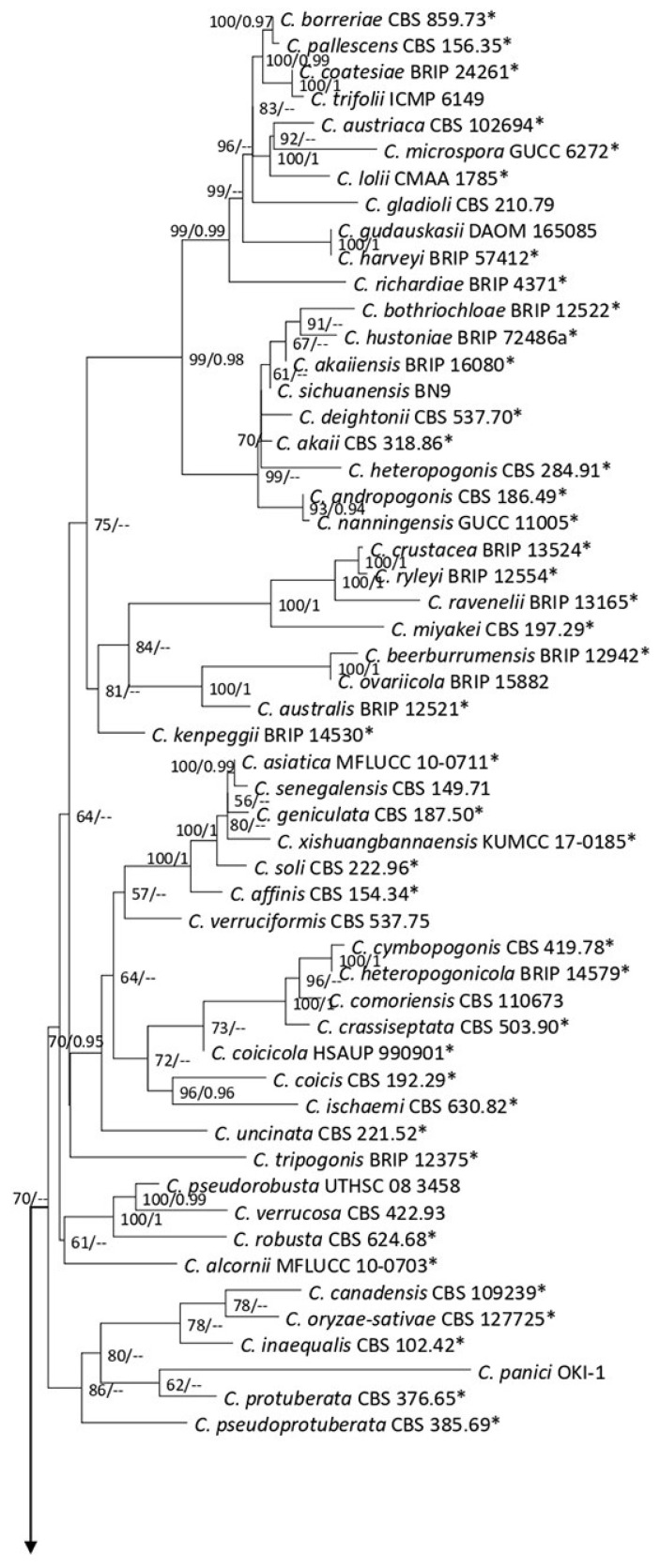

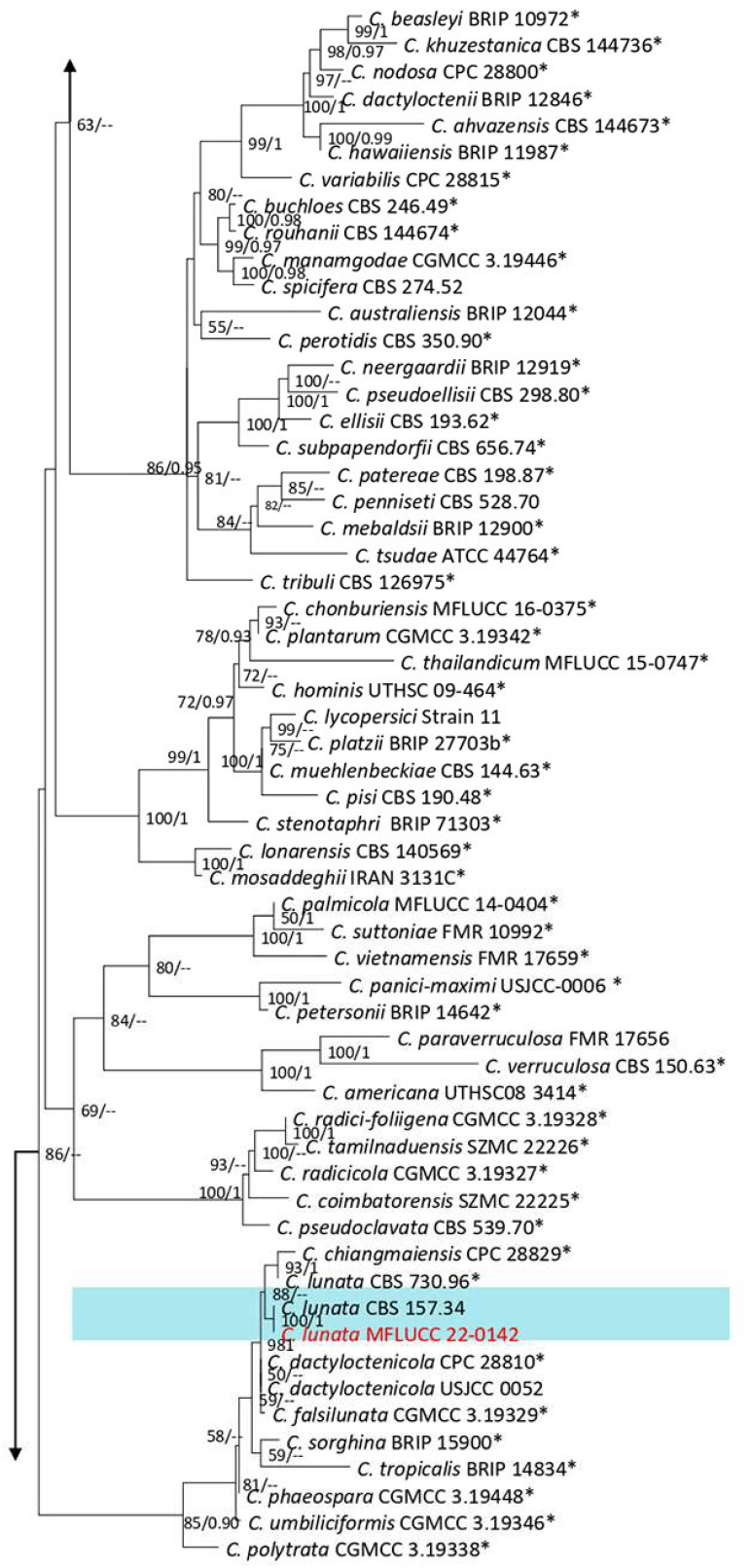

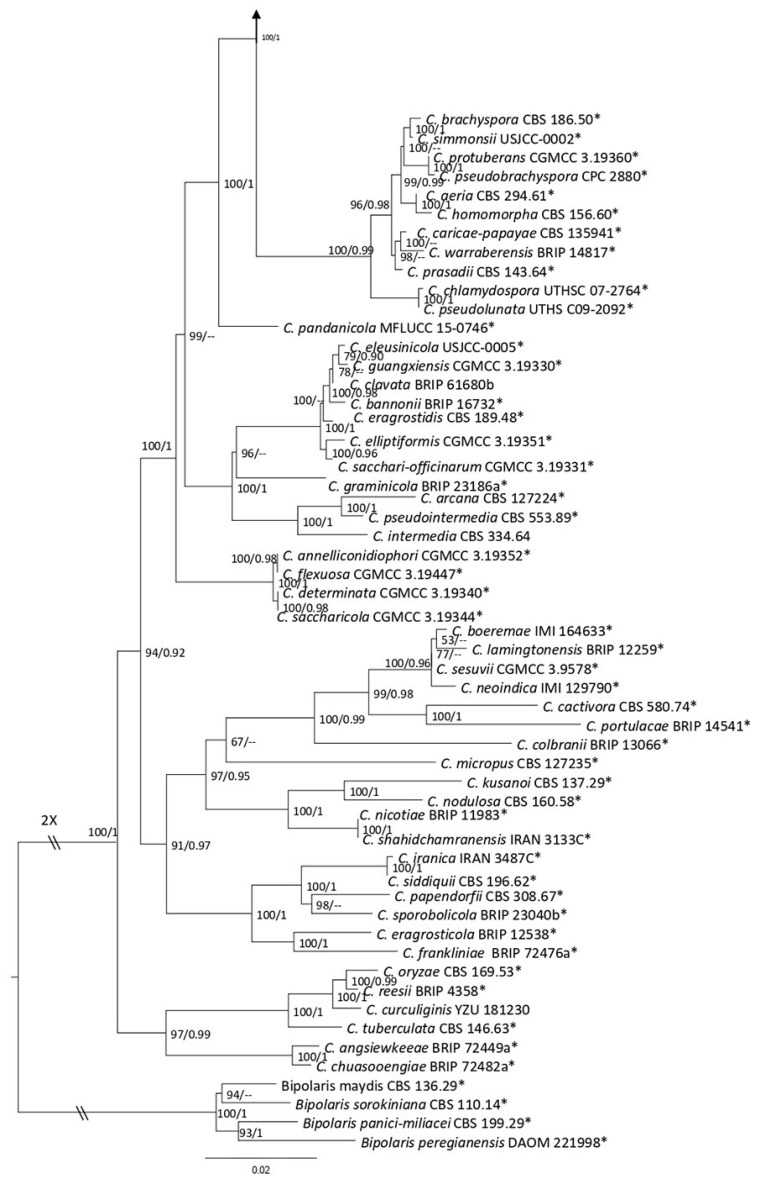
Maximum likelihood (ML) phylogenetic tree of *Curvularia* spp. Obtained from combined ITS, *gapdh*, and *tef1* sequence data. The ultrafast maximum likelihood bootstrap support (%) ≥ 50 and BYPP ≥ 0.90 are shown at the nodes. The ex-type strains are marked with an asterisk. The tree is rooted in *Bipolaris panici-miliacei* (CBS 199.29), *B. peregianensis* (DAOM 221998), *B. sorokiniana* (CBS 110.14), and *B. maydis* (CBS 136.29).

**Figure 4:**
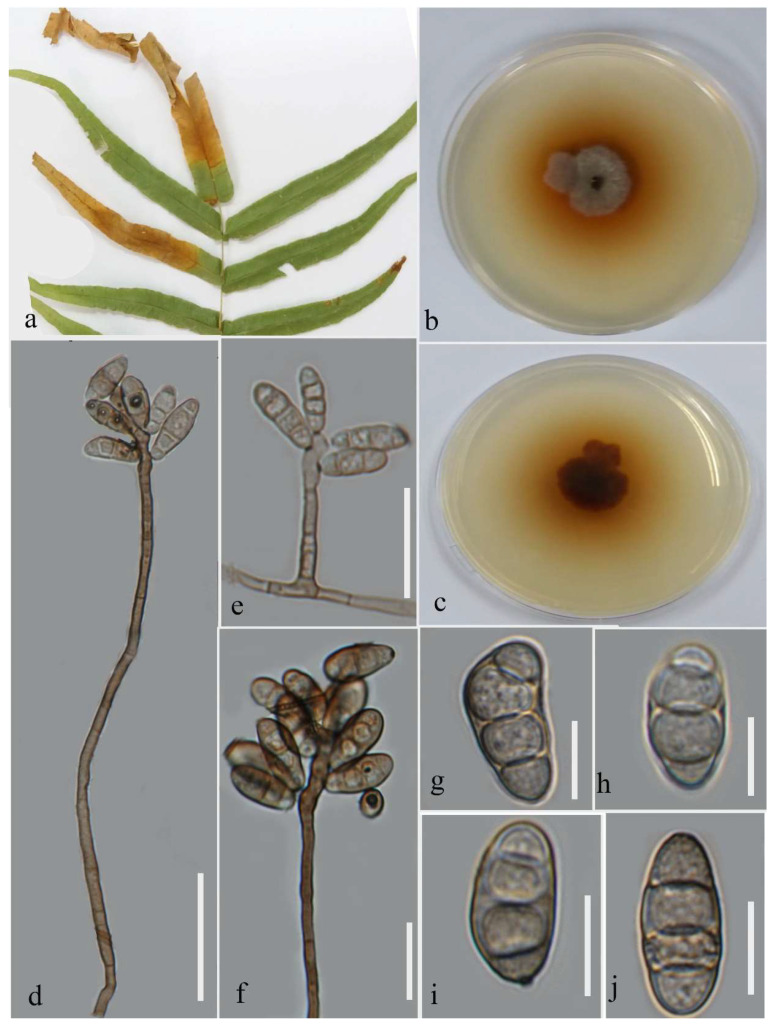
Morphology of *Curvularia lunata* (MFLUCC 22-0142): (**a**) blight on *Pteris grandifolia*; (**b**,**c**) upper and reverse views of the culture after seven days on PDA at 28 °C; (**d**–**f**) conidiophores and sympodial conidiogenous cells; (**g**–**j**) conidia. Scale bars (**d**) = 50 µm, (**e**–**f**) = 25 µm, and (**g**–**j**) = 10 µm.

***Curvularia lunata*** (Wakker) Boedijn, 1933; Figure 4.

Associated with *Pteris grandifolia* leaf blight. Sexual morph: not observed. Asexual morph: hyphae pale to medium brown; septate, 1.9–3.9 µm wide. Conidiophores straight to flexible, medium brown, septate, unbranched, geniculate toward the tip, 27–270 µm long. Conidiogenous cell sympodial, brown, terminal or intercalary, polytretic with dark scars. Conidia cylindrical to elliptic, pale to medium brown, 3-distoseptate, rounded at tips, the third cell from base swollen on one side in some spores, straight on one side, and convex on the opposite side, swollen cell larger with a similar color to other cells, 14–23 µm (mean = 17.96, n = 30) × 5–11 µm (mean = 7.8, n = 30). 

Culture characteristics: Colonies reached 17–27 mm after seven days of growth on PDA at 28 °C; felted, irregular shape, dull surface, lobate edge, well-defined margin, and medium density with pigmentation in media. Upper view smoke gray and reverse rust to chestnut. 

Material examined: Thailand, Chiang Rai Province, Muang District, Thasud, leaf spot on *Pteris grandifolia* (*Pteridaceae*), 4 December 2021, Elaheh Seifollahi, dried culture (MFLU 22-0209) and living culture MFLUCC 22-0142.

Notes: Isolated from blight spot on *Pteris grandifolia* leaves. The isolate obtained (MFLUCC 22-0142) clustered with *C. lunata*, with 100% ML bootstrap support and 1.0 BYPP (Figure 3). A pairwise comparison of the neotype strain (CBS 730.96) and the strain MFLUCC 22-0142 revealed a 0.82% nucleotide differences in *gapdh* (4 nucleotides) and 0.11% in *tef1*, while the ITS sequences were identical. This species was first reported from a lung biopsy in the USA and redescribed using a neotype [23]. It was found on *Brassica rapa*, *Morus* sp., *Oryza sativa*, *Panicum* sp., and *Zea mays* in Thailand [23,24,25,26]. Here, we provide a new host record for *C. lunata*. In the phylogenetic tree, *C. lunata* and *C. chiangmaiensis* clustered in the same clade with 90% ML bootstrap support, suggesting they may belong to the same species. Pairwise comparison of the DNA sequence data of the type strain of *C. lunata* (CBS 730.96) and *C. chiangmaiensis* (CPC 28829) revealed only 0.27% nucleotide differences in ITS (one nucleotide) and 0.34% in *tef1* (three nucleotides), while the sequences of *gapdh* were identical.

***Sordariomycetes*** O.E. Erikss. & Winka.

***Amphisphaeriales*** D. Hawksw. & O.E. Erikss., *Systema Ascomycetum* 5: 177 (1986).

This order comprises 88 genera and 17 families: *Amphisphaeriaceae*, *Apiosporaceae*, *Beltraniaceae*, *Castanediellaceae*, *Clypeophysalosporaceae*, *Cylindriaceae*, *Hyponectriaceae*, *Iodosphaeriaceae*, *Melogrammataceae*, *Oxydothidaceae*, *Phlogicylindriaceae*, *Pseudomassariaceae*, *Pseudosporidesmiaceae*, *Pseudotruncatellaceae*, *Sporocadaceae*, *Vialaeaceae*, and *Xyladictyochaetaceae*. The taxonomic treatment of this order is based on Hyde et al. [27].

***Sporocadaceae*** Corda, Icones fungorum hucusque cognitorum 5: 34 (1842).

This family included 23 genera, including endophytes, saprobes, plant pathogens, and parasites of animals and humans. Members of *Sporocadaceae* are characterized by acervuli, septate, hyaline, and pale to dark brown conidia. Additionally, they are distinguished by the sequence data of ITS, LSU, and *rpb2* [27]. 

***Neopestalotiopsis*** Maharachch., K.D. Hyde & Crous, *Studies in Mycology* 79: 135.

*Neopestalotiopsis* species are found as saprobes or plant pathogens. Due to the versicolorous median cells of the conidia, this genus was recognized from *Pesatalotiopsis* [28]. This study reports seven *Neopestalotiopsis* species associated with ferns based on morphomolecular justification: *N. guajavicola*, *N. hydeana*, *N. musae*, *N. pandanicola*, *N. phangngaensis*, *N. psidii*, and *N. saprophytica*.

**Figure 5:**
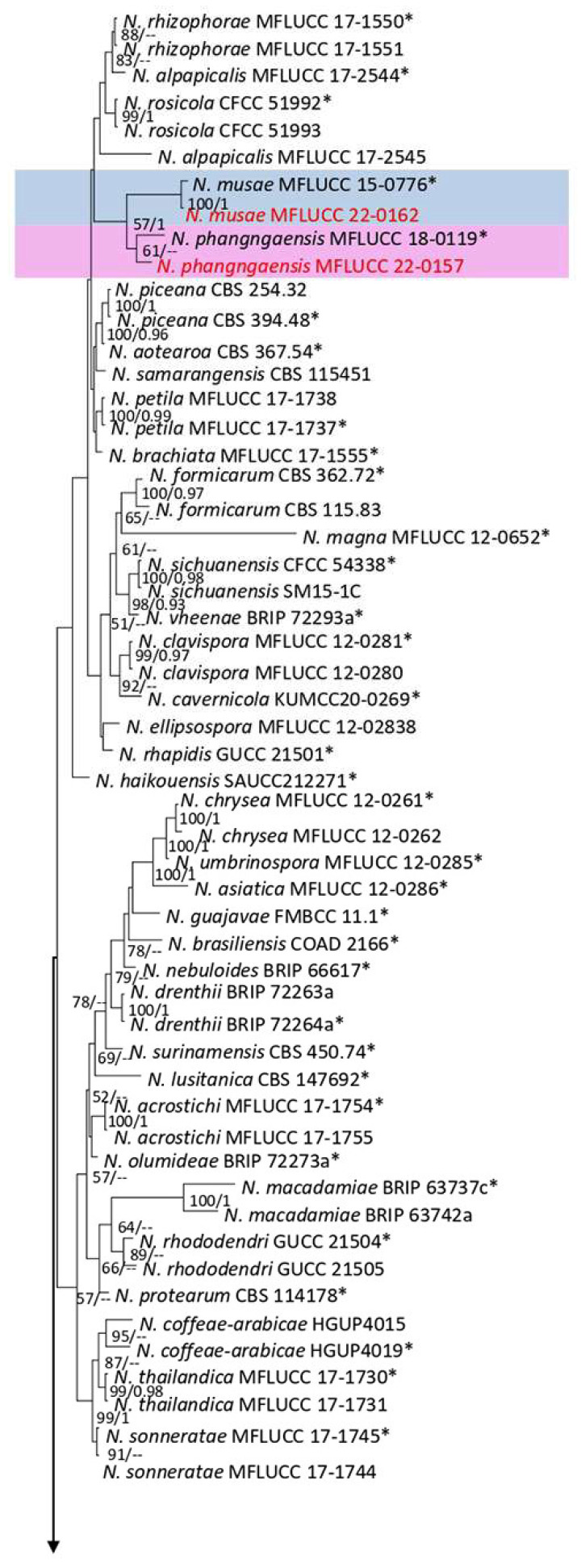

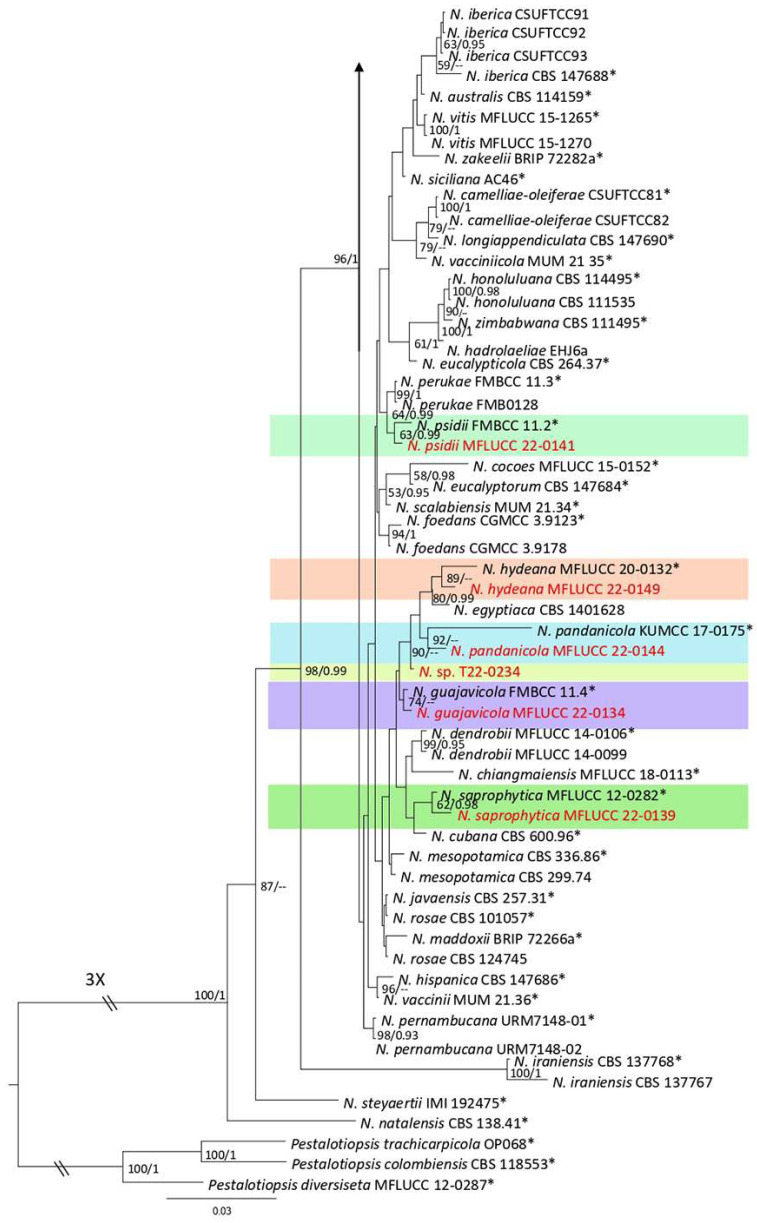
Bayesian phylogenetic tree of *Neopestalotiopsis* spp. Obtained from the combined ITS, *tef1*, and *tub2* sequence data. The ultrafast maximum likelihood bootstrap support (%) ≥ 50 and BYPP ≥ 0.95 are shown at the nodes. The ex-type strains are marked with an asterisk. The tree is rooted in *Pestalotiopsis trachicarpicola* (OP068), *P. colombiensis* (CBS 118553), and *P. diversiseta* (MFLUCC 12-0287).

***Neopestalotiopsis guajavicola*** I.U. Haq, Ijaz & N.A. Khan, 2021.

**Figure 6:**
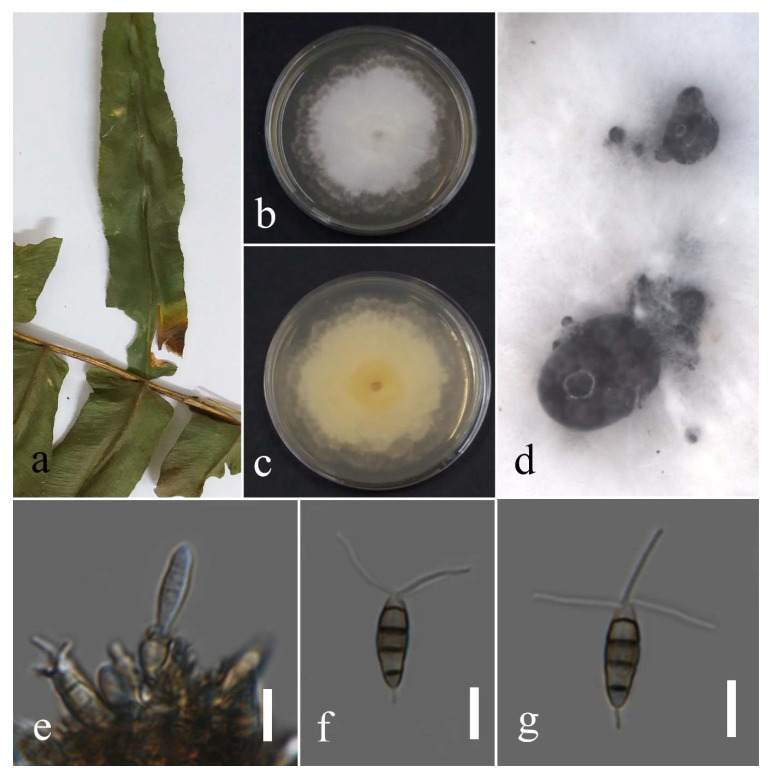
Morphology of *Neopestalotiopsis guajavicola* (MFLUCC 22-0134): (**a**) blight on *Nephrolepis* sp.; (**b**,**c**) upper and reverse views of the culture after seven days of growth on PDA at 28 °C; (**d**) conidiomata; (**e**) conidiogenous cells; (**f**,**g**) conidia. Scale bars = 10 µm.

Facesoffungi number: FoF 13402; Figure 6.

Associated with *Nephrolepis* sp. leaf spot. Sexual morph: not observed. Asexual morph: conidiomata ample, aggregated and scattered, immersed and semi-immersed, exuding black conidial mass. Conidiophores reduced to conidiogenous cells. Conidiogenous cells subcylindrical to ampliform, hyaline to pale brown. Conidia fusiform, straight or curved, 4 septate, 17–22 × 4–8 µm (mean = 20.1 × 6.5, n = 30). Basal cell conoid with truncate base, hyaline, 2–4 µm long; median cells, versicolored, darker spectate than other cells, 12–16 × 5–8 µm (mean = 14.3 × 6.4, n = 30) (second cell from the base pale brown, 3.5–5.9 µm long; third cell from the base medium brown, 3–7 µm long; forth cell from the base pale to medium brown, 3–6 µm long); apical cell conic, hyaline, 2–5 µm with 2–3 tubular apical appendage (often three) filiform, hyaline, unbranched, and 11–21 µm long (mean = 16.1, n = 30); basal appendage filiform, hyaline, unbranched, solitary, and 1–7 µm long. 

Culture characteristics: The colonies reached 72–80 mm after seven days of growth on PDA at 28 °C; cottony, irregular shape, dull surface, undulated edge, fluffy margin, medium density, and without pigmentation in the medium and conidial mass. Upper view is white, reverse pale luteous in the center, and primrose in other areas. 

Material examined: Thailand, Chiang Rai Province, Muang District, Thasud, on leaf spot of *Nephrolepis* sp. (*Nephrolepidaceae*), 4 December 2021, Elaheh Seifollahi, dried culture (MFLU 22-0210) and living culture MFLUCC 22-0134.

Notes: Isolated from a blight on leaves of *Nephrolepis* sp. The obtained isolate (MFLUCC 22-0134) clustered with *N. guajavicola* with a 74% ML bootstrap (Figure 5). Comparison of the DNA sequences of *N. guajavicola* strains (ex-type FMBCC 11.4 and MFLUCC 22-0134) revealed 0.59% nucleotide differences in *tef1* (1 gap) and 0.24% in *tub2* (one nucleotide) genes, while the sequences of ITS were identical. This species was first reported from a guava tree in Pakistan [29] and, here, it was isolated from *Nephrolepis* sp. In Thailand. Herein, we provided a new host and a geographical record for *N. guajavicola*. 

***Neopestalotiopsis hydeana*** Huanraluek & Jayaward., 2021.

**Figure 7:**
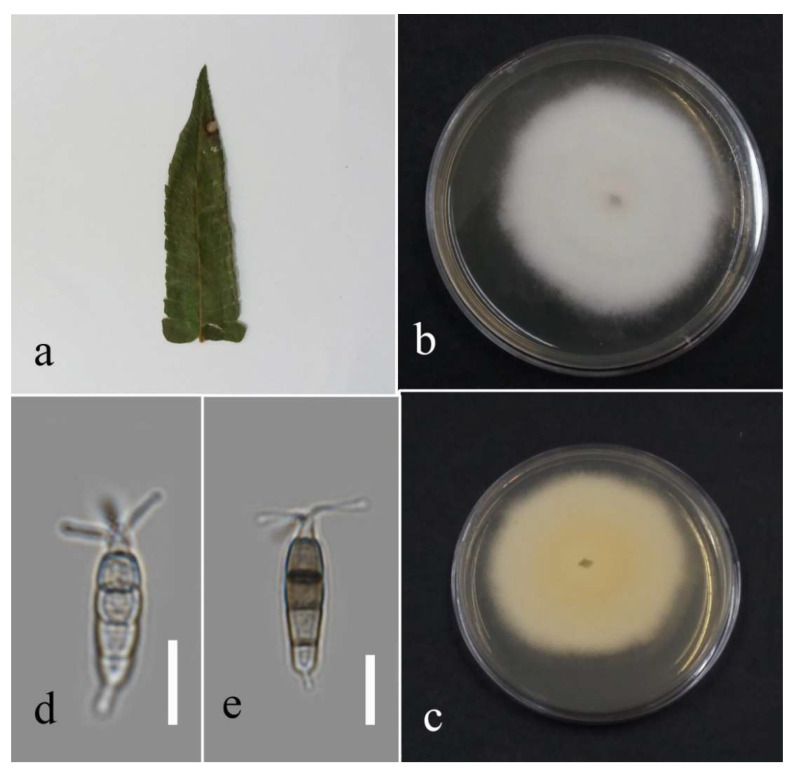
Morphology of *Neopestalotiopsis hydeana* (MFLUCC 22-0149): (**a**) necrotic spot in *Cyclosorus* sp.; (**b**,**c**) upper and reverse views of the colony after seven days of growth on PDA at 28 °C; (**d**) immature conidium; (**e**) mature conidium. Scale bars = 10 µm.

Facesoffungi number: FoF 09459; Figure 7.

Associated with leaf spot of *Cyclosorus* sp. Sexual morph: not observed. Asexual morph: conidiomata not observed. Conidia were found rarely, fusiform, straight, 4 septate, 19–27 × 4–7 µm (mean = 23.3 × 6.2, n =20). Basal cell conoid with a truncated base, hyaline, 3.9–5.4 µm; median cell versicolored, darker septate than other cells, 9–17 × 4–9 µm (mean = 15.1 × 6.0, n = 20) (second cell from the base pale brown, 4–7 µm long; third cell medium brown, 4–6 µm long; forth cell medium brown, 3–6 µm long); apical cell conoid, hyaline, 1–5 µm long with 2–3 apical appendages tubular, filiform, hyaline, unbranched, and 4–13 µm long (mean = 9.1, n = 20); basal appendage filiform, hyaline, unbranched, single, and 2–6 µm long.

Culture characteristics: The colonies reached 65–67 mm after seven days of growth on PDA at 28 °C; cottony, entire edge, fluffy margin, medium density, and without pigmentation in the medium and fruiting body. Upper view white and the reverse primrose. 

Material examined: Thailand, Chiang Rai Province, Muang District, Thasud, on leaf spot of *Cyclosorus* sp. (*Thelypteridaceae*), 17 December 2021, Elaheh Seifollahi, dried culture (MFLU 22-0211) and living culture MFLUCC 22-0149.

Notes: Isolated from a necrotic spot with a dark-brown margin. The obtained isolate in this study (MFLUCC 22-0149) clustered with *N. hydeana* by 89% ML bootstrap support (Figure 5). Comparison of the DNA sequences of *N. hydeana* strains (MFLUCC 20-0132 and ex-type strain T22-0333) showed 0.83% nucleotide differences in ITS (three nucleotides and one gap), 0.91% in *tef1* (four nucleotides), and 0.26% in *tub2* (one nucleotide). This species was isolated from fruit rot in *Annona squamosal* and *Garcinia mangostana* and leaf spots on *Alpinia malaccensis* and *Garcinia mangostana* in Thailand [30] and, here, it was isolated from *Cyclosorus* sp. Hence, we provide a new host record for *N. hydeana*. 

**Figure 8:**
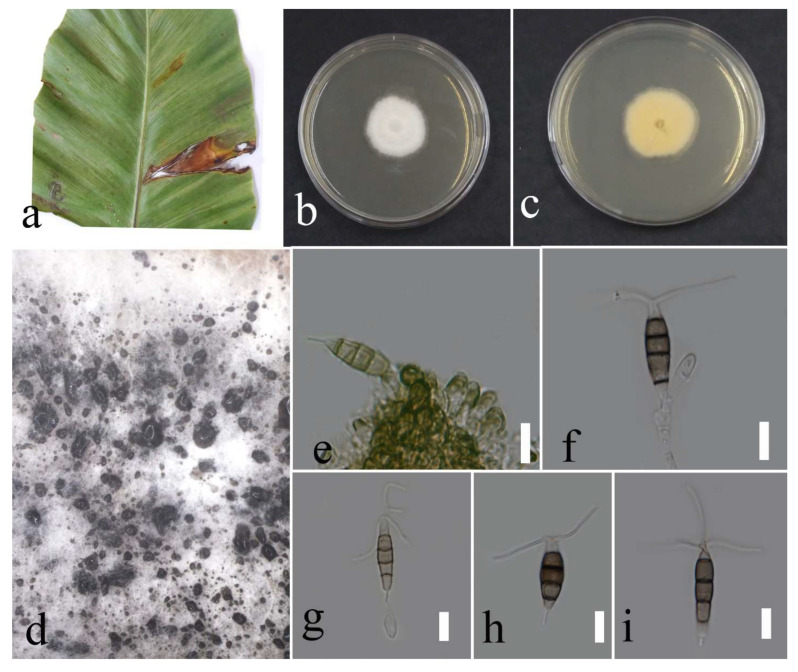
Morphology of *Neopestalotiopsis musae* based on the isolate MFLUCC 22-0162: (**a**) leaf spot in *Asplenium nidus*; (**b**,**c**) upper and reverse views of the colony after seven days of growth on PDA at 28 °C; (**d**) conidiomata; (**e**,**f**) conidiogenous cells; (**g**–**i**) conidia. Scale bars = 10 µm.

***Neopestalotiopsis musae*** Norphanph., T.C. Wen & K.D. Hyde, 2016; Figure 8.

Associated with *Asplenium nidus* leaf spot. Sexual morph: not observed. Asexual morph: conidiomata ample, scattered, and aggregated, immersed and semi-immersed, with black conidial mass. Conidiophores reduced to conidiogenous cells. Conidiogenous cells subcylindrical to ampulliform and hyaline to medium brown. Conidia fusiform, straight to slightly curved, 4 septate, 19–26 × 5–9 µm (mean = 22.7 × 6.9, n = 30). Basal cell conical, hyaline to pale brown, and 3–6 µm; three median cells doliiform, versicolored, and darker septate than the other cells, 12–17 × 5–9 µm (mean = 14.55 × 6.75, n = 30) (second cell from the base pale brown, 3–7 µm long; third cell pale to medium brown, 3–7 µm long; forth cell pale to medium brown, 3–6 µm long); apical cell conical, hyaline, 3–6 µm long with 2–3 apical appendages (often, two appendages), filiform, hyaline, unbranched, and 13–28 µm long (mean = 22.5, n = 30); basal appendage filiform, hyaline, unbranched, singular, and 3–7 µm long. 

Culture characteristics: Colonies reached 31–33 mm after seven days of growth on PDA at 28 °C; felted to cottony, dull surface, entire edge, regular and fluffy margin, medium density, and without pigmentation in the medium and fruiting body. Upper view white and the reverse primrose to straw. 

Material examined: Thailand, Chiang Rai Province, Muang District, Huai Sak, on leaf spot of *Asplenium nidus*, 17 December 2021, Elaheh Seifollahi, dried culture (MFLU 22-0212) and living culture MFLUCC 22-0162.

Notes: Isolated from blights on leaves of *Asplenium nidus*. The isolate obtained in this study (MFLUCC 22-0162) grouped with *N. musae* in the same clade by 100% ML bootstrap support and 1.0 BYPP (Figure 5). Comparison of the DNA sequences of *N. musae* strains (MFLUCC 15-0776 and ex-type strainT22-0164) showed 0.21% nucleotide differences in the ITS (1 gap), 0.49% in *tub2* (1 nucleotide and 1 gap) genomic regions, while the *tef1* sequences were identical. This species was first reported from *Musa* sp. in Thailand [31] and, here, it was isolated from *Asplenium nidus*, providing a new host record. 

***Neopestalotiopsis pandanicola*** Tibpromma & K.D. Hyde, 2018.

**Figure 9:**
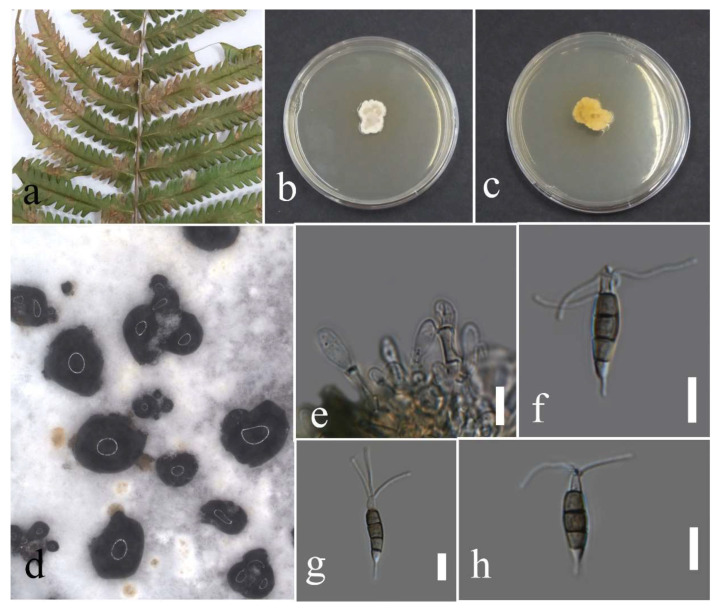
Morphology of *Neopestalotiopsis pandanicola* based on the isolate MFLUCC 22-0144: (**a**) leaf spot in *Cyclosorus* sp.; (**b**,**c**) upper and reverse views of the colony after seven days of growth on PDA at 28 °C; (**d**) conidiomata; (**e**) conidiogenous cell; (**f**–**h**) conidia. Scale bars = 10 µm.

Facesoffungi number: FoF 13403; Figure 9.

Associated with leaf spot. Sexual morph: Not observed. Asexual morph: Conidiomata ample, scattered, and aggregated, immersed and semi-immersed with black conidial mass. Conidiophores reduced to conidiogenous cells. Conidiogenous cells subcylidrical to ampulliform, hyaline. Conidia fusiform, straight to slightly curved, 4-septate, 16–25 µm × 4–7 µm (mean = 21.1 × 5.5, n= 30). Basal cell conical, hyaline, 3–7 µm long; three median cells doliform, concoloured, pale to medium brown, darker septate than the other cells, and 10–16 × 4–7 µm (mean = 13.6 × 5.4, n = 30) (the second cell from the base 4–6 µm long; the third cell 3–6 µm long; and the fourth cell 3–7 µm); apical cell conical to cylindrical, hyaline, 3–7 µm long, with 2–3 apical appendages, filiform, hyaline, unbranched, and 7–30 µm long (mean = 17.6, n = 30); basal appendage filiform, hyaline, unbranched, single, and 3–7 µm long. 

Culture characteristics: Colonies reached 16–21 mm after seven days of growth on PDA at 28 °C; felted, dull surface, irregular shape, crenate edge, and wrinkled aspect, dense density, and without pigmentation in the medium and fruiting body. Upper view olivaceous buff in center and white in other areas, and reverse pale luteous with ochreous parts. 

Material examined: Thailand, Chiang Rai Province, Muang District, Thasud, on leaf spot of *Cyclosorus* sp., 4 December 2021, Elaheh Seifollahi, (MFLU 22-0213), living culture MFLUCC 22-0144.

Notes: Isolated from a necrotic spot with a brown margin on *Cyclosorus* sp. The isolate obtained in this study (MFLUCC 22-0144) clustered with *N. pandanicola* in the same clade by 92% ML bootstrap support (Figure 5). Comparison of the DNA sequences of the ex-type strain of *N. pandanicola* (KUMCC 17-0175) with strain MFLUCC 22-0144 revealed 4.29% nucleotide differences in *tef1* (11 nucleotides and eight gaps) and 0.26% in *tub2* (1 nucleotide) genes. The ITS sequence for the type strain was not available. This species was introduced from *Pandanus* sp. in China [32] and, here, it was isolated from *Cyclosorus* sp. in Thailand, providing a new host and geographical records.

***Neopestalotiopsis phangngaensis*** Tibpromma & K.D. Hyde, 2018.

**Figure 10:**
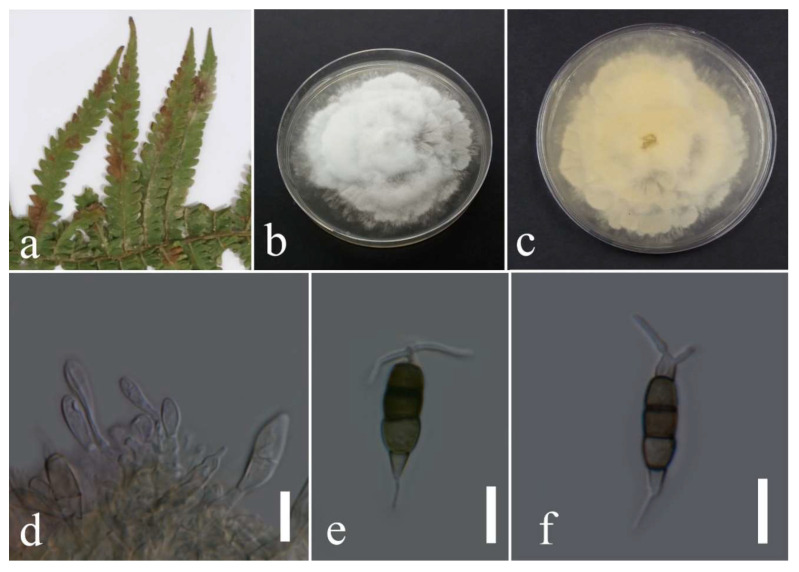
Morphology of *Neopestalotiopsis phangngaensis* (MFLUCC 22-0157): (**a**) brown leaf spot in *Cyclosorus* sp.; (**b**,**c**) upper and reverse views of the colony after seven days of growth on PDA at 28 °C; (**d**) conidiogenous cells; (**e**,**f**) conidia. Scale bars = 10 µm.

Facesoffungi number: FoF 04527; Figure 10.

Associated with *Cyclosorus* sp. leaf spot. Sexual morph: not observed. Asexual morph: conidiomata rarely formed, scattered, semi-immersed, with black conidial mass. Conidiophores reduced to conidiogenous cells. Conidiogenous cells subcylindrical to ampulliform, hyaline, entroblastic, and thin walled. Conidia fusiform, straight or slightly curved, 4 septate, and 20–26 × 4–8 µm (mean = 22.6 × 6.0, n = 30); basal cell conical, hyaline to pale brown, 3–6 µm long; three median cells doliiform, versicolored with darker septate than the rest of the cells, and 12–17 µm × 4–8 (mean = 14.7 × 6.1, n = 30) (second cell from the base pale brown, 3–6 µm long; third cell medium brown, 4–7 µm long; forth cell pale to medium brown, 2–7 µm long), apical cell conical, hyaline, 3–6 µm long with 2–3 apical appendages, filiform, hyaline, unbranched, and 8–26 µm long; basal appendage filiform, hyaline, unbranched, singular, and 4–8 µm long. 

Culture characteristics: Colonies reached 75–90 mm after seven days of growth on PDA at 28 °C; fluffy to cottony, irregular shape, dull surface, undulate edge, fluffy margin, wrinkled folded aspect in some areas, medium density and without pigmentation in medium and conidial mass. Upper view white and the reverse primrose.

Material examined: Thailand, Chiang Rai Province, Muang District, Thasud, on leaf spot of *Cyclosorus* sp., 17 December 2021, Elaheh Seifollah, dried culture (MFLU 22-0214) and living culture (MFLUCC 22-0157).

Notes: Isolated from brown leaf spots on *Cyclosorus* sp. The isolate obtained in this study (MFLUCC 22-0157) grouped with *N. phangngaensis* in the same cluster by 61% ML bootstrap support (Figure 5). In comparing the DNA sequences of the ex-type strain (MFLCC 18-0119) with MFLUCC 22-0157, the *tef1* for the type strain was removed in analyses because of ambiguities in the sequence. Comparison of the DNA sequences showed 0.22% nucleotide differences in ITS (one gap) and 0.51% in *tub2* (two nucleotides) genomic regions. *N. phangngaensis* was first isolated from dead leaves of *Pandanus* sp. in Thailand [32] and, here, it was recorded on *Cyclosorus* sp., providing a new host occurrence. 

***Neopestalotiopsis psidii*** I.U. Haq, Ijaz & N.A. Khan, 2021.

**Figure 11:**
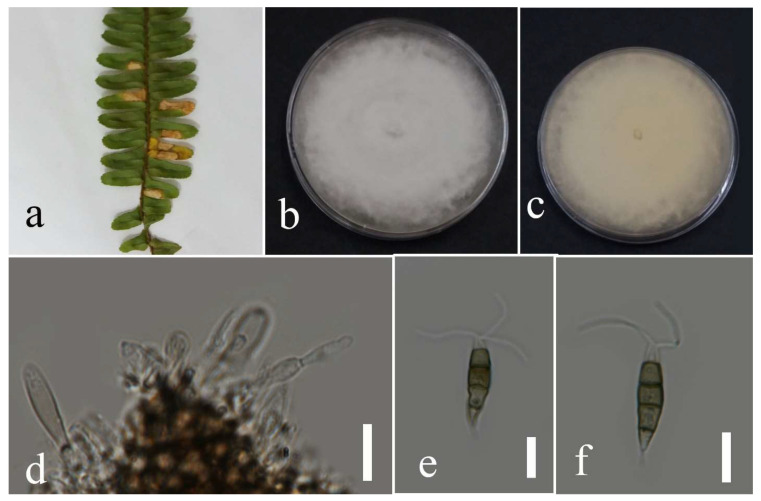
Morphology of *Neopestalotiopsis psidii* based on isolate MFLUCC 22-0141: (**a**) leaf spot in *Nephrolepis cordifolia*; (**b**,**c**) upper and reverse views of the colony after seven days of growth on PDA at 28 °C; (**d**) conidiogenous cells; (**e**,**f**) conidia. Scale bars = 10 µm.

Facesoffungi number: FoF 13404; Figure 11.

Associated with *Nephrolepis cordifolia* leaf spot. Sexual morph: not observed. Asexual morph: conidiomata ample, scattered, or aggregated, immersed and semi-immersed, with black conidial mass. Conidiophores reduced to conidiogenous cells. Conidiogenous cells cylindrical to ampulliform, hyaline, entroblastic, and thin walled. Conidia fusiform, straight to slightly curved, 4-septate, 17–30 µm × 4–8 µm (mean = 22.2 × 6.1, n = 30); basal cell conical, hyaline, thin walled, 2–5 µm long; three median cells doliiform, versicolored with darker septate than the other cells, 12–19 µm × 5–8 (mean = 14.6 × 6.5, n = 30) (second cell from the base pale brown, 3–7 µm long; third cell medium brown, 2–6 µm long; forth cell pale to medium brown, 3–6 µm long); apical cell conical, hyaline, with thin wall, 2–5 µm long with 2–3 apical appendages filiform, hyaline, unbranched, 10–19 µm (mean = 13.99, n = 30); basal appendage filiform, hyaline, unbranched, singular, 1–5 µm long. 

Culture characteristics: Colonies filled 90 mm Petri dish after seven days of growth on PDA at 28 °C, cottony, dull surface, irregular margin, undulate edge, medium density, and without pigmentation in medium and conidial mass. Upper view white, and reverse primrose. 

Material examined: Thailand, Chiang Rai Province, Muang District, Thasud, on leaf spot of *Nephrolepis cordifolia*, 4 December 2021, Elaheh Seifollah, dried culture (MFLU 22-0215), living culture MFLUCC 22-0141. 

Notes: Isolated from a necrotic leaf spot with a brown margin on *Nephrolepis cordifolia*. The isolate obtained in this study (MFLUCC 22-0141) clustered with *N. psidii* in the same clade by 63% ML bootstrap support and 0.99 BYPP (Figure 5). Comparison of the DNA sequences of *N. psidii* strains (FMBCC 11.2 and ex-type strain T22-0247) revealed 2.13% nucleotide differences in *tef1* (seven nucleotides and one gap) gene, while the sequences of ITS and *tub2* were identical. This species was first reported from guava in Pakistan [29] and, here, it was isolated from *Nephrolepis cordifolia* in Thailand as a new host and geographical record.

***Neopestalotiopsis saprophytica*** Maharachch., K.D. Hyde & Crous, 2014.

**Figure 12:**
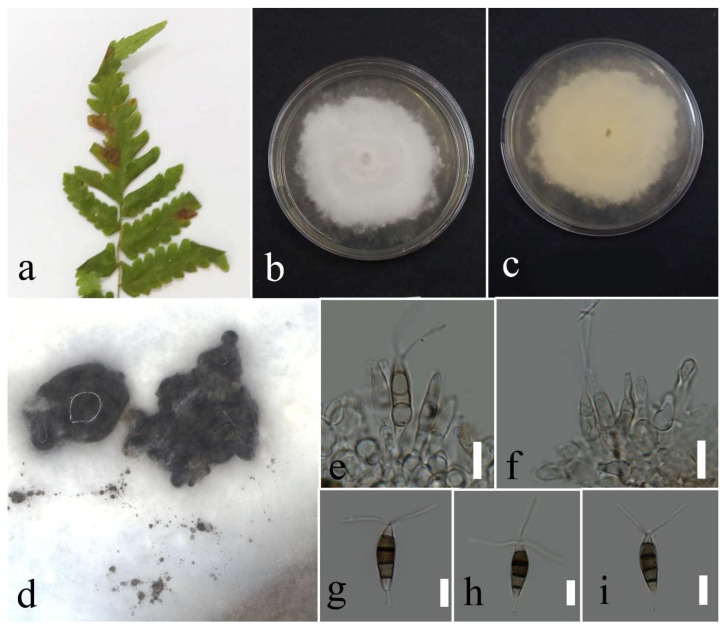
Morphology of *Neopestalotiopsis saprophytica* (MFLUCC 22-0139): (**a**) brown leaf spot in *Cyclosorus* sp.; (**b**,**c**) upper and reverse views of the colony after seven days of growth on PDA at 28 °C; (**d**) coidiomata; (**e**,**f**) conidiogenous cells; (**g**–**i**) conidia. Scale bars = 10 µm.

Facesoffungi number: FoF 13405; Figure 12.

Associated with leaf spot. Sexual morph: not observed. Asexual morph: conidiomata ample, scattered, and aggregated, semi-immersed with black conidial mass. Conidiophores reduced to conidiogenous cells. Conidiogenous cells lageniform, cylindrical to subcylindrical, and hyaline. Conidia fusiform, straight to slightly curved, 4 septate, 17–29 × 5–8 µm (mean = 21.9 × 6.8, n = 30); basal cell conical, hyaline to pale brown, thin walled, 3–6 µm long; three median cells doliiform, versicolored, and darker septate than the other cells, and 11–18 × 5–8 µm (mean = 14.5 × 6.5, n = 30) (second cell from the base pale brown to olivaceous, 3–6 µm long; third cell from base medium to dark brown, 2–6 µm long; forth cell medium to dark brown, 3–6 µm long); apical cell conical, hyaline, thin walled, 3–6 µm long with 2–3 apical appendages (mostly 3), tubular, hyaline, unbranched, and 13–26 µm (mean = 19.4, n = 30) long; basal appendage filiform, hyaline, unbranched, singular, and 2–10 µm long. 

Culture characteristics: Colonies reached 72–80 mm after seven days of growth on PDA at 28 °C; cottony irregular shape, dull surface, undulate edge, medium density, and without pigmentation in the medium and conidial mass. Upper view white and the reverse primrose. 

Material examined: Thailand, Chiang Rai Province, Muang District, Thasud, on leaf spot of *Cyclosorus* sp., 4 December 2021, Elaheh Seifollahi, dried culture (MFLU 22-0216) and living culture MFLUCC 22-0139.

Notes: Isolated from a brown leaf spot on *Cyclosorus* sp. The isolate obtained in this study (MFLUCC 22-0139) clustered with *N. saprophytica* with 62% ML bootstrap support and 0.98 BYPP (Figure 5). Comparison of the DNA sequences of *N. saprophytica* strains (ex-type strain MFLUCC 12-0282 and MFLUCC 22-0139) revealed 1.03% nucleotide differences in ITS (five nucleotides) and 0.90% in *tef1* (four gaps), while the *tub2* sequences were identical. The mentioned species was first reported from *Litsea rotundifolia* and *Magnolia* sp. in China [28] and, here, it was isolated from *Cyclosorus* sp. in Thailand as the new host and geographical records. 

***Pestalotiopsis*** Steyaert, *Bulletin du Jardin Botanique de l’État à Bruxelles*, 19 (3): 300 (1949).

The species of this genus occur as endophytes, saprobes, and opportunistic pathogens on various plants. *Pestalotiopsis* is distinguished from *Nepestalotiopsis* by concolorous median cells of the conidia [28]. In the current study, *P. hydei* and *P. dracontomelon* were obtained from leaf spots on ferns by morphological characters and phylogeny of the ITS, *tub2*, and *tef1* gene regions. 

**Figure 13:**
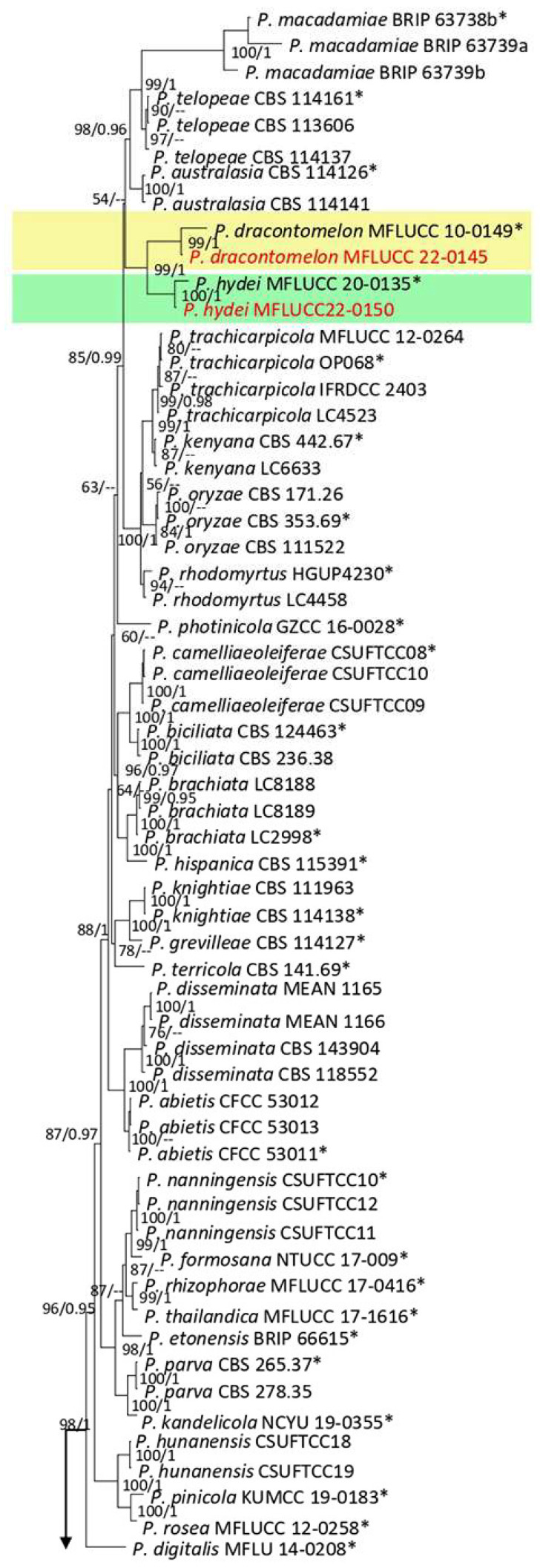

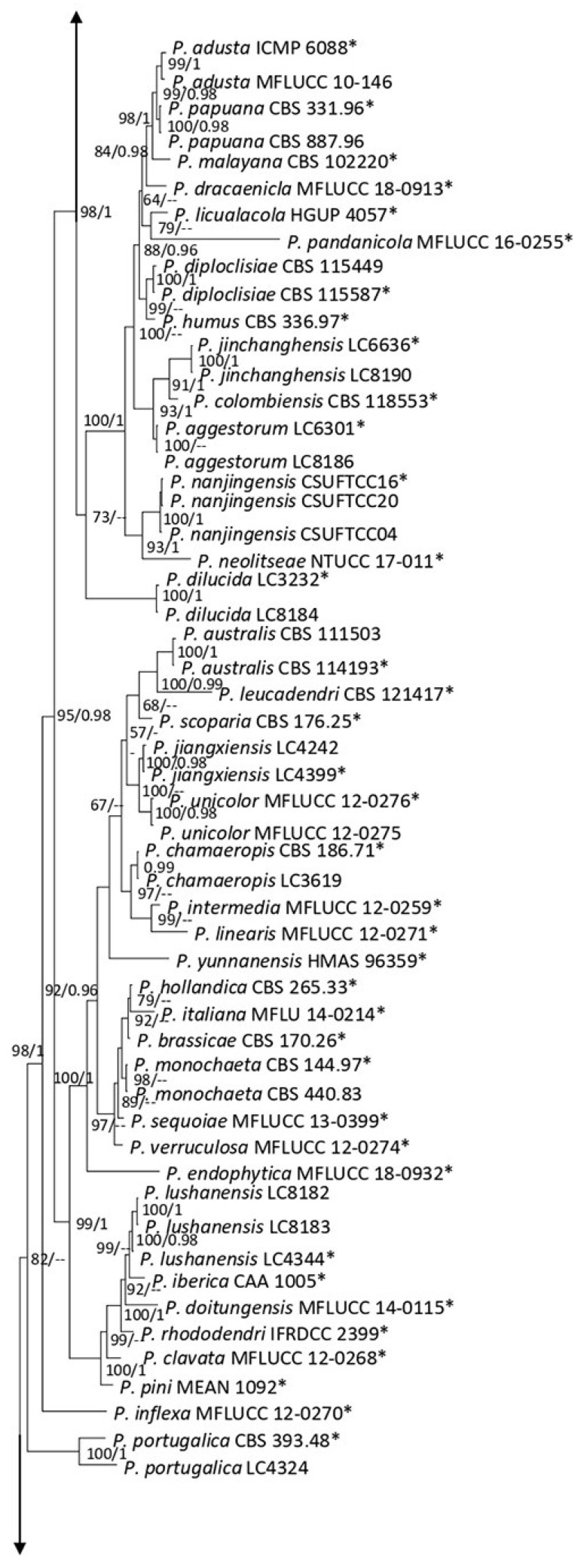

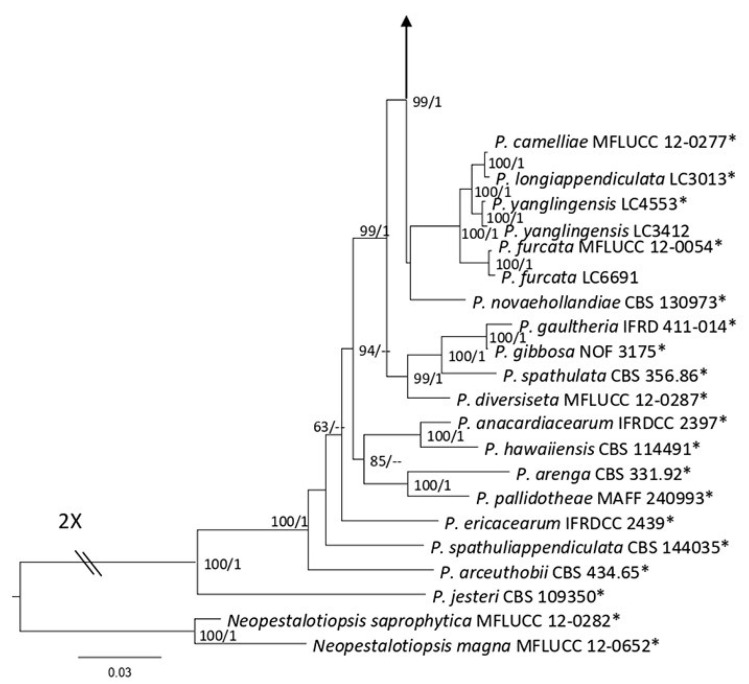
Bayesian phylogenetic tree of *Pestalotiopsis* spp. obtained from the combined ITS, *tef1*, and *tub2* sequence data. The ultrafast maximum likelihood bootstrap support (%) ≤ 50 and BYPP ≥ 0.95 are shown at the nodes. The ex-type strains are marked with an asterisk. The tree is rooted in *Neopestalotiopsis saprophytica* (MFLUCC 12-0282) and *N. magna* (MFLUCC 12-0652).

***Pestalotiopsis dracontomelon*** Maharachch. & K.D. Hyde, 2015.

**Figure 14:**
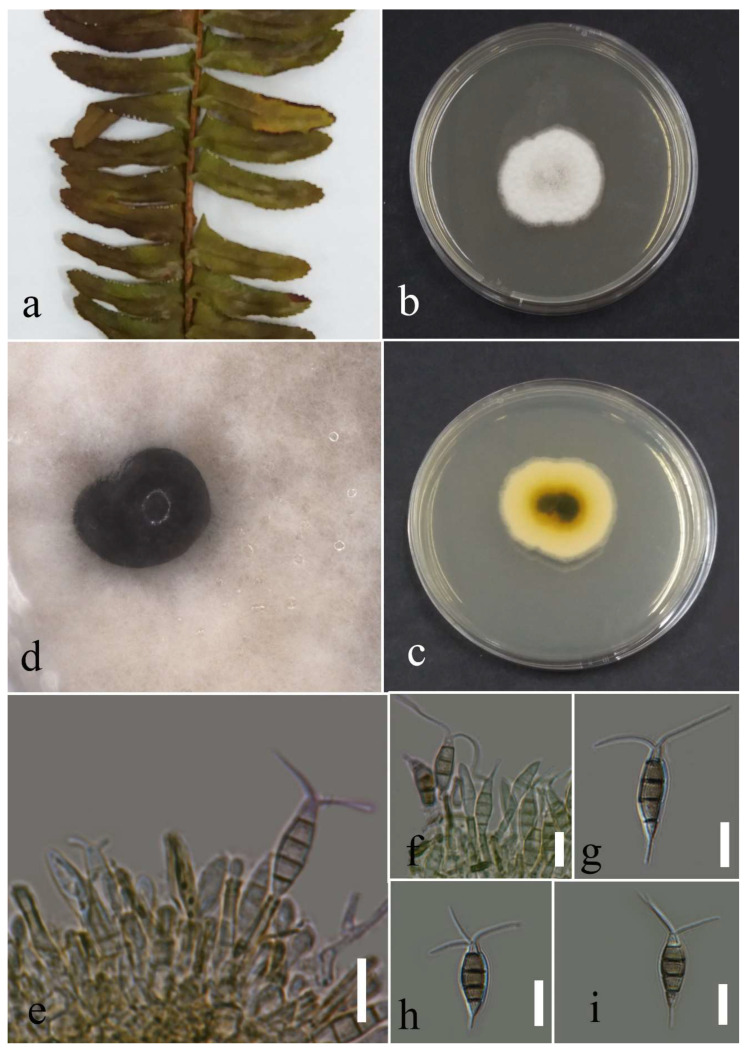
Morphology of *Pestalotiopsis dracontomelon* (MFLUCC 22-0145): (**a**) blight in *Nephrolepis cordifolia*; (**b**,**c**) upper and reverse views of the colony after seven days of growth on PDA at 28 °C; (**d**) conidiomata; (**e**,**f**) conidiogenous cells; (**g**–**i**) conidia. Scale bars = 10 µm.

Facesoffungi number: FOF00457; Figure 14.

Associated with *Nephrolepis cordifolia* leaf spot. Sexual morph: not observed. Asexual morph: conidiomata a few, scattered, semi-immersed to immersed, with black conidial mass. Conidiophores reduced to conidiogenous cells. Conidiogenous cells cylindrical, hyaline to pale brown, entroblastic. Conidia fusiform, straight to slightly curved, 4 septate, 16–22 × 4–7 µm (mean = 19.8 × 6.0, n = 30); basal cell conical, hyaline, thin walled, and 3–5 µm long; three median cells doliiform, concoloured, olivaceous, darker septate than the other cells, and 10–15 µ × 4–8 µm (mean = 12.9 × 6.0, n = 30) (second cell from the base 3–5 µm long; third cell 3–6 µm long; and forth cell 2–6 µm long); apical cell conical, hyaline, thin walled, and 2–5 µm long with 2–3 apical appendages filiform, hyaline, unbranched, and 7–20 µm long (mean = 13.7, n = 30); basal appendage tubular, hyaline, unbranched, singular, and 1–8 µm long. 

Culture characteristics: Colonies reached 32–36 mm after seven days of growth on PDA at 28 °C; felted, regular and entire edge, dull surface and well-defined margin, medium density and without pigmentation in medium and conidial mass. Upper view white, and the reverse greenish black, buff, and primrose circles from center to margin. 

Material examined: Thailand, Chiang Rai Province, Muang District, Thasud, on leaf spot of *Nephrolepis cordifolia*, 4 December 2021, Elaheh Seifollahi, dried culture (MFLU 22-0217) and living culture MFLUCC 22-0145.

Notes: Isolated from a blight on leaves’margin of *Nephrolepis cordifolia*. The isolate obtained in this study (MFLUCC 22-0145) clustered with *P. dracontomelon* in the same clade by 99% ML bootstrap support and 1.0 BYPP (Figure 13). Comparison of the DNA sequences of *P. dracontomelon* strains (ex-type strain MFLUCC 10-149 with MFLUCC 22-0145) showed 1.02% nucleotide differences in ITS (five nucleotides) and 0.23% in *tef1* (one gap). Sequence data for *tub2* were unavailable for the type strain (MFLUCC 10-149). This species was introduced from *Dracontomelon dao* in Thailand [33]. Here, we provide a new host record for *P. dracontomelon*.

***Pestalotiopsis hydei*** Huanraluek & Jayaward., 2021. 

**Figure 15:**
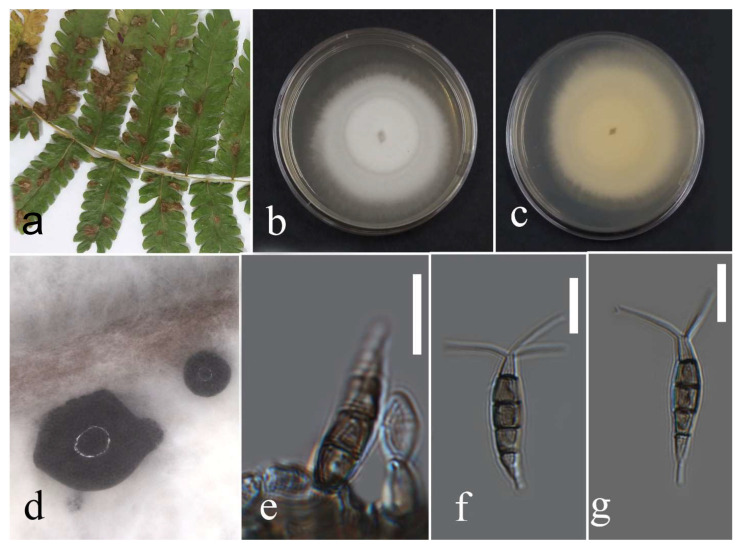
Morphology of *Pestalotiopsis hydei* based on isolate MFLUCC 22-0150: (**a**) leaf spot in *Cyclosorus* sp.; (**b**,**c**) upper and reverse views of the colony after seven days of growth on PDA at 28 °C; (**d**) conidiomata; (**e**) conidiogenous cell; (**f**,**g**) conidia. Scale bars = 10 µm.

Facesoffungi number: FoF 09460; Figure 15.

Associated with *Cyclosorus* sp. leaf spot. Sexual morph: not observed. Asexual morph: conidiomata a few, scattered, immersed, or semi-immersed, with black conidial mass. Conidiphores reduced to conidiogenous cells. Conidiogenous cells lageniform to cylindrical, hyaline, and entroblastic. Conidia fusiform, straight to slightly curved, 4 septate, and 18–28 × 4–7 µm (mean = 23.6 × 5.7, n = 30); basal cell conical, hyaline, thin walled, and 3–6 µm long; three median cell doliiform, concolourous, olivaceous, darker septate than the other cells, and 13–18 × 4–7 µm (mean = 15.6 × 5.6, n = 30) (second cell from the base 3–7 µm long; third cell 3–7 µm long; and forth cell 4–7 µm long); apical cell conical, hyaline, thin walled, 3–6 µm long with 2–3 apical appendages tubular, hyaline, unbranched, and 5–13 (mean = 9.4, n = 30); basal appendage filiform, hyaline, unbranched, singular, and 2–8 µm. 

Culture characteristics: Colonies reached 73–82 mm after seven days of growth on PDA at 28 °C; felted, circular shape, dull surface, entire edge, fluffy margin, medium dense density, and without pigmentation in medium and conidial mass. Upper view white and the reverse primrose. 

Material examined: Thailand, Chiang Rai Province, Muang District, Thasud, on leaf spot of *Cyclosorus* sp., 4 December 2021, Elaheh Seifollahi, dried culture (MFLU 22-0218) and living culture (MFLUCC 22-0150).

Notes: Th strain MFLUCC 22-0150 was isolated from necrotic brown leaf spots on *Cyclosorus* sp. The isolate obtained in this study (MFLUCC 22-0150) clustered with *P. hydei* in the same cluster by 100% ML bootstrap support and 1.0 BYPP (Figure 13). Comparison of the DNA sequence data of *P. hydei* strains (ex-type strain MFLUCC 20-0135 and MFLUCC 22-0150) revealed 0.62% nucleotide differences in ITS (three nucleotides), while the sequences of *tef1* and *tub2* were identical. This species was first reported from *Litsea petiolata* in Thailand [30] and, here, it was isolated from *Cyclosorus* sp., providing a new host record for this species. 

***Diaporthales*** Nannf., Nova Acta Regiae Societatis Scientiarum Upsaliensis. 8 (2): 53 (1932).

*Diaporthales* comprises 30 families and 181 genera and is characterized by solitary or aggregated perithecia, sometimes with long papilla, unitunicate asci, with a conspicuous refractive ring. The asexual morph is mostly coelomycetes and rarely hyphomycetes [27]. Species of this order can be plant and animal pathogens and are found in the soil and as saprobes or endophytes [34]. 

***Diaporthaceae*** Höhn. ex Wehm., *American Journal of Botany*. 13: 638 (1926).

This family comprised endophytes, pathogens, and saprobes on submerged plants or terrestrials [27]. *Diaporthaceae* is divided into 15 genera: *Apioporthella*, *Apiosphaeria*, *Chaetoconis*, *Chiangraiomyces*, *Diaporthe*, *Hyaliappendispora*, *Leucodiaporthe*, *Massariothea*, *Mazzantia*, *Ophiodiaporthe*, *Paradiaporthe*, *Phaeocytostroma*, *Phaeodiaporthe*, *Pustulomyces*, and *Stenocarpella* [27].

***Diaporthe*** Nitschke, Pyrenomycetes Germanici. 2: 240 (1870).

Diaporthe comprises 13 species complexes and nine singleton species based on phylogenetic analyses of ITS, *tef1*, *tub2*, *cal*, and *his3* [35]. *Diaporthe* species can live on various hosts as saprobes, endophytes, or pathogens [35]. The present study identified four species *(D. chiangraiensis, D. delonicis, D. heveae*, and *D. tectonendophytica)* from the *D. arecae* and *D. sojae complexes* on ferns, based on the morphology and phylogeny of the ITS, *tef1*, *tub2*, *cal*, and *his3* sequence data.

**Figure 16:**
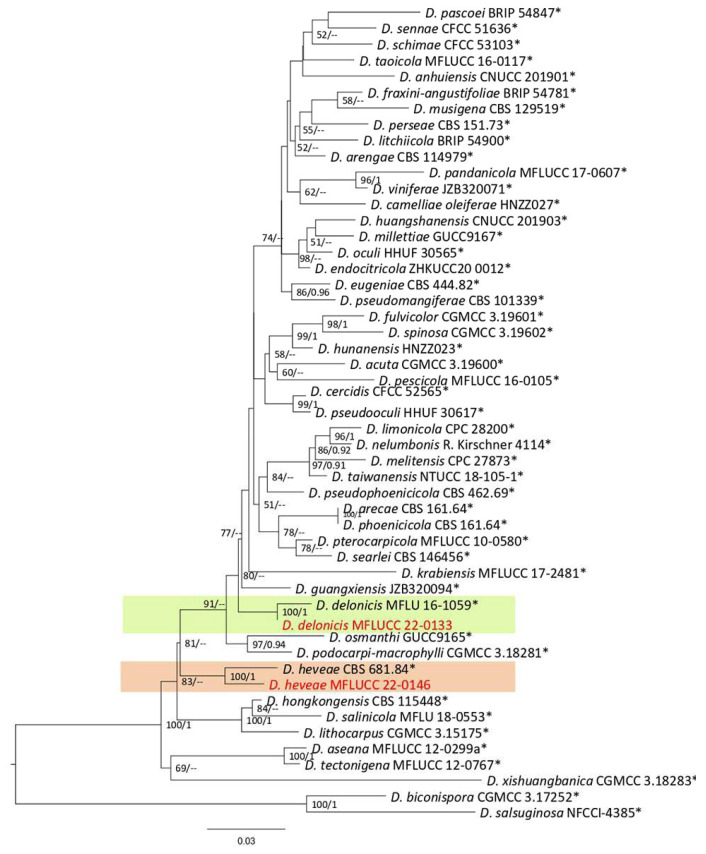
The *Diaporthe arecae* complex maximum likelihood phylogenetic tree obtained from the combined ITS, *tef1*, *tub2*, *cal*, and *his3* sequence data. The ultrafast maximum likelihood bootstrap support (%) ≥ 50 and BYPP ≥ 0.90 are shown at the nodes. The ex-type strains are marked with an asterisk. The tree is rooted in *D. biconispora* (CGMCC 3.17252) and *D. salsuginosa* (NFCCI-4385).

***Diaporthe delonicis*** R.H. Perera, E.B.G. Jones & K.D. Hyde, 2020.

**Figure 17:**
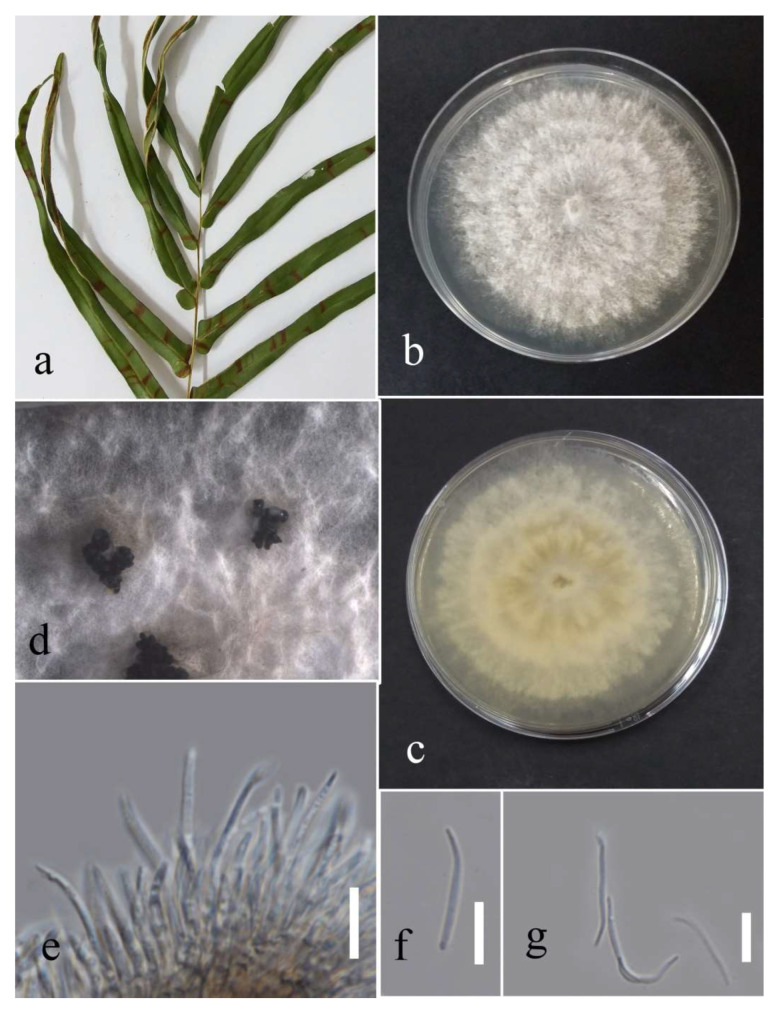
Morphology of *Diaporthe delonicis* (MFLUCC 22-0133): (**a**) leaf brown spots in *Pteris grandifolia*; (**b**,**c**) upper and reverse views of the colony after seven days of growth on PDA at 28 °C; (**d**) pycnidia and conidial mass; (**e**) conidiophores; (**f**,**g**) beta conidia. Scale bars = 10 µm.

Facesoffungi number: FoF 07754; Figure 17.

Associated with *Pteris grandifolia* leaf spot. Sexual morph: not observed. Asexual morph: conidiomata pycnidia, aggregated, semi-immersed, globes to nearly globes, black, with cream conidial mass. Conidiphores subcylindrical, hyaline, and 14–23 × 1–2 µm. *Alfa* conidia absent. Beta conidia filiform, slightly curved at one end, hyaline, aseptate, rounded tips, and 13–25 µm (mean = 18.7, n = 30) × 1–2 µm (mean = 1.5, n = 30).

Culture characteristics: Colonies filled a 90 mm Petri dish after seven days of growth on PDA at 28 °C; fluffy, circular shape, dull surface, undulate edge and fluffy margin, medium sparse density, and without pigmentation in the medium and fruiting body. Upper view with circles of white and olivaceous buff and the reverse primrose with greenish olivaceous areas. 

Material examined: Thailand, Chiang Rai Province, Muang District, Thasud, leaf spot on *Pteris grandifolia*, 3 December 2021, Elaheh Seifollahi, dried culture (MFLU 22-0219) and living culture (MFLUCC 22-0133).

Notes: Isolated from dark-brown spots on *Pteris grandifolia* leaves. The isolate obtained in this study (MFLUCC 22-0133) clustered with *D. delonicis* by 100% ML bootstrap support and 0.98 BYPP (Figure 16). The sequence data of *cal* and *tef1* are not available for the type strain of *D. delonicis*. Comparing sequences of the ex-type strain and our strain (MFLUCC 22-0133) revealed 1.32% nucleotide differences in ITS (six nucleotides) and 0.66% in *tub2* (three nucleotides). This species was first reported from dried seed pods of *Delonix regia* in Thailand [36]. It was isolated from *Pteris grandifolia*, providing a new host record for *D. delonicis*.

***Diaporthe heveae***

**Figure 18:**
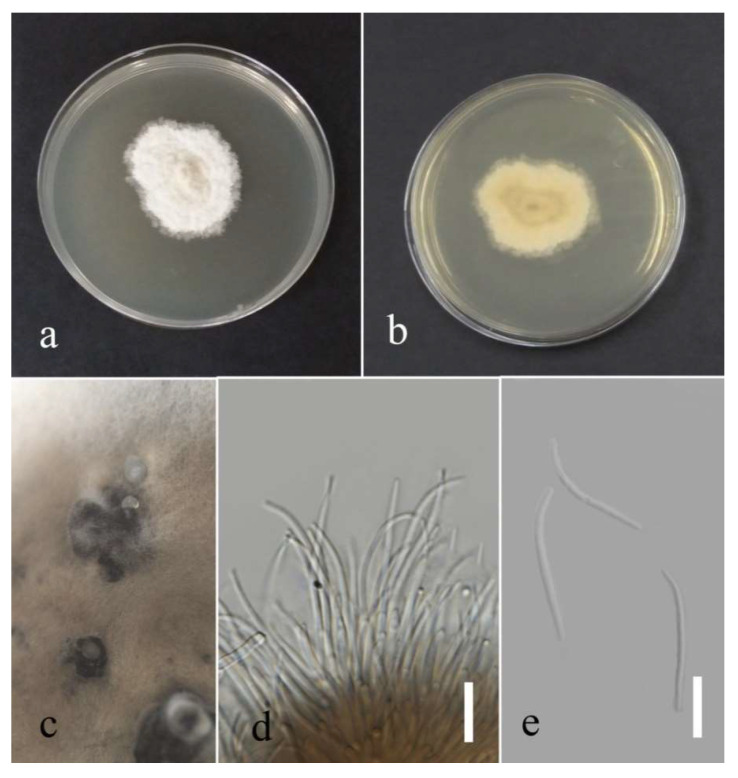
Morphology of *Diaporthe heveae* based on isolate MFLUCC 22-0146: (**a**,**b**) upper and reverse views of the colony after seven days of growth on PDA at 28 °C; (**c**) pycnidia and conidial mass; (**d**) conidiophores; (**e**) beta conidia. Scale bars (**d**,**e**) = 10 µm.

Facesoffungi number: FoF 13406; Figure 18.

Associated with *Nephrolepis cordifolia* leaf spot. Sexual morph: not observed. Asexual morph: conidiomata pycnidia, aggregated, semi-immersed, subglobes, black with cream conidial mass. Conidiophores cylindrical, hyaline, septate, branched and unbranched, rounded at the tip and wider at the base, and 15–38 × 1–2 µm. Alfa and gamma conidia absent. Beta conidia filiform, curved at one tip, hyaline, aseptate, rounded at tips, and 14–30 µm (mean = 25.4, n = 30) × 1–2 µm (mean = 1.4, n = 30). 

Culture characteristics: Colonies reached 33–43 mm after seven days of growth on PDA at 28 °C; felted, dull surface, circular shape, entire edge, fluffy margin, puckered aspect, medium density without pigmentation in the medium and fruiting body. Upper view white with olivaceous buff areas and the reverse primrose and buff areas. 

Material examined: Thailand, Chiang Rai Province, Muang District, Thasud, leaf spot on *Nephrolepis cordifolia*, 3 December 2021, Elaheh Seifollahi, dried culture (MFLU 22-0220), living culture (MFLUCC 22-0146).

Notes: Isolated from a blight on the leaf margin of *Nephrolepis cordifolia*. The isolate obtained in this study (MFLUCC 22-0146) was clustered with *D. heveae* by 100% ML bootstrap support and 1.0 BYPP (Figure 16). The sequence of *his3* is not available for the strain MFLUCC 22-0146 in this study, while the reference sequences of *D. heveae* had this gene region. Comparison of the sequence data of the reference strain for *D. heveae* with our strain (MFLUCC 22-0146) showed 2.87% nucleotide differences in *cal* (7 nucleotides and four gaps), 1.10% in ITS (5 nucleotides), 3.22% in *tef1* (10 nucleotides), and 3.36% in *tub2* (15 nucleotides). This species was first reported from *Hevea brasiliensis* as a die-back agent of the seedlings of the mentioned host in Thailand, Brazil, China, Indonesia, Malaysia, and Sri Lanka [37]. Here, it is first reported in *Nephrolepis cordifolia*.

**Figure 19:**
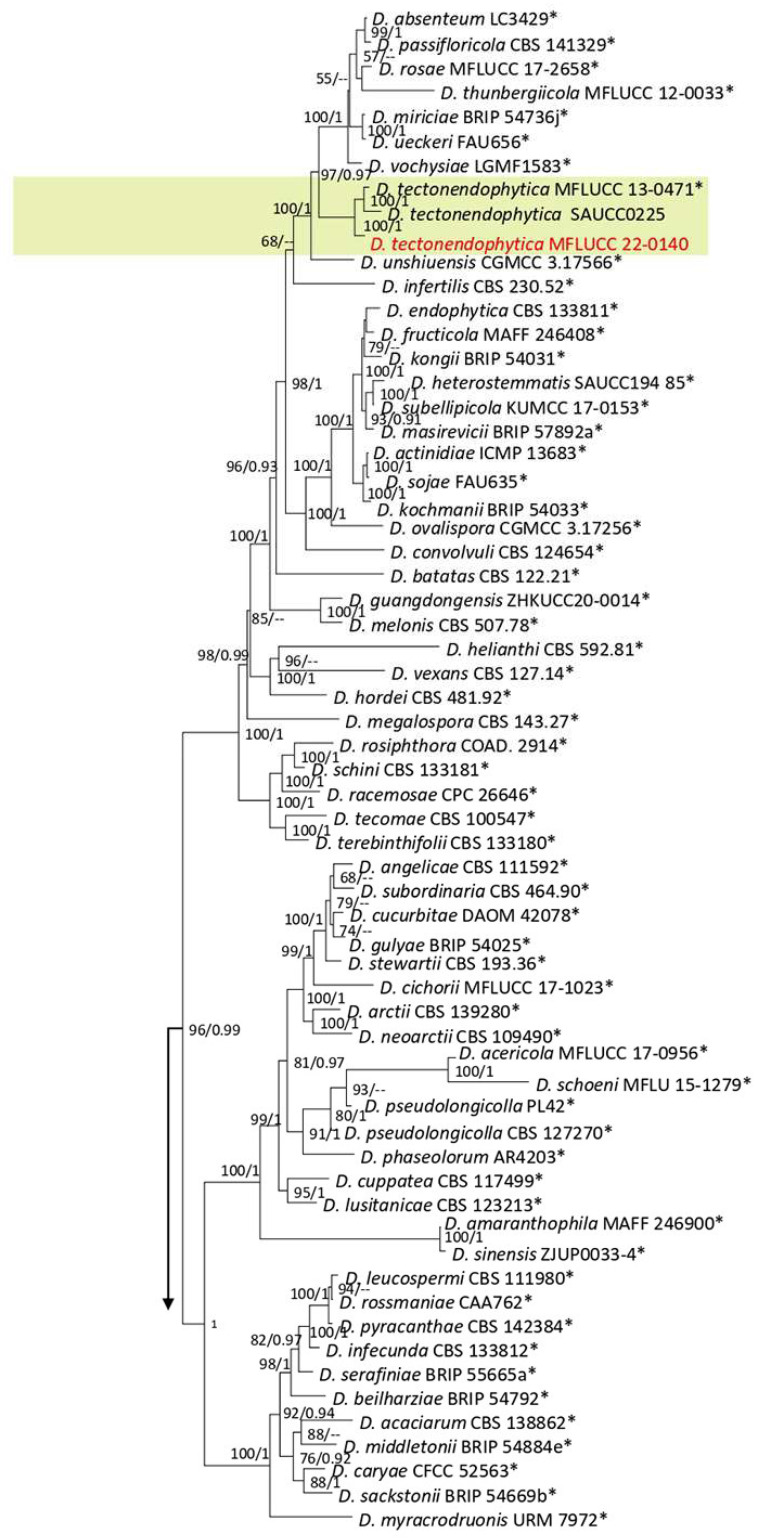

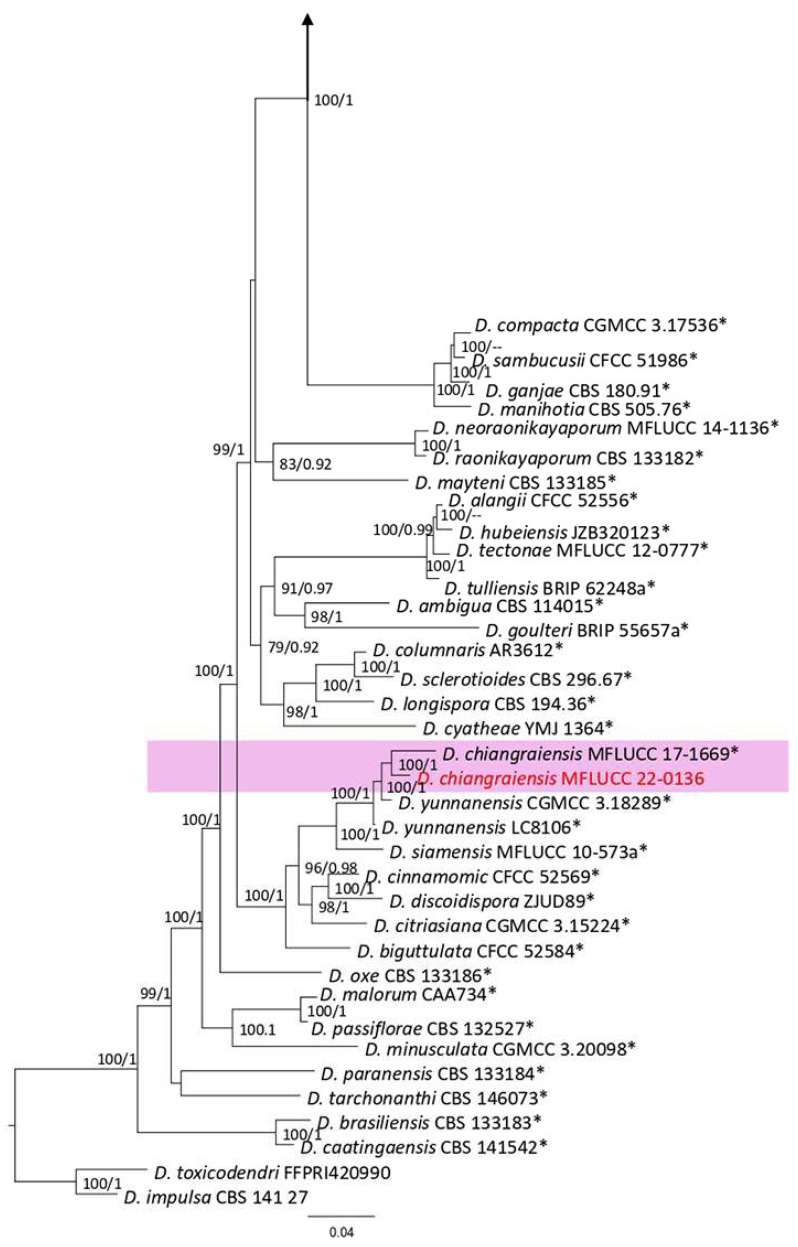
The *Diaporthe sojae* complex. Bayesian phylogenetic tree obtained from the combined ITS, *tef1*, *tub2*, *cal*, and *his3* sequence data. The tree was constructed using MrBayes 3.2.7a on CIPRES Science Gateway. The ultrafast maximum likelihood bootstrap support (%) ≥ 50 and BYPP ≥ 0.90 are shown at the nodes. The ex-type strains are marked with an asterisk. The tree is rooted in *D. toxicodendri* (FFPRI420990) and *D. impulse* (CBS 141.27).

***Diaporthe chiangraiensis*** (Senan & Hyde) Norph., 2022.

Facesoffungi number: FoF 13407.

Assocciated with *Asplenium nidus* leaf spot. Sexual morph: not observed. Asexual morph: conidiomata, pycnidium, immersed and semi-immersed, a few, aggregated and black. Sporulation, not observed (for the conidial morphology, see the description of *Chiangraiomyces bauhiniae* [38]). 

Culture characteristics: Colonies reached 75–80 mm after seven days of growth on PDA at 28 °C; felted, dull surface, circular shape, undulate edge, fluffy margin, and medium sparse density without pigmentation in the medium and fruiting body. Upper view with circles of white and primrose and the reverse with circles of straw and olivaceous buff. 

Material examined: Thailand, Chiang Rai Province, Muang District, Thasud, leaf spot on *Asplenium nidus*, 4 December 2021, Elaheh Seifollahi, dried culture (MFLU 22-0221) and living culture (MFLUCC 22-0136).

Notes: Isolated from a small blight on leaves of *Asplenium nidus*. The isolate obtained in this study (MFLUCC 22-0136) grouped with *D. chiangraiensis* with 100% ML bootstrap support and 1.0 BYPP (Figure 19). Sequence data of *cal* and *tub2* were unavailable for the ex-type strain of *D. chiangraiensis*. Comparing sequences of *D. chiangraiensis* strains (MFLUCC 22-0136 and ex-type strain MFLUCC 17-1669) revealed DNA sequence differences (including gaps) in 2% ITS (nine nucleotides) and 4.73% in *tef1* (nine nucleotides and six gaps) genomic regions. This species was first reported from dead twigs of *Bauhinia* sp. (*Fabaceae*) in Thailand [38], and it was synonymized as *D. chiangraiensis* based on the phylogenetic analysis [35]. Here, we provide a new host record for *D. chiangraiensis*.

**Figure 20:**
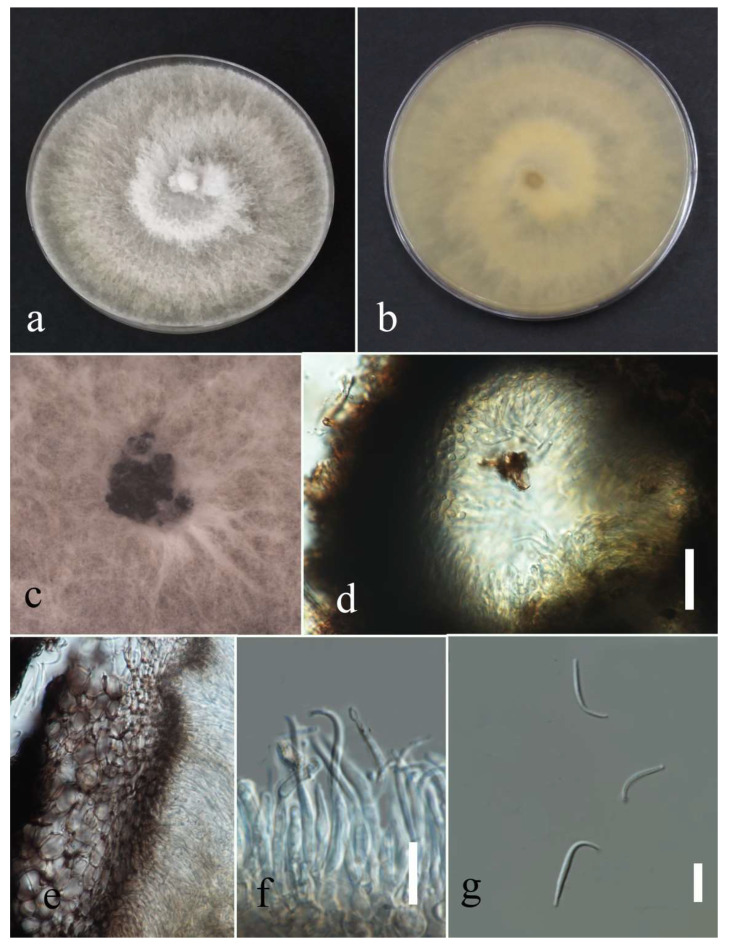
Morphology of *Diaporthe tectonendophytica* (MFLUCC 22-0140): (**a**,**b**) upper and reverse views of the colony after seven days of growth on PDA at 28 °C; (**c**) pycnidia on media; (**d**) pycnidium; (**e**) peridium; (**f**) conidiophores; (**g**) beta conidia. Scale bars (**d**) = 20 µm and (**f**,**g**) = 10 µm.

***Diaporthe tectonendophytica*** Doilom, Dissan. & K.D. Hyde, 2016; Figure 20.

Associated with *Nephrolepis cordifolia* leaf spot. Sexual morph: not observed. Asexual morph: conidiomata pycnidia, aggregated, semi-immersed, pyriform or subglobes with longish neck, black with hyaline conidial mass exuding from central ostioles after one month on PDA containing a tooth pick under florescent light at 28 °C. Conidiophores cylindrical, straight or slightly curved, hyaline, septate, unbranched, rounded at the tip and wider at the base, and 11–19 × 1–3 µm. Alfa conidia absent. Beta conidia filiform, curved at one end, hyaline, aseptate, rounded at tips, and 11–24 µm (mean = 18.93, n = 30) × 1–2 µm (mean = 1.4, n = 30). 

Culture characteristics: Colonies filled a 90 mm Petri dish after seven days growth on PDA at 28 °C; fluffy to felted, dull surface, and fluffy margin, medium sparse density without pigmentation in medium and fruiting body. Upper view white and primrose circles and the reverse view, honey in the center and primrose in other areas with honey spots. Gamma conidia, absent. 

Material examined: Thailand, Chiang Rai Province, Muang District, Thasud, leaf spot on *Nephrolepis cordifolia*, 3 December 2021, Elaheh Seifollahi, dried culture (MFLU 22-0222) living culture (MFLUCC 22-0140).

Notes: Isolated from a blight on *Nephrolepis cordifolia* leaves. The isolate obtained in this study (MFLUCC 22-0140) was grouped with *D. tectonendophytica* by 100% ML bootstrap support and 1.0 BYPP (Figure 19). Comparison of the DNA sequence data of *D. tectonendophytica* strains (MFLUCC 22-0140 and ex-type strain MFLUCC 13-0471) showed 1.46% nucleotide differences in *cal* (seven nucleotides), 0.67% in ITS (three nucleotides), 1.02% in *tef1* (two nucleotides and one gap), and 0.98% in *tub2* (two nucleotides and two gaps) genomic regions. This species was first introduced from *Tectona grandis* in Thailand [39]. Here, we provide a new host record for *D. tectonendophytica*.

***Glomerellales*** Chadef. ex Réblová, W. Gams & Seifert, *Studies in Mycology*, 68: 170 (2011).

The taxonomic structure of this order was clarified, and its name was published validly by Réblová et al. [40]. This order comprises five families: *Australiascaceae, Glomerellaceae*, *Malaysiascaceae*, *Plectosphaerellaceae*, and *Reticulascaceae* [27]. The members of this order are characterized by apostromatal and endostromatal ascomata and hyaline aseptate ascospores, which can be endophytes or plant parasites [40]. 

***Glomerellaceae*** Locq., Mycologie générale et structurale: 175 (1984).

This is a monotypic family with *Colletotrichum* asexual morph and *Glomerella* sexual morph. Members of this family are plant endophytes, pathogens, and saprobes [27].

***Colletotrichum*** Corda, Deutschlands Flora, Abt. III. Die Pilze Deutschlands. 3 (12): 41; (1831).

This genus comprises endophytes, saprobes, plant, insect, and human pathogens. Species of *Colletotrichum* are distributed in 16 species complex and 15 singleton species based on morphological and phylogenetic data [41]. In this study, seven species (*Colletotrichum polypodialium, C. fructicola, C. gigasporum, C. orchidearum, C. pandanicola, C. plurivorum*, and *C. truncatum*) belonging to *C. gloesporioides, C. gigasporum, C. orchidearum*, and *C. truncatum* complexes reported on ferns and fern-like hosts based on the morphology and combined phylogeny of the ITS, *tub2*, *act*, *gapdh*, and *chs-1* sequence data. 

**Figure 21:**
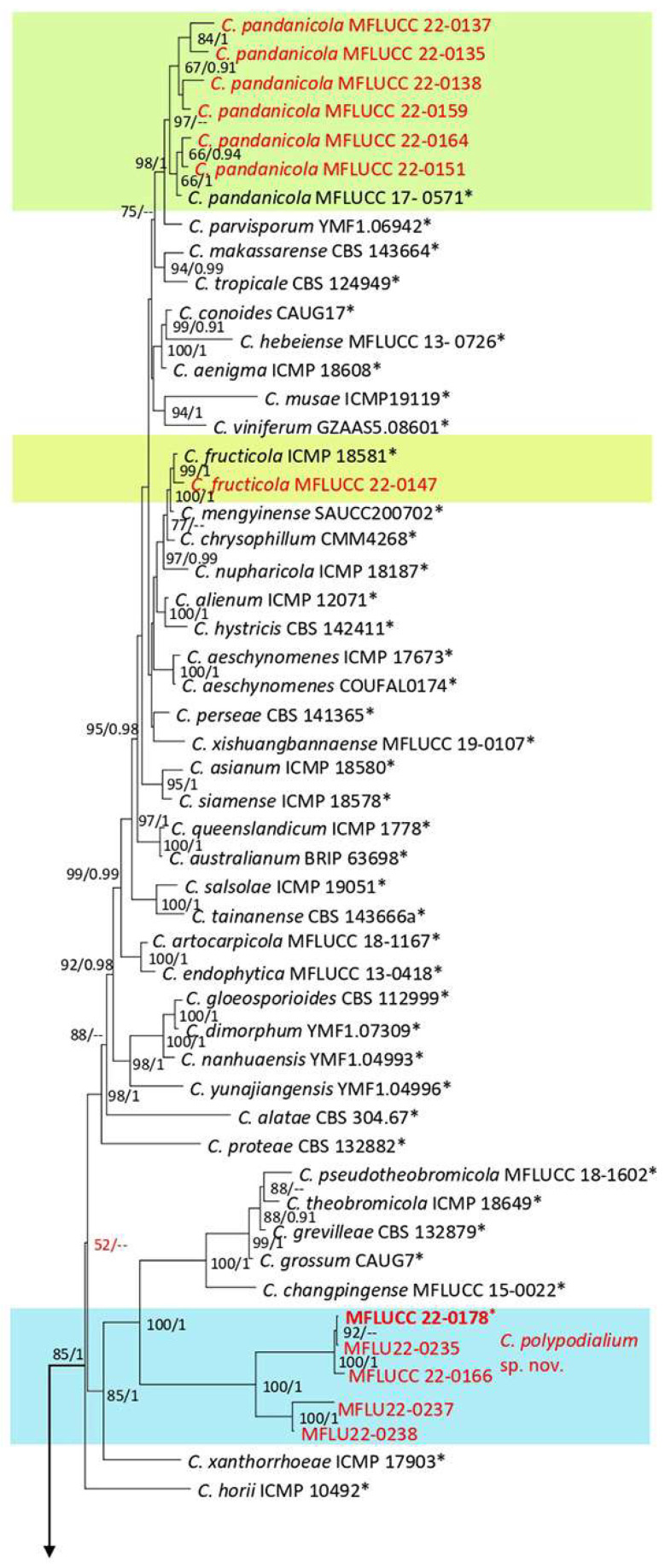

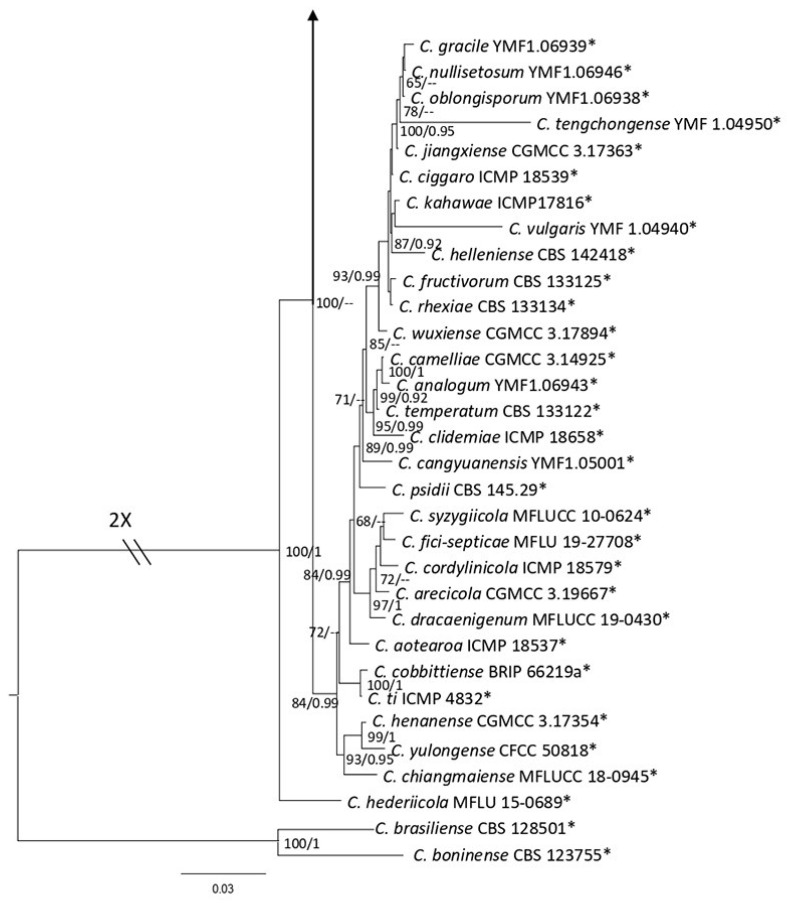
The *Colletotrichum gloesporioides* complex. The Bayesian phylogenetic tree obtained from the combined *act*, *chs-1*, *gapdh*, ITS, and *tub2* sequence data. The ultrafast maximum likelihood bootstrap support (%) ≥ 50 and BYPP ≥ 0.90 are shown at the nodes. The ex-type strains are marked with an asterisk. The tree is rooted in *C. braseiliense* (CBS 128501) and *C. boninense* (CBS 123755).

***Colletotrichum fructicola*** Prihast., L. Cai & K.D. Hyde, 2009.

**Figure 22:**
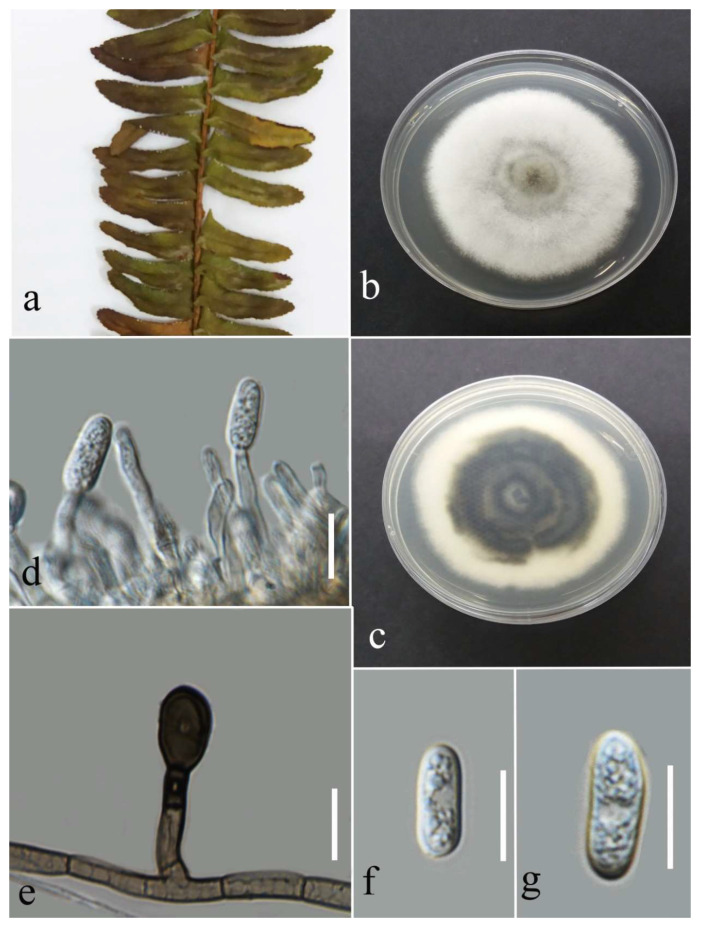
Morphology of *Colletotrichum fructicola* (MFLUCC 22-0147) obtained from *Nephrolepis cordifolia*: (**a**) blight on the leaf margin in the host; (**b**,**c**) upper and reverse views of the colony after seven days of growth on PDA; (**d**) conidiogenous cell; (**e**) appressorium; (**f**,**g**) conidia. Scale bars = 10 µm.

Facesoffungi number: FoF 06767; Figure 22.

Associated with *Nephrolepis cordifolia* leaf spot. Sexual morph: not observed. Asexual morph: on PDA Conidiomata acervulus, semi-immersed, with orange conidial mass. Setae absent. Conidiophores reduced to conidiogenous cells. Conidiogenous cells cylindrical, hyaline, and entroblastic. Conidia cylindrical, hyaline, rounded apices, and 11–15 µm (mean = 13.2, n = 30) × 4–6 (mean = 5.3, n = 30). Appressoria clavate, ovoid and slightly irregular or regular shape, brown to dark-brown, and s6–14 µm (mean = 9. 5, n = 20) × 4–11 µm (mean = 7.4, n = 20).

Culture characteristics: Colony reached 59–68 mm after seven days of growth on PDA at 28 °C; cottony, circular shape, dull surface, entire edge, well-defined margin, and medium density, without conidial mass and pigmentation. Upper view pale olivaceous gray in the center, smoke gray in the middle, and white margin, and the reverse with circles of dull green, greenish gray, and primrose margin.

Material examined: Thailand, Chiang Rai Province, Muang District, Thasud, leaf spot on *Nephrolepis cordifolia*, 3 December 2021, Elaheh Seifollahi, dried culture (MFLU 22-0223) and living culture (MFLUCC 22-0147).

Notes: Isolated from a blight on the leaf margin of *Nephrolepis cordifolia*. The isolate obtained in this study (MFLUCC 22-0147) clustered with *C. fructicola* by 99% ML bootstrap support and 1.00 BYPP (Figure 21). The DNA sequence data of *C. fructicola* strains (MFLUCC 22-0147 and ex-type strain ICMP 18581) differed 1.05% in *act* (one nucleotide and one gap) and 0.41% in *tub2* (two nucleotides), while the sequences of *chs-1*, *gapdh*, and ITS were identical. This species was first reported from *Coffea arabica* in Thailand [42]. It was also isolated from *Capsicum annuum*, *Carica papaya*, *Cymbopogon citratus*, *Dendrobium* sp., *Dimocarpus longan*, *Freycinetia* sp., *Freycinetia* sp., *Pandanus* sp., and *Pennisetum purpureum* in Thailand [43]. Here, we provide a new host record for *C. fructicola*. 

***Colletotrichum pandanicola*** Tibpromma & K.D. Hyde.

**Figure 23:**
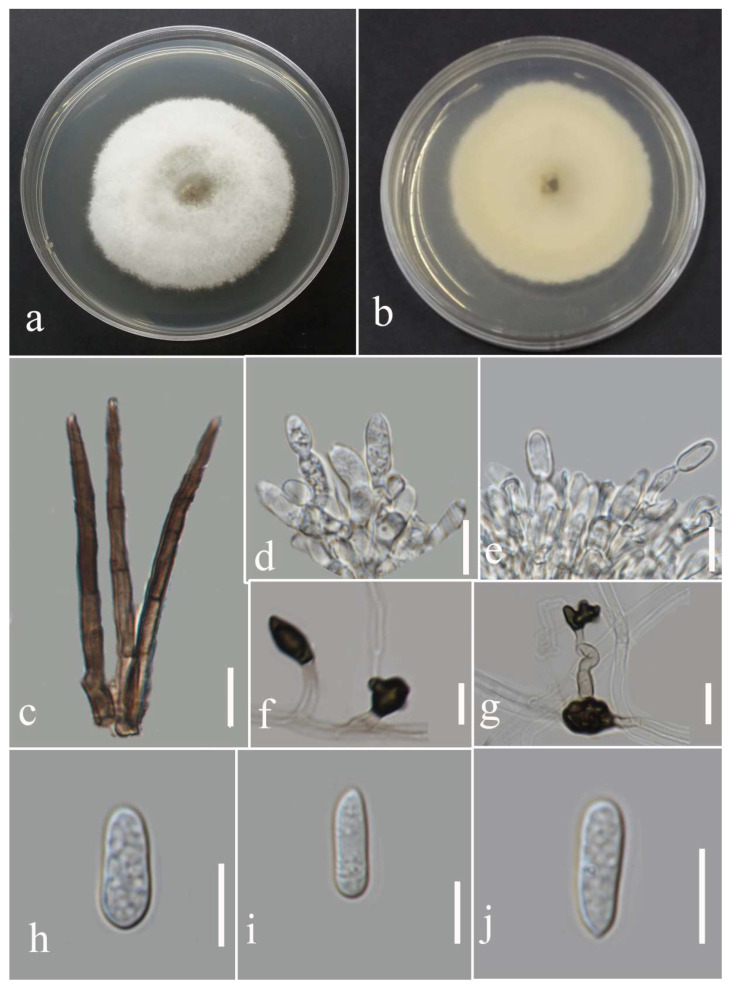
Morphology of *Colletotrichum pandanicola* (MFLUCC 22-0137) obtained from *Nephrolepis cordifolia*: (**a**,**b**) upper and reverse views of the colony after seven days of growth on PDA at 28 °C; (**c**) setae; (**d**,**e**) conidiogenous cells; (**f**,**g**) appresoria; (**h**–**j**) conidia. Scales bars = 10 µm.

Facesoffungi number: FoF 04534; Figure 23.

Associated with *Nephrolepis cordifolia* leaf spot. Sexual morph: not observed on PDA. Asexual morph: conidiomata acervulus, semi-immersed, scattered with orange conidial mass. Setae formed on PDA after two months, brown, 3–4 cell, and 255–358 × 10–18 µm. Conidiohpores reduced to conidiogenous cells. Conidiogenous cells cylindrical or clavate, hyaline, enteroblastic, and 4–9 µm (mean = 6. 8, n = 20) × 2–5 µm (mean = 3.4, n = 20). Conidia cylindrical, hyaline with rounded apices and 10–15 µm (mean = 12.6, n = 30) × 2–6 µm (mean = 4.1, n = 30). Appressoria formed in slide culture on SNA medium after one week with diverse shapes, lobated, knobbed brown to dark-brown, and 6–14 µm (mean = 8.9, n = 20) × 2–8 µm (mean = 5.7, n = 20).

Culture characteristics: Colonies reached 56–71 mm diameter after seven days of growth on PDA at 28 °C; fluffy, dull surface, entire edge, fluffy margin, and medium density, without pigmentation in media and conidial mass. Upper view smoke gray in the center and white in other parts and the reverse primrose.

Material examined: Thailand, Chiang Rai Province, Muang District, Huai Sak, leaf spot on *Nephrolepis cordifolia* (*Nephrolepidaceae*), 17 December 2021, Elaheh Seifollahi, dried culture (MFLU 22-0224) and living (MFLUCC 22-0137); ibid., Thasud, leaf spot on *Nephrolepis cordifolia*, 4 December 2021, Elaheh Seifollahi, dried culture (MFLU 22-0227) and living culture (MFLUCC 22-0159); ibid., leaf spot on *Pteris ensiformis* (*Pteridaceae*), 4 December 2021, Elaheh Seifollahi, dried culture (MFLU 22-0225) and living culture (MFLUCC 22-0135); ibid., leaf spot on *Cyclosorus* sp., 4 December 2021, Elaheh Seifollahi, dried culture (MFLU 22-0226) and living culture (MFLUCC 22-0138); ibid., leaf spot on *Cyclosorus* sp. (*Thelypteridaceae*), 4 December 2021, Elaheh Seifollahi, dried culture (MFLU 22-0228) and living culture (MFLUCC 22-0164); ibid., leaf spot on *Cyclosorus* sp., 17 December 2021, Elaheh Seifollahi, dried culture (MFLU 22-0229) and living culture (MFLUCC 22-0151).

Notes: Isolated from a dark necrotic margin (*Cyclosorus* sp.), brown spot (*Pteris ensiformis*), and necrotic lesions (*Nephrolepis cordifolia*). In this study, the isolates obtained from fern species (MFLUCC 22-0137, MFLUCC 22-0135, MFLUCC 22-0138, MFLUCC 22-0159, T22-0231, and MFLUCC 22-0151) clustered in the same clade with *C. pandanicola*, with 97–98% ML bootstrap support (Figure 21). Pairwise nucleotide comparison of the ex-type strain (MFLUCC 17-0571) with strains in this study are shown in Table 1. This species was first reported from *Pandanus* sp. in Thailand as an endophytic fungus [44]. This is the first report of *C. pandanicola* from *Nephrolepis cordifolia*, *Pteris ensiformis*, and *Cyclosorus*.

***Colletotrichum polypodialium*** Seifollahi, Jayaward., K.D. Hyde. 

**Figure 24:**
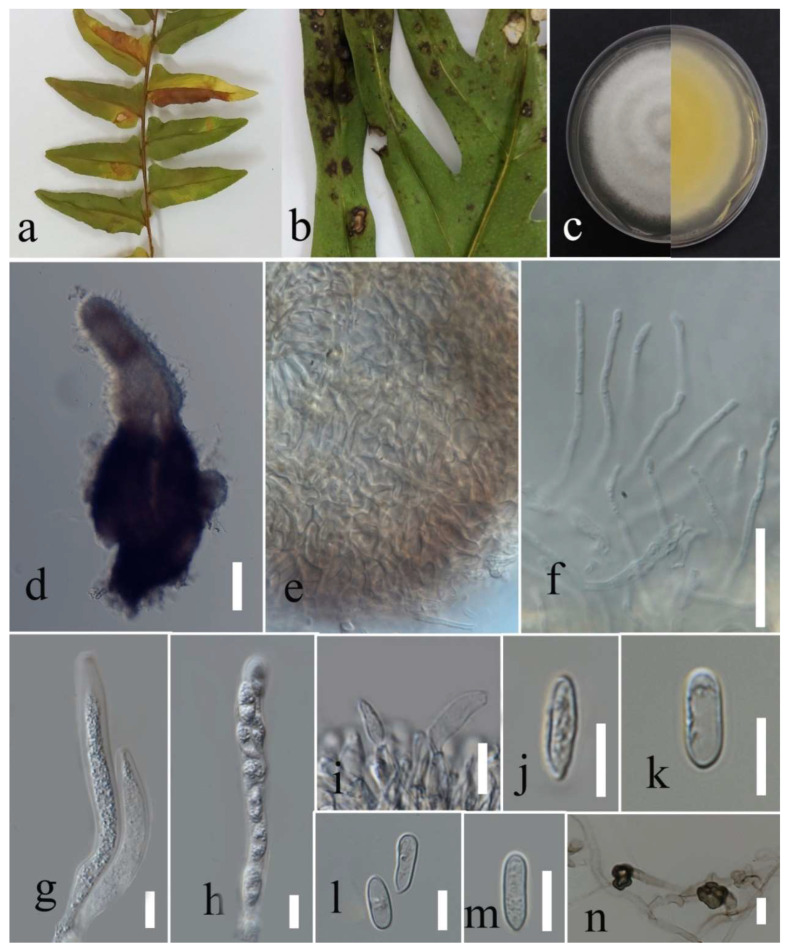
Morphology of *Colletotrichum polypodialium* (ex-type: MFLUCC 22-0178 for asexual morph and culture; MFLU 22-0235 for sexual morph): (**a**,**b**) symptoms on *Nephrolepis* sp. and *Phymatosorus* sp.; (**c**) upper and reverse views of culture after seven days of growth on PDA at 28 °C; (**d**) perithecium; (**e**) peridium; (**f**) paraphyses; (**g**) immature asci; (**h**) mature ascus; (**i**) conidiogenous cells; (**j**) ascospore; (**k**–**m**) conidia; (**n**) appressoria. Scale bars (**d**) = 100 µm, (**f**) = 20 µm, and (**g**–**n**) = 10 µm.

Index Fungorum number: IF 559325; Facesoffungi number: FoF 13408; Figure 24.

Etymology: The species epithet refers to the plant order (*Polypodiales*) from which it was isolated.

Saprobe and associated with *Nephrolepis* sp. leaf spots and other leaves necrotic symptoms. Sexual morph: ascomata, perithecia aggregated, semi-immersed, ostiolate, pyriform, medium to dark brown, 360–600 µm high, and 125–240 µm wide. Asci cylindrical to clavate, hyaline, unitunicate, eight ascospores, uniseriate arrangement, truncate at tips, and 67–99 × 7–11 µm. Paraphyses septate. Ascospores fusiform, hyaline, aseptate, rounded at tips, and 13–18 µm (mean = 15.0, n = 30) × 3–7 µm (mean = 5.4, n = 30). Asexual morph: conidiomata acervulus with orange conidial mass. Setae absent. Conidiophores reduced to conidiogenous cells. Conidiogenous cells cylindrical, hyaline to pale brown, and 7–13 × 2–5 µm. Conidia cylindrical, hyaline, aseptate, round at tips, and 11–16 µm (mean = 13.6, n = 30) × 3–8 µm (mean = 4.8, n = 30). Appressoria lobate, pale to medium brown, irregular outline, singular, and 5–13 µm (mean = 8.8, n = 20) × 5–15 (mean = 9.3, n = 20).

*Culture characteristics*: Colonies reached 78–82 mm after seven days of growth on PDA at 28 °C; fluffy, circular, dull surface, entire to lobate edge, fluffy margin, and medium density without conidial mass and pigmentation. Upper view white and the reverse straw. 

Material examined: Thailand, Chiang Rai Province, Muang District, Thasud, leaf spot on *Nephrolepis* sp., 4 December 2021, Elaheh Seifollahi, dried culture (MFLU 22-0234, **holotype**); ex-type living culture (MFLUCC 22-0178) ibid., dried culture (MFLU 22-0235); ibid., leaf spot on *Phymatosorus* sp. (*Polypodiaceae*), 4 December 2021, Elaheh Seifollahi, dried culture (MFLU 22-0236); living culture (MFLUCC 22-0166); ibid., dried culture (MFLU 22-0238); ibid., dead leaves of *Phymatosorus* sp., 4 December 2021, Elaheh Seifollahi, dried culture (MFLU 22-0237).

**Figure 25:**
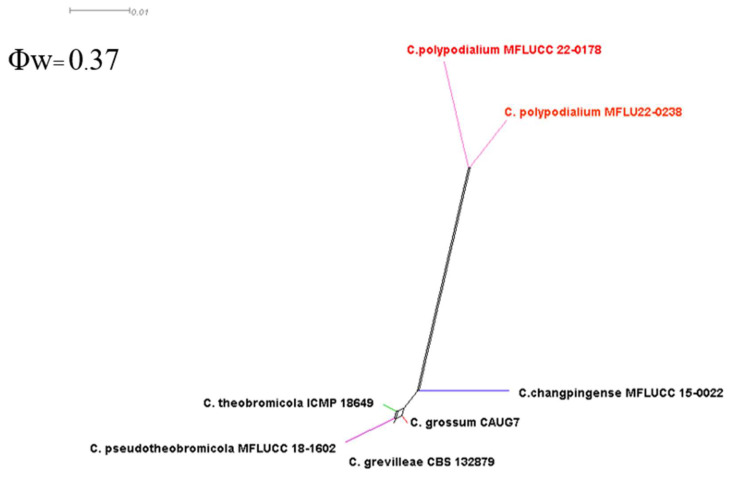
Pairwise homoplasy index (PHI) test of closely related species using LogDet transformation. The PHI test results (Φ_w_) < 0.05 indicate significant recombination within the dataset.

Notes: Associated with leaf spots, dark necrotic margin or anthracnosis lesion on *Nephrelepis* sp., and brown spots and dark-brown necrotic margin lesion on *Phymatosorus* sp. Perithecium was formed in the isolates MFLU 22-0235, MFLUCC 22-0178, and MFLUCC 22-0166 on PDA after two months of disruption with toothpicks, while it was not observed in the isolates MFLU 22-0238 and MFLU 22-0237. After six months, we could not maintain a living culture for the isolates MFLU 22-0235, MFLU 22-0237, and MFLU 22-0238 on PDA, 15% glycerol and distilled water at 16, −21, and 25 °C, respectively. Our isolates (MFLU 22-0235, MFLU 22-0234, MFLU 22-0236, MFLU 22-0238, MFLUCC 22-0166, and MFLU 22-0237) clustered in a separate group with 100% ML ultrafast bootstrap supports and 1.00 BYPP (Figure 21), closely related to *C. pseudotheobromicola*, *C. theobromicola*, *C. grevilleae*, *C. grossum*, and *C. changpingense* (Figure 21). Pairwise comparison of the ex-type strain sequences of *C. polypodialium* and *C. changpingense* revealed 6.88% nucleotide differences in *act* (15 nucleotides), 7.79% in *chs-1* (18 nucleotides), 17.22% in *gapdh* (35 nucleotides and one gap), 1.78% in ITS (8 nucleotides and one gap), and 8.11% in *tub2* (36 nucleotides and one gap). The PHI test did not show significant recombination between the *C. polypodialium* and *C. changpingense* isolates (Φ_w_ = 0.37) (Figure 25). Based on the recommendations of Chethana et al. [45] and Jayawardena et al. [46,47], we introduce *C. polypodialium* to accommodate the newly obtained isolates.

**Figure 26:**
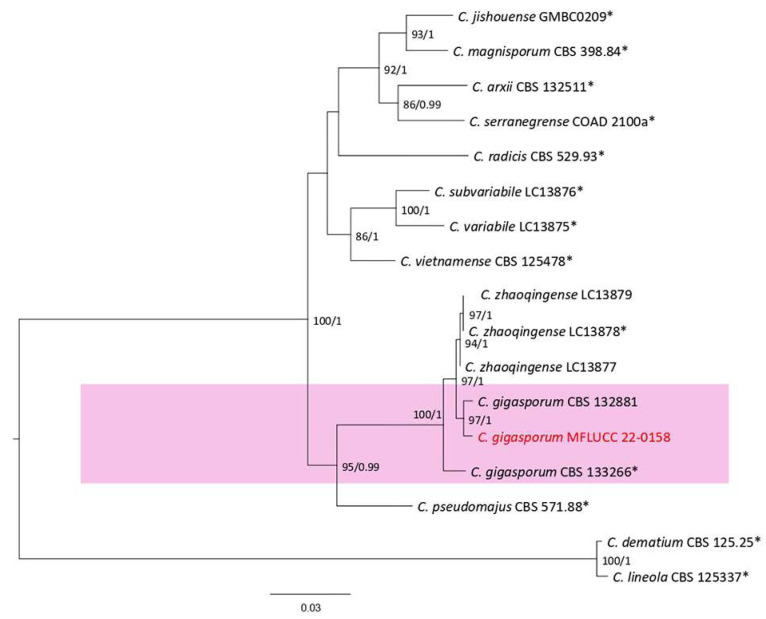
The *Colletotrichum gigasporum* complex maximum likelihood phylogenetic tree obtained from the combined *act*, *chs-1*, *gapdh*, ITS, and *tub2* sequence data. The ultrafast maximum likelihood bootstrap support (%) ≥ 50 and BYPP ≥ 0.90 are shown at the nodes. Th ex-type strains are marked with an asterisk. The tree is rooted in *C. dematium* (CBS 125.25) and *C. lineola* (CBS 125337).

**Figure 27:**
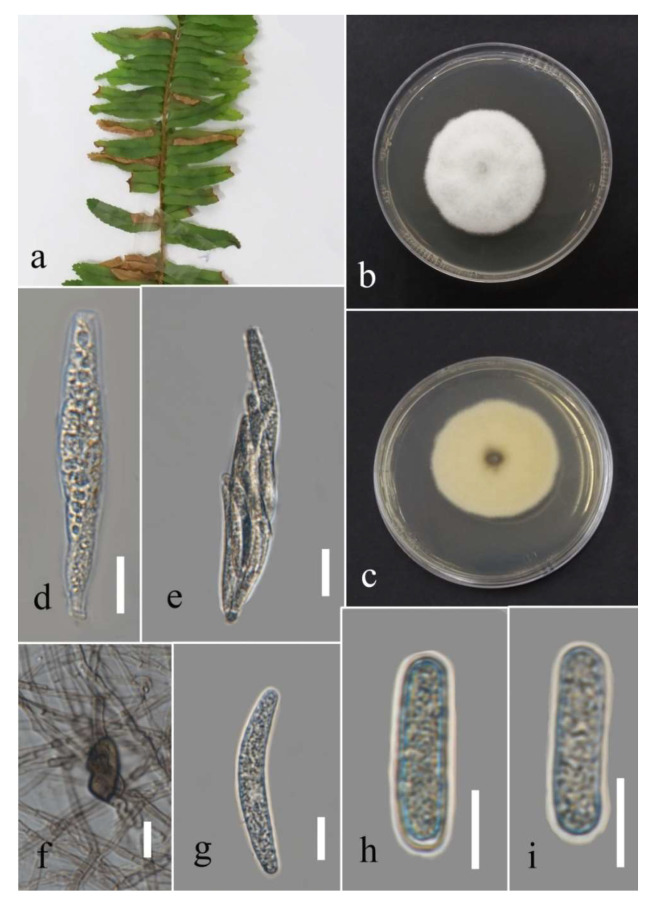
Morphology of *Colletotrichum gigasporum* (MFLUCC 22-0158): (**a**) leaf blight on *Nephrolepis cordifolia*; (**b**,**c**) upper and reverse views of the culture after seven days of growth on PDA at 28 °C; (**d**) immature ascus; (**e**) mature ascus; (**f**) appressorium; (**g**) ascospore; (**h**,**i**) Conidia. Scale bars (**d**,**e**) = 20 µm and (**f**–**i**) = 10 µm.

***Colletotrichum gigasporum*** Rakotonir. & Munaut, 2013; Figure 27.

Associated with *Nephrolepis cordifolia* leaf blight. Sexual morph formed on PDA containing tooth pick after two months. Ascomata, perithecia solitary or aggregated, obpyriform to subglobose, and covered with mycelia. Asci cylindrical to clavate, hyaline, unitunicate, eight ascospores, truncate at apex, 85–124 × 16–22 µm. Ascospores falcate, hyaline, aseptate, rounded tips, and 34–60 µm (mean = 49.1, n = 30) × 4–10 µm (mean = 7.5, n = 30). 

Asexual morph: Conidiomata condiophores and conidiogenous cells not observed. Conidia cylindrical, hyaline, aseptate, and rounded at both tips, and 21–32 µm (mean = 26.6, n = 30) × 6–10 µm (mean = 7.55, n = 30). Appresorria clavate, pale to medium brown, regular or irregular outline with a germ pore, and 17–20 × 8–12 µm. 

Culture characteristics: Colonies reached 47–52 mm after seven days of growth on PDA at 28 °C; cottony, circular shape, dull surface, entire edge, and well-defined margin with medium density and without pigmentation in media and conidial mass. Upper view white and the reverse dull green in the center and primrose in other parts. 

Material examined: Thailand, Chiang Rai Province, Muang District, Thasud, leaf spot on *Nephrolepis cordifolia*, 4 December 2021, Elaheh Seifollahi, dried culture (MFLU 22-0239) and living culture (MFLUCC 22-0158).

Notes: Strain MFLUCC 22-0158 was isolated from a necrotic spot with a brown margin on *Nephrolepis cordifolia* leaves. The isolate obtained in this study (MFLUCC 22-0158) clustered with *C. gigasporum* in the same clade by 97% ML bootstrap support and 1.0 BYPP (Figure 26). The DNA sequence data of *C. gigasporum* strains (MFLUCC 22-0158 and CBS 133266) differed 2.13% in ITS (seven nucleotides), 1.94% in *chs-1* (one gap and four nucleotides), 2.83% in *gapdh* (seven nucleotides), and 1.44% in *tub2* (one gap and six nucleotides). The sequence of *act* for the type strain is not available. *Colletotrichum gigasporum* was first introduced from *Centella asiatica*, *Stylosanthes guianensis*, and *Coffea arabica* from Madagascar, Mexico, and Colombia [48]. It was also reported on *Acacia auriculiformis*, *Alocasia* sp. and *Hibiscus rosa-sinensis* in Thailand [43,47,49]. Here, we first report it from *N. cordifolia*. 

**Figure 28:**
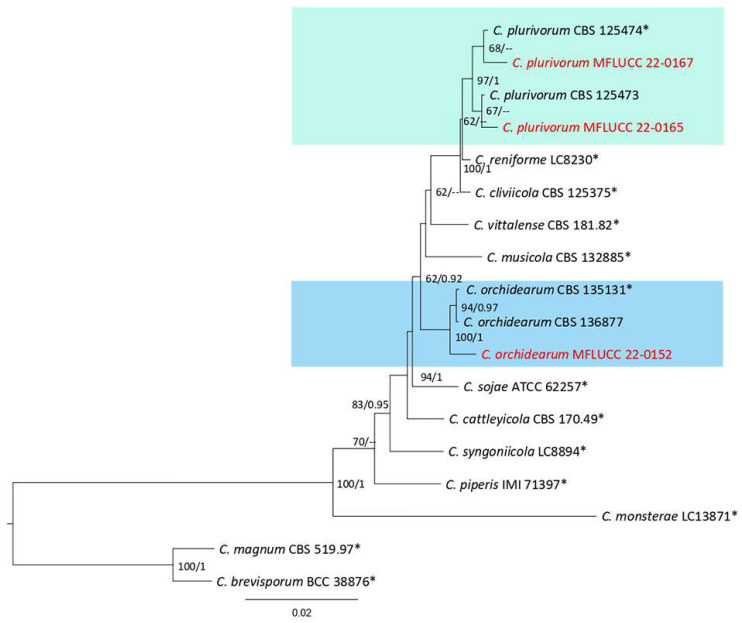
The *Colletotrichum orchidearum* complex. Bayesian phylogenetic tree obtained from combined *act*, *chs-1*, *gapdh*, ITS, and *tub2* sequence data. The tree was constructed using MrBayes 3.2.7a on CIPRES Science Gateway. The ultrafast maximum likelihood bootstrap support (%) ≥ 50 and BYPP ≥ 0.90 are shown at the nodes. The ex-type strains are marked with an asterisk. The tree is rooted in *C. magnum* (CBS 519.97) and *C. boninense* (BCC 38876).

***Colletotrichum orchidearum*** Allesch., Rabenhorst’s Kryptogamen-Flora, Pilze, 1902. 

**Figure 29:**
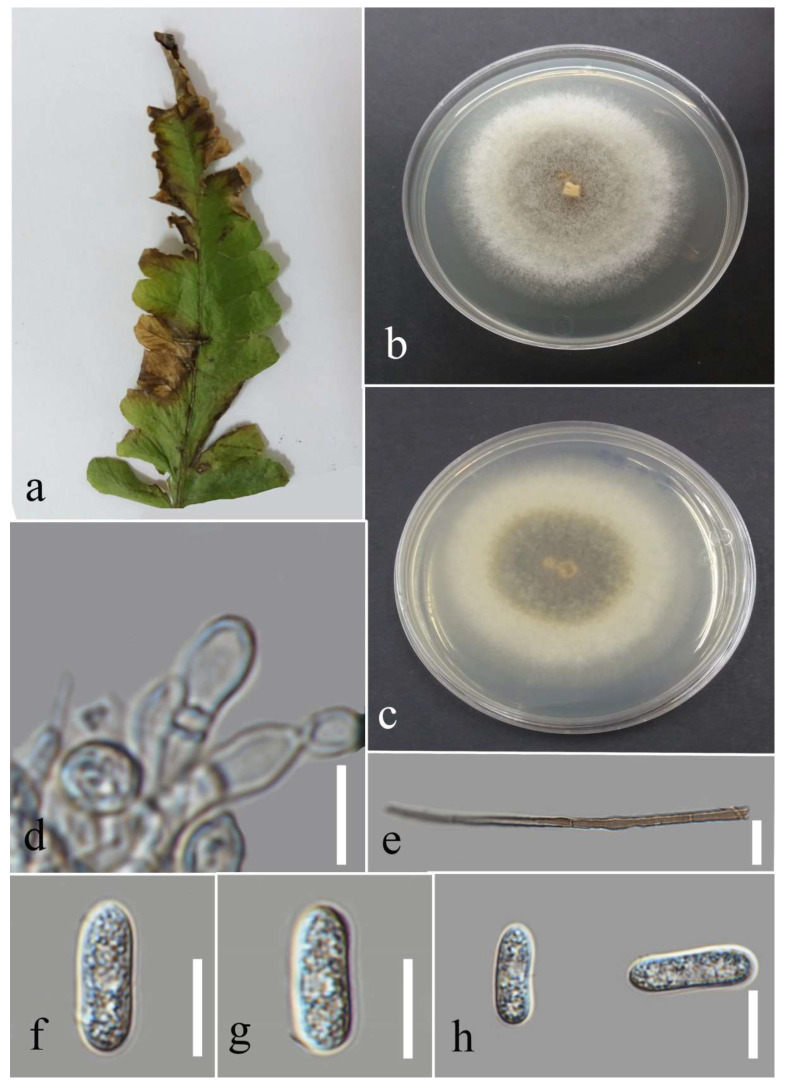
Morphology of *Colletotrichum orchidearum* (MFLUCC 22-0152): (**a**) necrotic lesion on *Cyclosorus* sp.; (**b**–**c**) upper and reverse views of the colony after seven days of growth on PDA at 28 °C; (**d**) conidiogenous cells; (**e**) satae; (**f**–**h**) conidia. Scale bars (**e**) = 20 µm and (**d**,**f**–**h**) = 10 µm.

Facesoffungi number: FoF 13409; Figure 29.

Associated with *Cyclosorus* sp. necrotic lesion. Sexual morph: not observed. Asexual morph: conidiomata acervulus. Setae present, medium brown to dark-brown, 3–4-septate, and tip acute to round. Conidiophores reduced to conidiogenous cells. Conidiogenous cells cylindrical to doliiform, hyaline to pale brown, and 2–11 × 3–5 µm. Conidia cylindrical, hyaline, aseptate, rounded apices, and 10–18 (mean = 14.3, n = 30) × 3–7 µm (mean = 5.4, n = 30). Appressoria absent.

Culture characteristics: Colonies reached 65–72 mm after seven days of growth on PDA at 28 °C; fluffy, circular, entire edge, fluffy margin, dull surface, and medium density, without pigmentation in media and conidial mass. Upper view pale olivaceous with white margin and the reverse gray olivaceous to dull green with primrose margin. 

Material examined: Thailand, Chiang Rai Province, Muang District, Huai sak, leaf spot on *Cyclosorus* sp., 17 December 2021, Elaheh Seifollahi, dried culture (MFLU 22-0240) and living culture (MFLUCC 22-0152).

Notes: The isolate obtained in this study (MFLUCC 22-0152) clustered with *C. orchidearum* in the same clade by 100% ML bootstrap support and 1.0 BYPP (Figure 28). The DNA sequence data of *C. orchidearum* strains (ex-type strain CBS 135131 and T22-0385) showed 1.17% nucleotide differences in *chs-1* (three nucleotides), 0.48% in *gapdh* (one gap), and 1.03% in *tub2* (three nucleotides and two gaps), while the sequences of ITS and *act* were identical. *Colletotrichum orchidearum* was revised using obtained isolates from *Eria javanica*, *Epipremnum aureum*, *Dendrobium nobile*, and *Hymenocallis* sp. in Germany, Iran, Netherlands, and Thailand [50]. Here, we provided a new host record for this species. 

**Figure 30:**
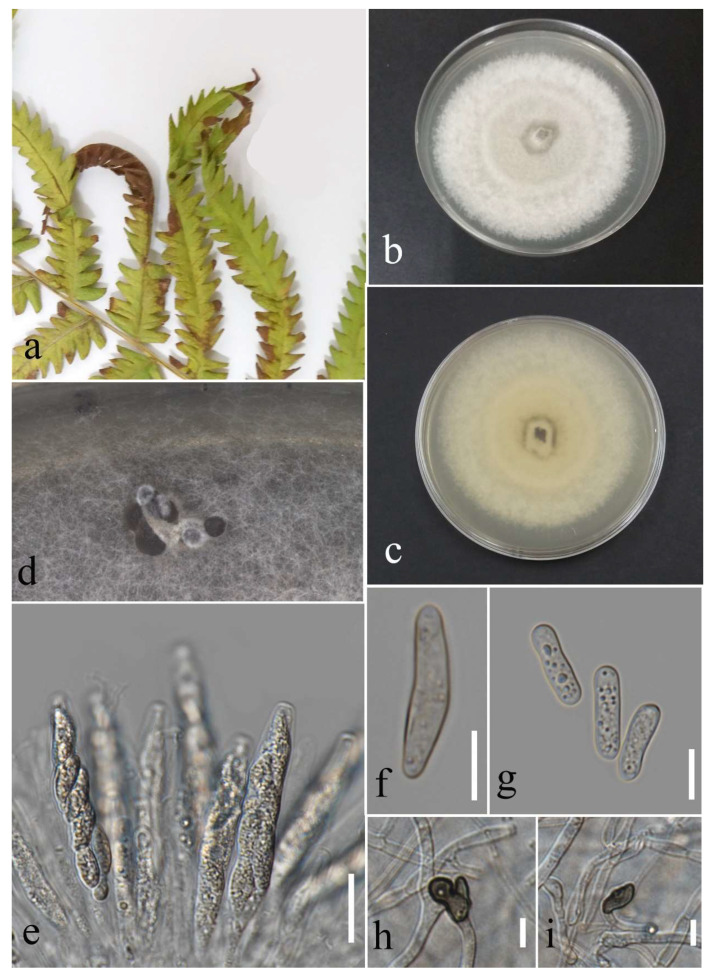
Morphology of *Colletotrichum plurivorum* based (MFLUCC 22-0167): (**a**) symptoms *Cyclosorus* sp.; (**b**,**c**) upper and reverse views of the colony after seven days of growth on PDA at 28 °C; (**d**) ascomata on PDA; (**e**) asci; (**f**) ascospore; (**g**) conidia; (**h**–**i**) appressoria. Scales bars (**e**) = 20 µm (**f**–**i**) = 10 µm.

***Colletotrichum plurivorum*** Damm, Alizadeh & Toy. Sato, 2018; Figure 30.

Associated with *Cyclosorus* sp. leaf necrotic lesions. Sexual morph: formed on PDA containing tooth pick at room temperature after two months, ascomata, perithecia aggregated, semi-immersed, globes, and covered with sparse white aerial mycelia. Asci cylindrical to clavate, hyaline, unitunicate, eight ascospores, and 67–86 × 10–12 µm. Ascospores allantoid to fusiform, initially hyaline, aseptate, rounded tips, and 12–31 µm (mean = 18.2, n = 30) × 4–8 µm (mean = 5.5, n = 30). 

Asexual morph: conidiomata acervulus. Setae absent. conidiophores reduced to conidiogenous cells. Conidiogenous cells cylindrical and hyaline. Conidia cylindrical, hyaline, aseptate with rounded apices, and 10–21 µm (mean = 16.3, n = 30) × 3–6 µ (mean = 4.7, n = 30). Appressoria diverse shape, bullet-shaped to lobate, brown, regular or irregular margin, and 7–13 µm (mean = 9.5, n = 20) × 4–8 (mean = 6.2, n = 20). 

Culture characteristics: Colonies filled the 90 mm Petri dish after seven days of growth on PDA at 28 °C; fluffy to cotton, circular shape, dull surface, entire edge, with fluffy margin and medium density, without pigmentation in media and conidial mass. Upper view circles of smoke gray and white and the reverse circles of greenish olivaceous and primrose with greenish olivaceous dots. 

Material examined: Thailand, Chiang Rai Province, Muang District, Thasud, leaf necrotic lesions on *Cyclosorus* sp., 17 December 2021, Elaheh Seifollahi, dried culture (MFLU 22-0242) and living culture (MFLUCC 22-0165); ibid., leaf spot on *Cyclosorus* sp., 17 December 2021, Elaheh Seifollahi, dried culture (MFLU 22-0241) and living culture (MFLUCC 22-0167). 

Notes: Strains MFLUCC 22-0165 and MFLUCC 22-0167 were isolated from leaf necrotic lesions with a dark-brown margin and anthracnose, respectively, on *Cyclosorus* species. Conidiomata, formed in isolate MFLUCC 22-0165, while it was not observed for isolate MFLUCC 22-0167. Additionally, conidiophores and conidiogenous cells were not observed in isolate MFLUCC 22-0167. The isolates obtained in this study clustered with *C. plurivorum* in the same clade by 97% ML bootstrap support and 1.0 BYPP (Figure 28). Pairwise comparison sequences of the ex-type strain and strain MFLUCC 22-0167 revealed 0.39% nucleotide differences in *chs-1* (one nucleotide), 0.98% in *gapdh* (one nucleotide and one gap), 0.39% in ITS (two gaps), and 0.62% in *tub2* (two nucleotides and one gap). The pairwise comparison results for strain MFLUCC 22-0165 showed 0.86% nucleotide differences in *act* (two nucleotides), 0.39% in *chs-1* (one nucleotide), 0.97% in *gapdh* (one nucleotide and one gap), 0.19% in ITS (one nucleotide), and 1.11% in *tub2* (four nucleotides and two gaps). *Colletotrichum plurivorum* was introduced causing anthracnose from *Phaseolus lunatus*, *Gossypium* sp., *Spathiphyllum wallisii*, *Phaseolus vulgaris*, and *Coffea* sp. in Benin, Brazil, Iran, and Vietnam, [50]. Later, it was reported on *Capsicum annuum* and *Capsicum* sp. in Thailand [51]. This study introduces *Cyclosorus* sp. as a new host of *C. plurivorum*. 

**Figure 31:**
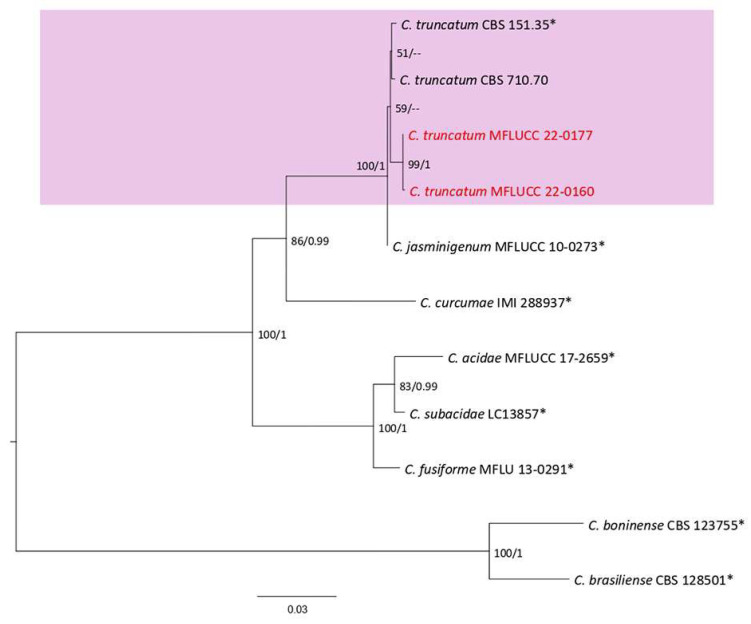
The *Colletotrichum truncatum* complex maximum likelihood phylogenetic tree obtained from combined *act*, *chs-1*, *gapdh*, ITS, and *tub2* sequence data. The ultrafast maximum likelihood bootstrap support (%) ≥ 50 and BYPP ≥ 0.90 are shown at the nodes. The ex-type strains are marked with an asterisk. The tree is rooted in *C. boninense* (CBS 123755) and *C. lineola* (CBS 128501).

**Figure 32:**
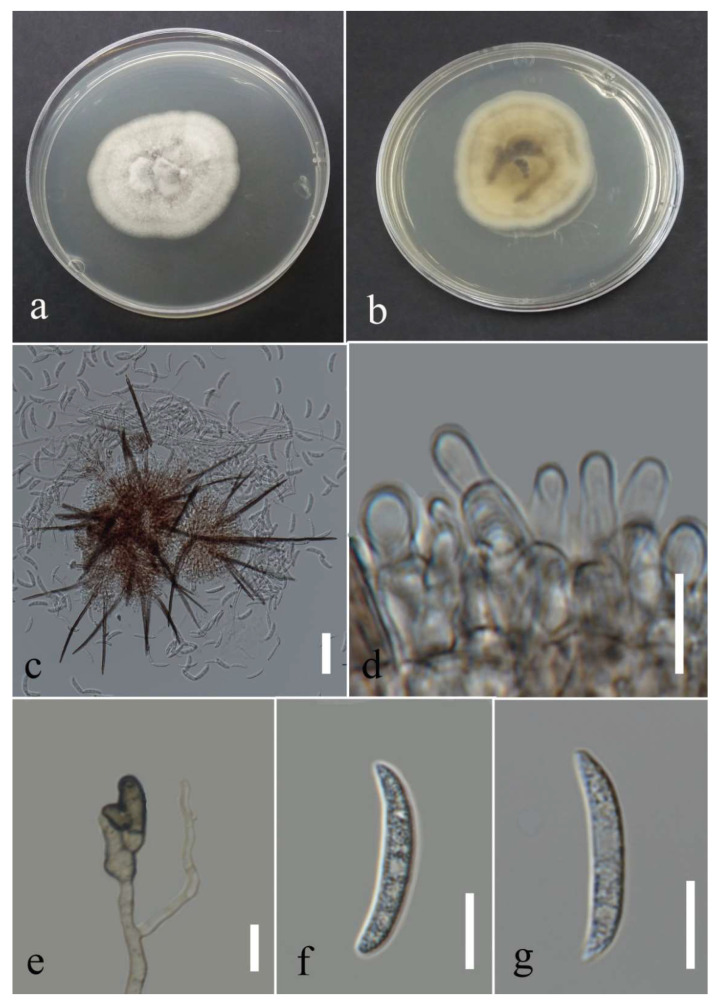
Morphology of *Colletotrichum truncatum* (MFLUCC 22-0177) obtained from *Cyclosorus* sp.: (**a**,**b**) upper and reverse views of the colony after seven days growth on PDA at 28 °C; (**c**) acervulus and setae; (**d**) conidiogenous cells; (**e**) appressoria; (**f**,**g**) fulcate conidia. Scale bars (**c**) = 50 µm and (**d**–**g**) = 10 µm.

***Colletotrichum truncatum*** (Schwein.) Andrus & W.D. Moore, 1935; Figure 32.

Associated with *Cyclosorus* sp. leaf spot. Sexual morph: not observed. Asexual morph: conidiomata acervulus. Setae medium to dark brown, 3 septate, round to slightly acute tip, and conical to cylindrical base. Conidiophores reduced to conidiogenous cells. Conidiogenous cells cylindrical, hyaline to pale brown, entroblastic, and 4–10 × 3–5 µm. Conidia falcate, hyaline, aseptate, and 16–27 µm (mean = 22.1, n = 30) × 2–5 µm (mean = 4.1, n = 30). Appressoria diverse shaped, lobed or bullet-shaped, pale to medium brown, entire edge to lobed, in a group or solitary, and 8–16 × 4–8 µm. 

Culture characteristics: Colonies reached 40–47 mm after seven days of growth on PDA at 28 °C; fluffy, circular, dull surface, entire edge, well-defined margin, and medium density, without pigmentation in media and conidial mass. Upper view white with smoke gray circles and spots and the reverse olivaceous in the center with gray olivaceous and primrose circles. 

Material examined: Thailand, Chiang Rai Province, Muang District, Thasud, leaf spot on *Cyclosorus* sp., 4 December 2021, Elaheh Seifollahi, dried culture (MFLU 22-0243), living culture MFLUCC 22-0177; Thailand, Thasud, Muang District, Chiang Rai Province, leaf spot on *Cyclosorus* sp., 4 December 2021, Elaheh Seifollahi, dried culture (MFLU 22-0244) and living culture (MFLUCC 22-0160).

Notes: Strains MFLUCC 22-0177 and MFLUCC 22-0160 were isolated from small brown spots and necrotic spots with brown margins, respectively, on the leaves of two *Cylosorus* sp. The isolates from this study (MFLUCC 22-0177 and MFLUCC 22-0160) clustered with *C. truncatum* in the same clade by 59% ML bootstrap support (Figure 31). A pairwise comparison sequence data of the ex-type strain and strain MFLUCC 22-0177 revealed 1.76% nucleotide differences in *act* (two nucleotides and two gaps), 1.72% in *chs-1* (four nucleotides), 0.89% in *gapdh* (two nucleotides), and 0.19% in ITS (one gap), while the *tub2* sequences were identical. The pairwise comparison results for strain MFLUCC 22-0160 showed 1.76% nucleotide differences in *act* (two nucleotides and two gaps), 1.72% in *chs-1* (four nucleotides), 1.34% in *gapdh* (three nucleotides), and 0.19% in ITS (one gap), while the *tub2* sequences were identical. *Colletotrichum truncatum* was revised using the obtained isolate from *Phaseolus* sp., *Phaseolus lunatus*, *Capsicum frutescens*, and *Arachis hypogaea* in the USA and India [52]. It was obtained from *Capsicum annuum*, *C. frutescens*, *Capsicum* sp., *Glycine max*, *Gossypium* sp., *Hymenocallis* sp., *Manihot esculenta*, *Solanum melongena*, *Stylosanthes hamate*, and *Vigna sesquipedalis* in Thailand [43]. Here, it was isolated from *Cyclosorus* sp., providing a new host of *C. truncatum*. 

***Hypocreales*** Lindau, Die Natürlichen Pflanzenfamilien nebst ihren Gattungen und wichtigeren Arten 1. (1): 343 (1897).

Based on molecular data, this order comprised 14 families: *Bionectriaceae*, *Calcarisporiaceae*, *Clavicipitaceae*, *Cocoonihabitaceae*, *Cordycipitaceae*, *Flammocladiellaceae*, *Hypocreaceae*, *Myrotheciomycetaceae*, *Nectriaceae*, *Niessliaceae*, *Ophiocordycipitaceae*, *Sarocladiaceae*, *Stachybotryaceae*, and *Tilachlidiaceae* [27]. 

***Nectriaceae*** Tul. & C. Tul., Selecta Fungorum Carpologia: Nectriei-Phacidiei-Pezizei. 3: 3 (1865).

This family, comprising 64 genera, are saprobes, pathogens, and endophytes on human, plant, and insect substrates in aquatic and terrestrial habitats [27]. They also cause important plant diseases and are significant from this point of view [27]. The asexual morph of them is mostly hyphomycetous and, rarely, coelomycetous [27].

***Fusarium*** Link, Magazin der Gesellschaft Naturforschenden Freunde Berlin. 3 (1): 10 (1809).

This genus is characterized by thin- or thick-walled macroconidia with various basal or apical cell shapes, production of trichothecene mycotoxin, and *Giberrella* sexual morph [53]. Species of this genus were identified by morphological characters and phylogenetic data of the *tef1*, *rpb1*, and *rpb2* gene regions [53]. This study reports four species (*Fusarium ipomoeae, F. nirenbergiae, F. pernambucanum*, and *F. sulawesiense*) of the *F. incarnatum-equiseti* and *F. oxysporum* complexes obtained from fern and fern-like hosts using morphological data and multilocus phylogeny. 

**Figure 33:**
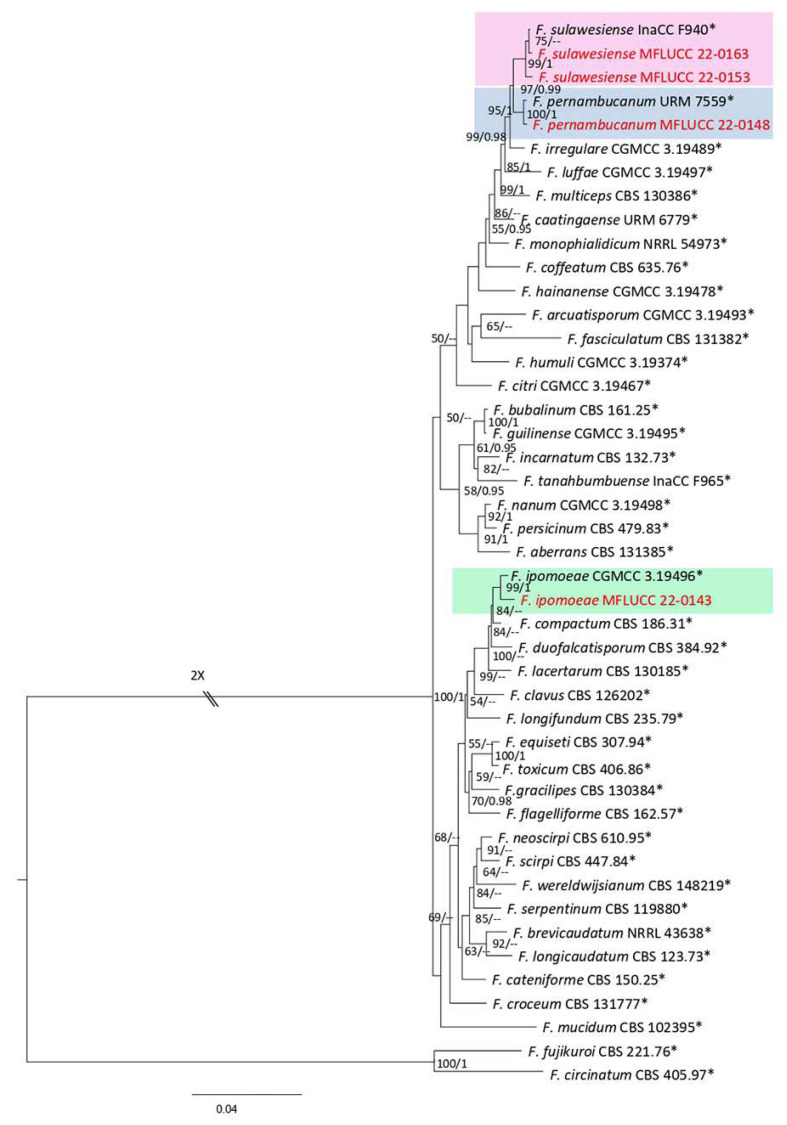
*Fusarium incarnatum* complex. Bayesian phylogenetic tree obtained from the combined *tef1*, *rpb1*, and *rpb2* sequence data. The ultrafast maximum likelihood bootstrap support (%) ≥ 50 and BYPP ≥ 0.95 are shown at the nodes. The ex-type strains are marked with an asterisk. The tree is rooted in *F*. *fujikuroi* (CBS 221.76) and *F*. *circinatum* (CBS 405.97).

***Fusarium ipomoeae*** M.M. Wang, Qian Chen & L. Cai, 2019.

**Figure 34:**
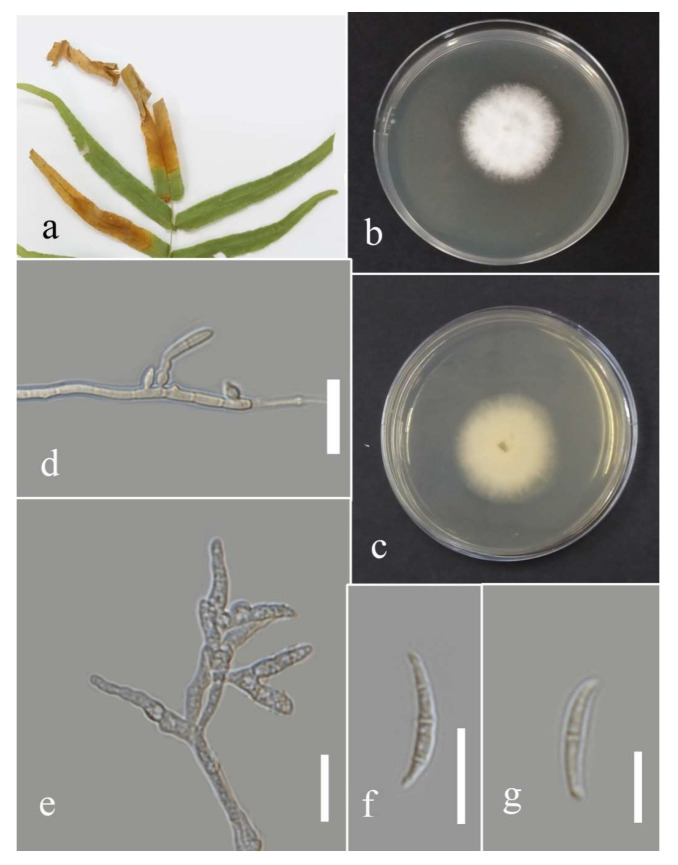
Morphology of *Fusarium ipomoeae* based on the isolate MFLUCC 22-0143: (**a**) leaf blight on *Pteris grandifolia*; (**b**,**c**) upper and reverse views of the colony after seven days of growth on PDA at 28 °C; (**d**) conidiogenous cells; (**e**) phialides; (**f**,**g**) mesoconidia. Scale bars (**d**–**f**) = 20 µm and (**g**) = 10 µm.

Facesoffungi number: FoF 13410; Figure 34.

Associated with *Pteris grandifolia* leaf blight. Arial conidiophores hyaline, branched or unbranched, diverse length, bearing lateral or terminal monophialides and polyphialides, and 15–41 µ × 3–6. Sometimes conidiophores reduced to conidiogenous cells on hyphae. Conidiogenous cells monophialidic, hyaline, and 7–9 × 4–5 µm. Chlamydospore formed on SNA, globes, hyaline, solitary, intercalary, and 10–13 µm diameter. Mesoconidia diverse shape, fusiform or clavate, straight to curved, hyaline, 1–7 cells, aseptate or 1–6 septate, one septate conidia, and 11–24 × 2–5 µm (mean = 16.9 × 3.5, n = 30). Three septate conidia 17–32 × 3–5 µm (mean = 24.0 × 4.0, n = 30). Microconidia not observed. Sporodochium on PDA and SNA not observed. 

Culture characteristics: Colonies reached 38–39 mm after seven days of growth on PDA at 28 °C; fluffy, dull surface, entire edge, fluffy margin, medium density, and without pigmentation in the medium. Upper view was white and the reverse primrose. 

Material examined: Thailand, Chiang Rai Province, Muang District, Thasud, leaf spot on *Pteris grandifolia*, 3 December 2021, Elaheh Seifollahi, dried culture (MFLU 22-0245) and living culture (MFLUCC 22-0143). 

Notes: Strain MFLUCC 22-0143 was isolated from a leaf blight of *Pteris grandifolia*. The isolate obtained in this study (MFLUCC 22-0143) clustered with *F. ipomoeae* in the same clade by 99% ML bootstrap supports and 1.0 BYPP (Figure 33). The comparison DNA sequences of *F. ipomoeae* strains (ex-type strain GCMCC 3.19496 and MFLUCC 22-0143) showed 0.32% nucleotide differences in *tef1* (two nucleotides) and 1.94% in *rpb1* (nine nucleotides), while no difference was found for the sequences of *rpb2*. This species was introduced from *Solanum lycopersicum, Hibiscus syriacus, Lagenaria siceraria, Oryza sativa, Rhododendron pulchrum, Vinca major*, submerged wood, bamboo, and *Capsicum* sp. [54]. Here, we provide a new host and geographical records for *F. ipomoeae*.

***Fusarium pernambucanum*** A.C.S. Santos, C.S. Lima, P.V. Tiago & N.T. Oliveira, 2019.

**Figure 35:**
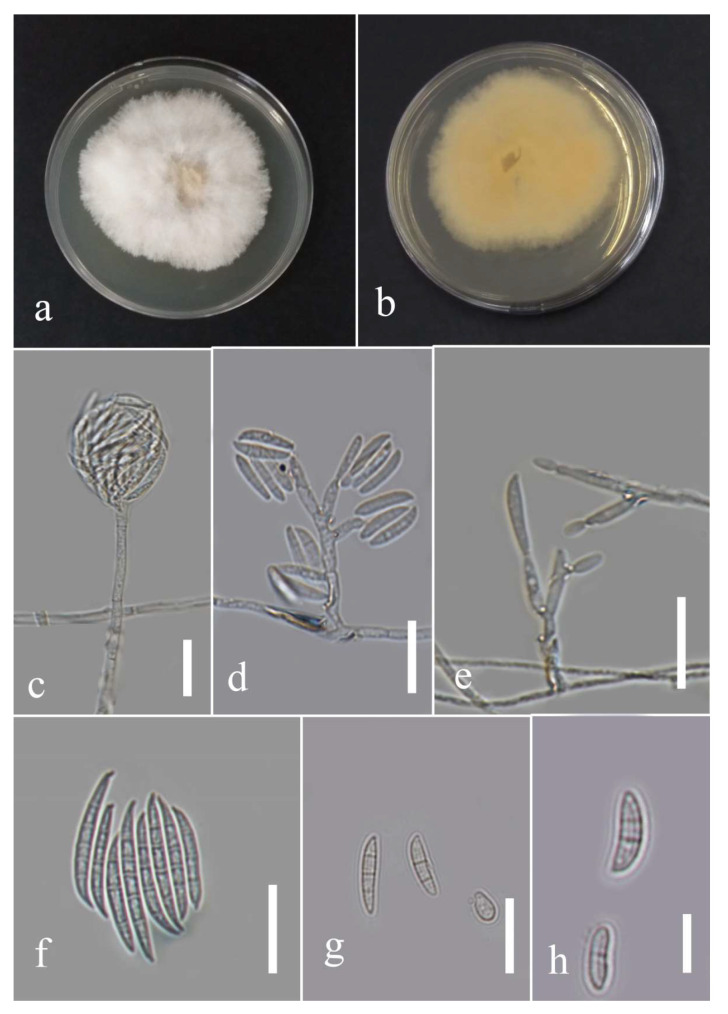
Morphology of *Fusarium pernambucanum* (MFLUCC 22-0148): (**a**,**b**) upper and reverse views of the culture after seven days of growth on PDA at 28 °C; (**c**) false head; (**d**,**e**) phialides; (**f**–**h**) conidia. Scale bars (**c**–**g**) = 20 µm and (**h**) = 10 µm.

Facesoffungi number: FoF 13411; Figure 35.

Associated with a dry stem of *Asparagus setaceous*. Sexual morph: not observed. Aerial conidiophores hyaline, tall or branched sympodially, diverse length, bearing mono and polyphialides, and reduced to conidiogenous cells or not. Monophialides subulate to subcylindrical, hyaline, terminal, or intercalary, and 4–30 µm × 1–4 µ. Polyphialides 7–20 µm × 2–4 µm. Chlamydospores rarely found on PDA, hyaline, intercalary, and solitary. Conidia fusiform, straight to slightly curved, hyaline, 0–6 septate. Four septate conidia 24–33 × 2–5 µm (mean = 29.8 × 3.4, n = 30). One septate conidia 7–16 × 2–5 µm (mean = 11.6 × 3.6, n =30). Microconidia formed in false head on SNA. 

Culture characteristics: Colonies reached 47–68 mm after seven days of growth on PDA at 28 °C; fluffy, circular shape, dull surface, entire edge, fluffy margin, medium density, and without pigmentation in the medium. Upper view is salmon in the center, white in other areas, and the reverse in salmon and primrose areas. 

Material examined: Thailand, Chiang Rai Province, Muang District, Huai Sak, on a dry stem of *Asparagus setaceous* (*Asparagaceae*), 17 December 2021, Elaheh Seifollahi, dried culture (MFLU 22-0246) and living culture (MFLUCC 22-0148).

Notes: Strain MFLUCC 22-0148 was isolated from dry stems of *Asparagus setaceous*. MFLUCC 22-0148 clustered with *F. pernambucanum* in the same clade with 100% ML bootstrap supports and 1.0 BYPP (Figure 33). Comparison of the sequences of the ex-type strain (URM 7559) of this species with strain MFLUCC 22-0148 showed 0.64% nucleotide differences in *tef1* (four nucleotides), while no differences were found in the *rpb1* and *rpb2* genes. *Fusarium pernambucanum* was introduced from *Aleurocanthus woglumi* and *Dactylopius opuntiae* in Brazil [55]. Here, we provide a new host and geographical records for *F. pernambucanum*.

***Fusarium sulawesiense*** Maryani, Sand.-Den., L. Lombard, Kema & Crous, 2019.

**Figure 36:**
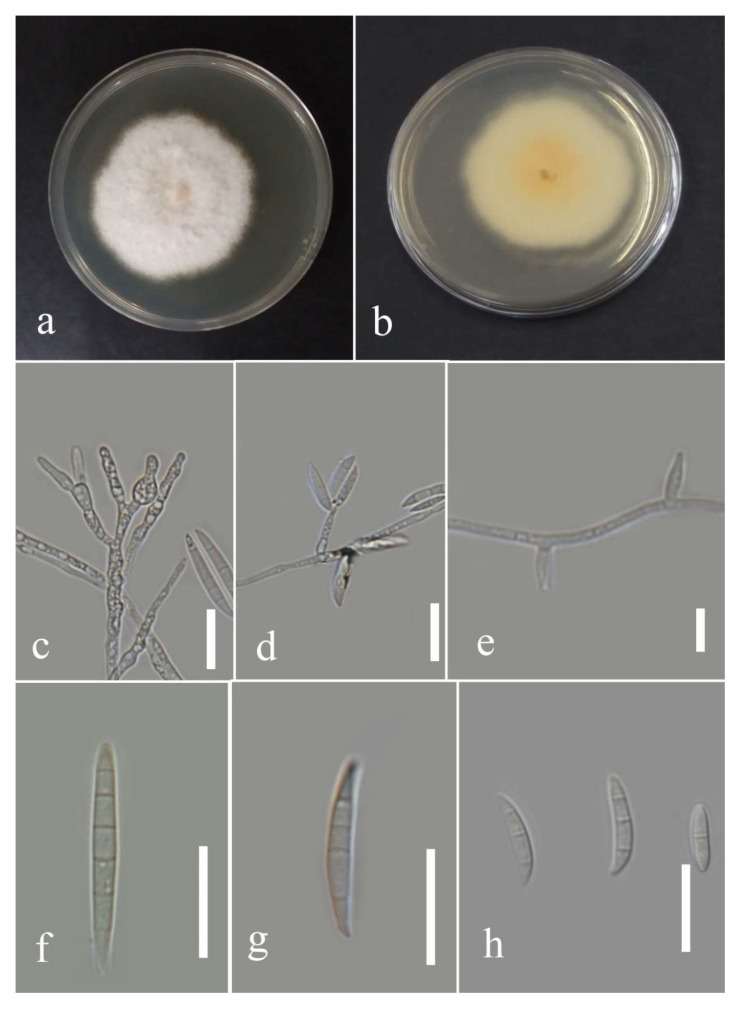
Morphology of *Fusarium sulawesiense* (MFLUCC 22-0163): (**a**,**b**) upper and reverse views of the colony after seven days of growth on PDA at 28 °C; (**c**–**e**) phialides; (**f**–**h**) conidia. Scale bars (**c**,**d**,**f**–**h**) = 20 µm and (**e**) = 10 µm.

Facesoffungi number: FoF 13412; Figure 36.

Saprobe and associated with *Asplenium nidus* leaf spot. Sexual morph: not observed. Arial conidiophores hyaline, branched vertically or irregularly, diverse length, bearing monophialides or polyphialides subulate to subcylindrical, terminal, or lateral. Monophialides 4–35 × 2–4 µm. Polyphialides 12–34 × 3–8 µm. Chlamydospores absent. Arial conidia fusiform, straight to slightly curved (falcate), hyaline, and 0–7 septate. One septate conidia 9–21 × 2–5 µm (mean = 13.6 × 3.6, n = 30). Five septate conidia and 30–43 × 3–5 µm (mean = 35.1 × 4.1, n = 30). 

Culture characteristics: Colonies reached 57–62 mm after seven days of growth on PDA at 28 °C; fluffy, circular shape, dull surface, entire edge, fluffy margin, medium density, and without pigmentation in the medium. Upper view is white to light salmon and the reverse with salmon and straw circles. 

Material examined: Thailand, Chiang Rai Province, Muang District, Thasud, from leaf spot of *Asplenium nidus*, 4 December 2021, Elaheh Seifollahi, dried culture (MFLU 22-0247), living culture MFLUCC 22-0163; *ibid*., on a dead leaf of *Asplenium nidus*, 4 December 2021, Elaheh Seifollahi, dried culture (MFLU 22-0248) and living culture (MFLUCC 22-0153).

Notes: Strain MFLUCC 22-0163 was obtained from a necrotic spot with a brown margin leaf spot on *Asplenium nidus*. The isolates obtained in this study (MFLUCC 22-0163 and MFLUCC 22-0153) clustered with *F. sulawesiense* with 99% ML bootstrap support and 1.0 BYPP (Figure 33). The sequence of the *rpb1* gene is not available for the type strain of this species. A comparison of the ex-type strains of *F. sulawesiense* (InaCC F490) with strains MFLUCC 22-0163 and MFLUCC 22-0153 revealed 0.35% nucleotide differences in *tef1* (two nucleotides between the ex-type and MFLUCC 22-0153), while the *tef1* sequences between the ex-type and MFLUCC 22-0163 and *rpb2* sequences (in all comparisons) were identical. This species was first introduced from *Musa acuminate in* Indonesia [56]. Here, we provide a new host and geographical records for *F. sulawesiense*. 

**Figure 37:**
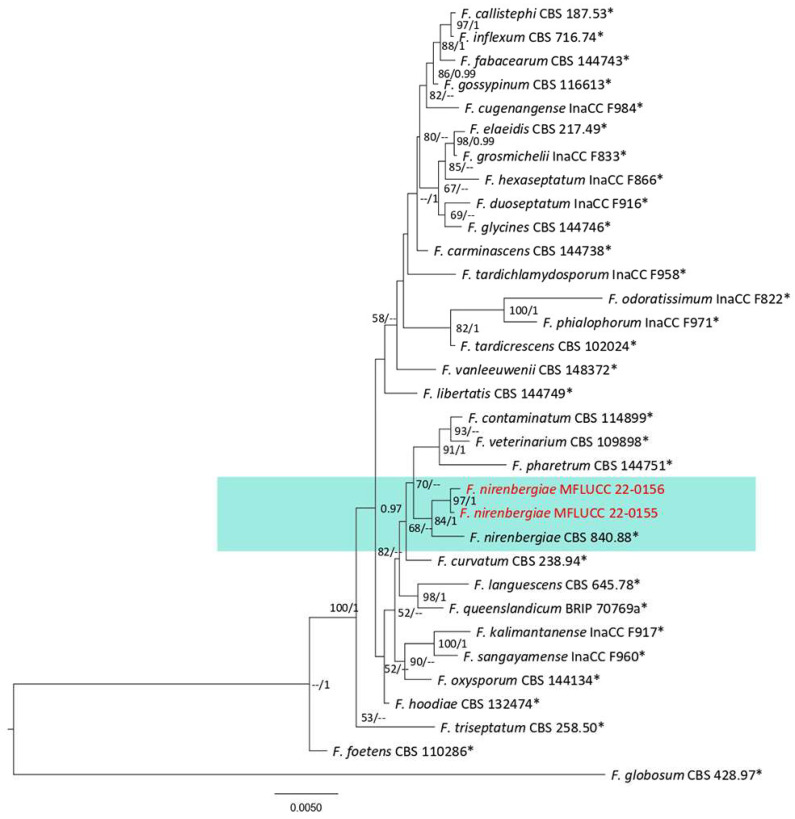
*Fusarium oxysporum* complex. Bayesian phylogenetic tree obtained from the combined of the *tef1*, *rpb1*, and *rpb2* sequence data. The ultrafast maximum likelihood bootstrap support (%) ≥ 50 and BYPP ≥ 0.95 are shown at the nodes. The ex-type strains are marked with an asterisk. The tree was rooted with *F*. *globosum* (CBS 428.97).

***Fusarium nirenbergiae*** L. Lombard & Crous, 2018.

**Figure 38:**
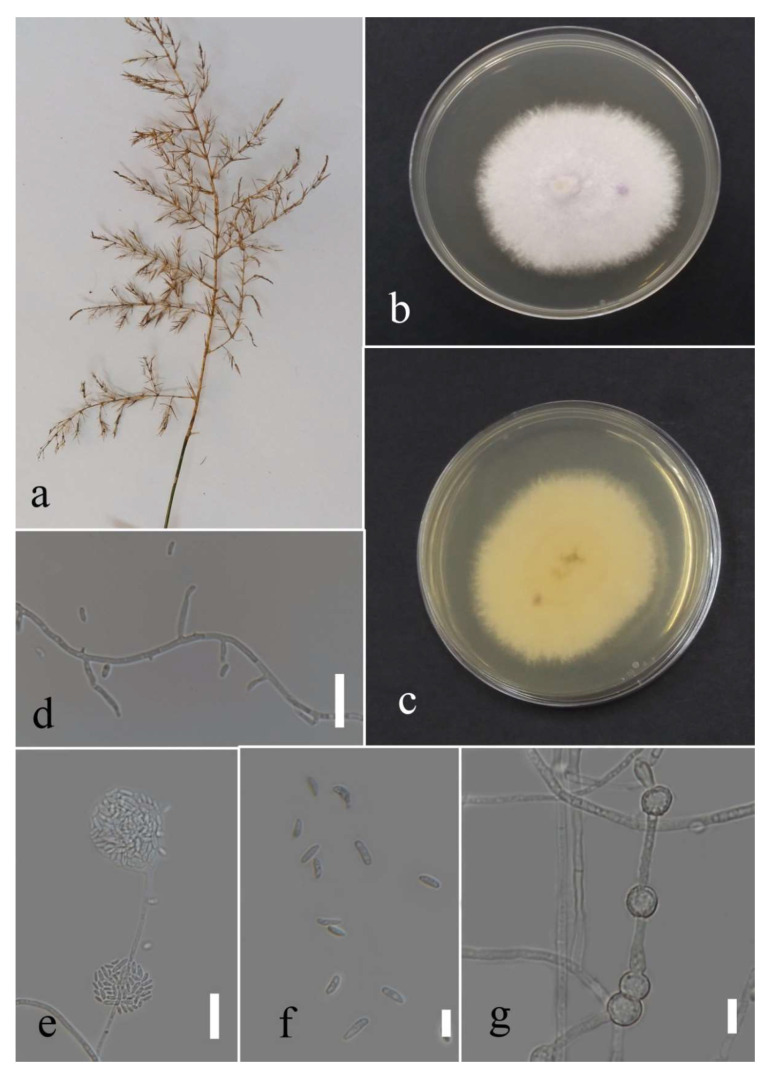
Morphology of *Fusarium nirenbergiae* (MFLUCC 22-0155): (**a**) symptoms on *Asparagus setaceous*; (**b**,**c**) upper and reverse views of the colony after seven days of growth on PDA at 28 °C; (**d**) phialides; (**e**) false head; (**f**) conidia; (**g**) chlamydospores. Scale bars (**d**,**e**) = 20 µm and (**f**,**g**) = 10 µm.

Facesoffungi number: FoF 13413; Figure 38.

Associated with leaf blight of *Nephrolepis cordifolia* and dried branches of *Asparagus setaceous*. Sexual morph: not observed. Aerial conidiophores hyaline, branched sparingly or unbranched, diverse length, bearing monophialides subulate to subcylindrical, hyaline, terminal or intercalary, and 2–19 × 2–3 µm. Chalmydospores ample on PDA, globose to subglobose, hyaline, intercalary or terminally, and 6.0–11.4 µm diameter. Conidia in false head on SNA, ellipsoidal to fusiform, straight or curved, hyaline, aseptate, and 4–11 × 1–4 µm (mean = 6.6 × 2.7, n = 30). 

Culture characteristics: Colonies reached 57–64 mm after seven days of growth on PDA at 28 °C; fluffy, circular, dull surface, entire edge, fluffy margin, medium density, and without pigmentation in the medium. Upper view white to very light rosy vinaceous and the reverse circles of primrose and buff. 

Material examined: Thailand, Chiang Rai Province, Muang District, Thasud, on leaf spot of *Nephrolepis cordifolia*, 3 December 2021, Elaheh Seifollahi, dried culture (MFLU 22-0249) and living culture (MFLUCC 22-0156); ibid., Huai Sak, on diseased dry leaves of *Asparagus setaceous* (*Asparagaceae*), 17 December 2021, Elaheh Seifollahi, dried culture (MFLU 22-0250) and living culture (MFLUCC 22-0155).

Notes: Strain MFLUCC 22-0156 was isolated from leaf blight on *Nephrolepis cordifolia*, while strain MFLUCC 22-0155 was isolated from the dried disease branch of *Asparagus setaceous*. The isolates obtained in this study (MFLUCC 22-0155 and MFLUCC 22-0156) grouped in the same clade with *F. nirenbergiae* by 84% ML bootstrap support and 1.0 BYPP (Figure 37). The sequence of the *rpb1* gene is not available for the ex-type strain of this species (CBS 840.88). Comparison of the sequences of the ex-type strain with strain MFLUCC 22-0155 revealed 0.58% nucleotide differences in *rpb2* (five nucleotides) and 0.16% in *tef1* (one nucleotide). The pairwise comparison results between the ex-type and strain MFLUCC 22-0156 were 0.57% nucleotide differences in *rpb2* (five nucleotides) and 0.33% in *tef1* (two nucleotides). This species was first reported from *Dianthus caryophyllus*, *Passiflora edulis*, *Bouvardia longiflora*, *Solanum lycopersicum*, *Agathosma betulina*, amputated human toe, *Solanum tuberosum*, *Chrysanthemum* sp., tulip roots, human leg ulcer, *Secale cereal*, and *Musa* sp. [57]. Here, we provide a new host and geographical record for *F. nirenbergiae*.

## 3. Discussion

Ferns and fern-like species comprise a significant portion of the world’s flora and are crucial for economic and ecological reasons [2,4,58]. In this study, we collected fungi associated with ferns and fern-like species in Chaing Rai, Thailand, including ornamental (*Asplenium nidus, Asparagus setaceus*, *Nephrolepis cordifolia*, *Nephrolepis* spp., *Phymatosorous* sp., *Pteris ensiformis*, and *P. grandifolia*) and wild (*Cyclosorus* spp.). We introduce one new species and report 30 new hosts of 25 fungal species.

*Colletotrichum fructicola*, *C. polypodialium*, *C. gigasporum*, *C. orchidearum*, and *C. truncatum* species were isolated from the anthracnose symptoms (Figure 21, Figure 22, Figure 24, Figure 26, Figure 27, Figure 28, Figure 29, Figure 30 and Figure 31). Considering the isolation of these species from anthracnose spots on the other hosts [42,50,52,59] and the proof of pathogenicity of some species by other studies [60], our results indicate that ferns can be important hosts for *Colletotrichum* species, contributing to their evolution and fitness. This study obtained multiple associations from anthracnose of three hosts: *Nephrolepis cordifolia* (*C. fructicola*, *C. pandanicola*, and *C. gigasporum*); *Cyclosorus* spp. (*C. pandanicola*, *C. plurivorum* and *C. truncatum*, *C. Orchidearum*, and *C. truncatum*). Therefore, further studies are necessary to identify the dominant species on these hosts to ease controlling the fungus. Moreover, we demonstrated that *C. polypodialium* is able to complete its life cycle as a saprophyte or parasite on *Phymatosorus* sp., characterizing an important characteristic of the biology of this in ferns.

We isolated and identified *C. lunata* from leaf spots on *Pteris grandifolia* (Figure 3 and Figure 4) in Thailand. *Curvularia lunata* was first introduced as a saprobe on decaying leaves of sugarcane [23], while it was also introduced as the leaf spot agent in many hosts, such as corn [61], mulberry [25], and olive [62]. Even though we did not do pathogenicity proofs, based on the mentioned studies, the occurrence of *C. lunata* in *P. grandifolia* characterizes a reservoir for this pathogen and should be noted in the quarantine program and for managing the disease. In addition, the phylogenetic analysis of this article suggested synonymizing some species in the *C. gigasporum* complex and *Curvularia* genus. It is recommended that *C. lunata* and *C. chiangmiensis* be labeled the same species, because they cannot be distinguished by phylogenetic analysis (Figure 3), as also shown by Garcia-Aroca et al. [61]. It also appears that *C. gigsporum* and *C. zhaoqingense* (Figure 26) are essentially one species, which is supported by the nonseparation of the different isolates of these two species in the phylogenetic tree constructed for the *C. gigasporum* complex.

We isolated *D. heveae* and *D. tectonendophytica* associated with leaf spots on *N. cordifolia* (Figure 16 and Figure 19), and *D. delonicis* and *D. chiangriensis* were associated with leaf spots on *Pteris grandifolia* and *Asplenium nidus*, respectively (Figure 16, Figure 17 and Figure 19). Among the *Diaporthe* species that have been reported with different lifestyles, *D. delonicis* and *D. chiangriensis* have been reported as saprobe on dried seed pods of *Delonix regia* and dead twigs of *Bauhinia* sp., respectively [36,38], while *D. tectonendophytica* was introduced as the endophyte [39]. However, all obtained *Diaporthe* species in this study were isolated from leaf spots. Therefore, this indicates a lifestyle switch from endophytes to pathogens or saprophytes [63], representing an increase in their fitness. 

Species of *Fusarium* were isolated from leaf blight on a dry stem of *A. setaceous* (*F. ipomoeae*), dead leaf and leaf spot on *A. nidus* (*F. pernambucanum*), leaf spot on *N. cordifolia (F. nirenbergiae*), and dry leaves of *A. setaceous* (*F. sulawesiense*) (Figure 33, Figure 34, Figure 37 and Figure 38). *Fusarium sulawesiense* and *F. ipomoeae* are pathogenic on sesame [64] and peanut [65], respectively. Additionally, *F. pernambucanum* and *F. nirenbergiae* were obtained from disease symptoms of *Musa* sp., *Passiflora edulis* [66], and potato [57]. This article completed our knowledge of the host range of these species by isolating them from symptoms on new hosts. Furthermore, *F. nirenbergiae* and *F. pernambucanum* were isolated from the dried disease branches of *Asparagus setaceous*, indicating that it is essential to identify the dominant species on this host before treating the related disease on this host.

This article reports *L. thailandica* from a leaf spot on *A. nidus* and a dead leaf of *N. cordifolia* (Figure 1 and Figure 2). *Lasiodiplodia thailandica* was first reported from symptomless twigs of *Mangifera indica* [21]. It was also isolated from canker on branches of *Podocarpus macrophyllus* and *Albizia chinensis* in China [67]. Hence, pathogenicity assays should be carried out to confirm that this species possesses both endophytic and pathogenic phases.

Several species of *Neopestalotiopsis* (*N. guajavicola*, *N. hydeana*, *N. musae, N. pandanicola, N. phangngaensis, N. psidii*, and *N. saprophytica*) were also isolated from leaf spots on ferns and fern-like species (Figure 5, Figure 6, Figure 7, Figure 8, Figure 9, Figure 10, Figure 11 and Figure 12). These species have been reported from several hosts worldwide [30,31]. *Neopestalotiopsis musae* was isolated from a leaf spot on *Musa* sp. [31], while *N. guajavicola* and *N. psidii* were obtained from symptomatic guava tree branches [29]. Additionally, *N. hydeana* was shown to cause fruit rot in *Annona squamosal* and *Garcinia mangostana* and leaf spots on a fern, *Alpinia malaccensis*, and *Garcinia mangostana* [30]. However, *N. saprophytica* was isolated as the saprobe on *Litsea rotundifolia* and *Magnolia* [28] and as the pathogen on *Elaeis guineensis* and *Paphiopedilum micranthum* [68,69]. *Neopestalotiopsis phangngaensis* and *N*. *pandanicola* were introduced as the saprobes [32]. A novel lifestyle or extending host range was thus discovered for all *Neopestalotiopsis* species listed in this study, which were all isolated from leaf spots of various ferns. Three species *N*. *phangngaensis*, *N*. *pandanicola*, and *N*. *saprophytica*, were isolated from *Cyclosorus* spp., a wild fern in Thailand, and should be regarded as the reservoir of these fungi. We also obtained the closely related species, *P*. *dracontomelon* and *P. hydei*, associated with leaf spots on *N. cordifolia* and *Cyclosorus* spp., in the present study (Figure 13, Figure 14 and Figure 15). These species were also reported from leaf spots on *Dracontomelon dao* [33] and *Litsea petiolata* [30]. The new host revealed the new reservoirs for these fungi, which should be considered in quarantine programs and disease management.

Ninety percent (37 out of 41 strains) of the fungi reported in this article were isolated from leaf spots and blights using the tissue culture method. All strains were collected during the dry season from Mueang district in Chiang Rai Province, Thailand. Among the 41 isolates, 26 species were identified, which shows great diversity. Each isolate of *Pestalotiopsis*, *Neopestalotipsis*, and *Diaporthe* isolated from ferns represented single species, showing 100% biodiversity when compared with the other recorded genera. Only approximately 4% of the fungi identified in this paper represent new species. However, Hyde et al. [43] predicted that 96% of the fungi in northern Thailand are new. Thus, limiting the lifestyle (fungi that cause leaf spot), environmental conditions (e.g., dry or wet season), and geographic area also limits identifying the flora of a given area, such as northern Thailand, which has a high fungal diversity [70,71,72,73,74]. In addition, it was documented that the sampling time on fern species affected the diversity, abundance, and composition of fungal flora [75]. Similar to our findings, only one new species was found in the biodiversity survey of associated fungi with ferns in Taiwan, although it has been estimated that between 800 and 8000 new species can be found in the country [7]. Moreover, only one new saprobe was detected in *Alsophila costularis* in Thailand [17]. In addition, the fungal biodiversity of two collections of fern in Mexico revealed 14 records among 21 taxa, and no new species was found [14]. However, 15% of species novelty (two new species among 13 strains) and 92% diversity (12 species among 13 strains) were recorded by Kirschner and Liu [15] in Taiwan, and 76% of the novel taxa (15 new species and on new genera among 21 taxa) was detected on amazon ferns [76] and five new *Pseudocercospora* species were discovered on ferns [77]. Our study showed a snapshot of fungi that can cause spot or blight on ferns and the low or high species novelty on ferns. However, wide collections must be performed in pristine tropical environments to reveal the fungal diversity of ferns and fern-like species.

This study revealed new hosts for 25 fungal species and new lifestyles for some of them (*D. delonicis*, *D. tectonendophytica*, *D. chiangriensis*, *N*. *phangngaensis*, and *N*. *pandanicola*). Identifying the host range of associated fungi with plants is important from several points of view. To manage plant disease using different strategies, including resistant cultivars, quarantine regulations, crop rotations, eradication of reservoirs, disease forecasting, landscape planning, risk modeling, and knowledge of pathogen host range, is necessary [78]. Furthermore, most pathogens have host specificity, meaning they cannot cause damage to nontarget plants. Therefore, they can be employed as a biocontrol agent alongside herbicides or physical techniques to manage exotic plants in agricultural ecosystems [79].

Furthermore, identifying new endophyte hosts is important because some have both pathogenic and no-pathogenic phases, while others only have nonpathogenic phases [63]. As nonpathogenic endophytes are the cause of resistance to heat, salinity, and heavy metals, identifying endophytes whose advantages have been proven in the past leads to the identification of plants that are resistant to salt and heat stress, as well as the identification of plants that are involved in bioremediation [80,81]. In addition, identifying the pathogenic forms of fungi previously reported as saprophytic, provided that their pathogenicity is proven, is necessary to control plant diseases, because it reveals unrecognized gaps in the disease cycle. No correlation between biodiversity and geographic location was found in the hosts where more than one species of the same genus was isolated from leaf spots. Only a small portion of the fungi associated with ferns in northern Thailand was identified in this work. Given the biodiversity of ferns in tropical regions and the paucity of research in this area, it is advised that additional hosts be collected and surveyed to investigate the diversity of fern-associated fungi.

## 4. Materials and Methods

### 4.1. Sampling and Fungal Isolation

Infected leaves and debris of 11 fern and fern-like species were collected from three locations in Chiang Rai Province, Thailand, in December 2021. For the fungal pathogens isolations, they were cut into 4 mm square pieces and sanitized using 1:1 70% alcohol: 1% sodium hypochlorite solution for 1–2 min, followed by a double wash in sterilized distilled water for 1 min [82]. Finally, they were air-dried on sterilized filter paper for 5 min, placed on PDA plates, and incubated in darkness at room temperature. From the debris, cultures were obtained using the imprint or direct culture methods [82]. Pure isolates were obtained using the hyphal tip method. 

### 4.2. Culture Characteristics and Morphological Investigations

Fungal structures were obtained from the sporulating cultures on PDA and SNA, and the morphology of the isolates was investigated. The necessary morphological characters, such as asci, ascospores, conidiomata, conidiophores, phialides, conidiogenous cells, conidia, appressoria, and chlamydospores, were examined. All microscopic characters were observed with a microscope (Nikon Y-TV55, Nikon coopration, Tokyo, Japan), and digital images were captured with a Nikon DS-Ri2 camera. All measurements were made using image framework Version 0.9.7. Images used for photoplates were processed with Adobe Photoshop CS6 v. 13.1.2 (Adobe Systems, USA).

The culture morphology was investigated for all isolates, and the colony character and color were recorded after seven days growth on PDA at 28 ℃. The color was recorded based on Rayner’s color chart [83]. 

### 4.3. DNA Extraction and PCR Amplification

The DNA was extracted using the DNA extraction kit (Omega Bio-Tek). Polymerase chain reaction (PCR) amplifications were performed in a total of 25 μL: 1 μL DNA (50-700 ng/μL), 1 μL of each primer, 12.5 μL Go Taq Green Master Mix 2x (PROMEGA), and 9.5 μL molecular grade water [84] and amplified using the primers and conditions listed in Table 2.

### 4.4. Phylogenetic Analysis

The obtained sequences were blasted against the NCBI no-redundant sequence database, and the sequences of the closely related taxa were downloaded from the GenBank database, comprising different datasets (one per genus). The sequences of each dataset were aligned using ClustalW [99] implemented in Geneious Prime^®^ 2019.1.3 (*tef1* and *tub2* genes for *Neopestalotiopsis*) or the MAFFT v.7 online server [100] (acceessed on 22 August 2022, https://mafft.cbrc.jp/alignment/server/) for the other cases. Sequences where ambiguities were identified were removed from the alignments. The alignments were automatically treated using TrimAl v 1.2 [101] under the *-gapthreshold* of 0.8′ (*tef1* and *tub2* gene regions for *Neopestalotiopsis*) or the *-gappyout* option for the other datasets.

Maximum likelihood (ML) inferences were performed using IQ-TREE v. 1.6.12 [102] in the CIBIV web server ([103] http://iqtree.cibiv.univie.ac.at/, acceessed on 25 August 2022), with the automatic substitution model selection [104], 1000 ultrafast bootstraps [105], and 1000 SH-aLRT branch test replicates [106]. Bayesian inference (BI) was performed in the CIPRES Science Gateway portal (https://www.phylo.org/, acceessed on 28 August 2022) [107]. The best evolutionary model for each genomic region was calculated by jModelTest 2 v.2.1.6 on XSEDE [108], and the BI analyses by MrBayes on XSEDE v.3.2.7a [109] with four independent Markov Chain Monte Carlo (MCMC) chains and four runs. The number of the generations and the sample frequency were optimized based on the number of taxa in each genus, and the stop value was set at 0.01. The first 0.25 of the sampled values was discarded as burn-in. The obtained phylogenetic trees were visualized using FigTree v 1.4.0 (http://tree.bio.ed.ac.uk/software/figtree/, acceessed on 28 August 2022). 

Taxonomic novelties were registered in the Index Fungorum (http://www.indexfungorum.org/names/Names.asp, acceessed on 22 August 2022), and all the obtained sequences of this study were submitted to GenBank (Table 3). 

### 4.5. Pairwise Homoplasy Index Test

To compute the recombination level between the closely related species on the phylogenetic tree, the pairwise homoplasy index (PHI) test [110] was performed by Splits Tree4 v. 4.18.2 [110], using concatenated datasets of the five loci of *Colletotrichum* species. The relationship between species was observed by the splits tree graph and activation of the splits decomposition and logDet options.

## Figures and Tables

**Table 1:** Pairwise differences of the DNA sequences data of the ex-type strain with strains obtained in this study.

Isolate Name	*act*	*chs* *-1*	*gapdh*	ITS	*tub2*
MFLUCC 22-0137	3.62% (7 N *, 1 G *)	1.98% (2 N, 3 G)	0%	1.18% (6 N)	0%
MFLUCC 22-0135	1.44% (3 N)	1.61% (4 N)	0%	1.57% (7 N, 1 G)	0%
MFLUCC 22-0138	1.75% (2 N, 2 G)	1.20% (3 N)	0.48% (1 N)	1.13% (6 N)	0%
MFLUCC 22-0159	1.75% (4 N)	0.40% (1 N)	0.48% (1 N)	0.19% (1 G)	0%
MFLUCC 22-0164	0.44% (1 G)	0.80% (1 N, 1 G)	0%	0.40% (1 N, 1 G)	0.65% (3 G)
MFLUCC 22-0151	0%	1.21% (3 N)	0%	0.40% (1 N, 1 G)	0%

* N = nucleotide; G = gap.

**Table 2:** PCR conditions and used primers in this study.

Locus (Primers)	PCR Condition	Genus	Reference
*act* (ACT-512F/ACT-783R)	95 °C: 5 min, (95 °C: 30 s, 55 °C: 50 s, 72 °C: 1 min) ×40 cycles, 72 °C: 10 min	*Colletotrichum*	[85]
*cal* (CL1/CL2A)	95 °C: 5 min, (95 °C: 30 s, 50.8 °C: 50 s, 72 °C: 1 min) ×40 cycles, 72 °C: 10 min	*Diaporthe*	[86]
*chs-1* (CHS79F/CHS345 R)	94 °C: 5 min, (94 °C: 50 s, 58 °C: 30 s, 72 °C: 1:30 min) ×35 cycles, 72 °C: 10 min	*Colletotrichum*	[85]
*gapdh* (GDF/GDR)	95 °C: 5 min, (95 °C: 50 s, 58 °C: 50 s, 72 °C: 1 min) ×40 cycles, 72 °C: 10 min	*Colletotrichum*	[87]
*gapdh* (gpd1/gpd2)	94 °C: 5 min, (94 °C: 30 s, 54 °C: 50 s, 72 °C: 1 min) ×35 cycles, 72 °C: 10 min	*Curvularia*	[88]
ITS (ITS4/ITS5)	95 °C: 5 min, (95 °C: 45 s, 53 °C: 45 s, 72 °C: 2 min) ×40 cycles, 72 °C: 10 min	All genera in this study	[89]
*rpb1* (Fa/R8)	94 °C: 5 min, (94 °C: 30 s, 53 °C: 50 s, 72 °C: 1 min) ×35 cycles, 72 °C: 10 min	*Fusarium*	[90,91]
*rpb2* (5f2/7cr)	94 °C: 3 min, (94 °C: 30 s, 58 °C: 1 min, 72 °C: 1:20 min) ×40 cycles, 72 °C: 10 min	*Fusarium*	[92,93]
*tef1* (EF-983/EF2218R)	94 °C: 3 min, (94 °C: 30 s, 58 °C: 1:30 min, 72 °C: 1:20 min) ×40 cycles, 72 °C: 10 min	*Curvularia*	[94]
*tef1* (EF-728F/EF2)	94 °C: 3 min, (94 °C: 30 s, 58 °C: 1:30 min, 72 °C: 1:20 min) ×40 cycles, 72 °C: 10 min	*Diaporthe*, *Lasiodiplodia*, *Neopestalotiopsis*, *Pestalotiopsis*	[85,95]
*tef1* (EF1/EF2)	94 °C: 3 min, (94 °C: 30 s, 58 °C: 1:30 min, 72 °C: 1:20 min) ×40 cycles, 72 °C: 10 min	*Fusarium*	[95]
*tub2* (Bt2a/Bt2b)	94 °C: 3 min, (94 °C: 30 s, 58 °C: 1:30 min, 72 °C: 1:20 min) ×40 cycles, 72 °C: 10 min	*Nepestalotiopsis, Pestalotiopsis*	[96,97]
*tub2* (Btub2Fd/Btub4Rd)	94 °C: 3 min, (94 °C: 30 s, 58 °C: 1:30 min, 72 °C: 1:20 min) ×40 cycles, 72 °C: 10 min	*Colletotrichum, Diaporthe, Lasiodiplodia*,	[98]

**Table 3:** GenBank accession numbers of taxa isolated in the present study.

**Species**	**ID**	**Fungarium Code**	**Host**	**ITS**	***tef1***	***tub2***	***act***	***cal***	***chs*** ***-1***	***gapdh***	***rpb1***	***rpb2***
*Colletotrichum fructiola*	MFLUCC ^a^ 22-0147	MFLU ^b^ 22-0223	*Nephrolepis cordifolia*	OP802378		OP801751	OP801696		OP801713	OP801731		
*C. gigasporum*	MFLUCC 22-0158	MFLU 22-0239	*N*. *cordifolia*	OP802376		OP801756	OP801701		OP801719	OP801737		
*C*. *orchidearum*	MFLUCC 22-0152	MFLU 22-0240	*Cyclosorus* sp.	OP802367		OP801743	OP801698		OP801715	OP801733		
*C*. *pandanicola*	MFLUCC 22-0137	MFLU 22-0224	*Nephrolepis cordifolia*	OP802362		OP801740	OP801686		OP801703	OP801721		
	MFLUCC 22-0135	MFLU 22-0225	*Pteris ensiformis*	OP802374		OP801748	OP801693		OP801710	OP801728		
	MFLUCC 22-0138	MFLU 22-0226	*Cyclosporus* sp.	OP802366		OP801742	OP801688		OP801705	OP801723		
	MFLUCC 22-0159	MFLU 22-0227	*N*. *cordifolia*	OP802373		OP801747	OP801692		OP801709	OP801727		
	MFLUCC 22-0164	MFLU 22-0228	*Cyclosporus* sp.	OP802369		OP801744	OP801689		OP801706	OP801724		
	MFLUCC 22-0151	MFLU 22-0229	*Cyclosporus* sp.	OP802371		OP801746	OP801691		OP801708	OP801726		
*C. plurivorum*	MFLUCC 22-0167	MFLU 22-0241	*Cyclosorus* sp.	OP802368		OP801753			OP801716	OP801734		
	MFLUCC 22-0165	MFLU 22-0242	*Cyclosorus* sp.	OP802363		OP801752	OP801697		OP801714	OP801732		
*C. polypodialium*	MFLUCC 22-0178^T^	MFLU 22-0234	*Nephrolepis* sp.	OP802361		OP801739	OP801685		OP801702	OP801720		
		MFLU 22-0235	*Nephrolepis* sp.	OP802370		OP801745	OP801690		OP801707	OP801725		
	MFLUCC 22-0166	MFLU 22-0236	*Phymatosorus* sp.	OP802365		OP801741	OP801687		OP801704	OP801722		
**Species**	**ID**	**Fungarium Code**	**Host**	**ITS**	***tef1***	***tub2***	***act***	***cal***	***chs*** ***-1***	***gapdh***	***rpb1***	***rpb2***
		MFLU 22-0237	*Phymatosorus* sp.	OP802375		OP801749	OP801694		OP801711	OP801729		
		MFLU 22-0238	*Phymatosorus* sp.	OP802377		OP801750	OP801695		OP801712	OP801730		
*C. truncatum*	MFLUCC 22-0177	MFLU 22-0243	*Cyclosorus* sp.	OP802364		OP801754	OP801699		OP801717	OP801735		
	MFLUCC 22-0160	MFLU 22-0244	*Cyclosorus* sp.	OP802372		OP801755	OP801700		OP801718	OP801736		
*Curvularia lunata*	MFLUCC 22-0142	MFLU 22-0209	*Pteris grandifolia*	OP802379	OP830873					OP801738		
*Diaporthe chiangraiensis*	MFLUCC 22-0136	MFLU 22-0221	*Asplenium nidus*	OP802381	OP830875	OP801758		OP830858				
*Diaporthe delonicis*	MFLUCC 22-0133	MFLU 22-0219	*P. grandifolia*	OP802382	OP830876	OP801759		OP830859				
*Diaporthe heveae*	MFLUCC 22-0146	MFLU 22-0220	*N. cordifolia*	OP802380	OP830874	OP801757		OP830857				
*Diaporthe tectonendophytica*	MFLUCC 22-0140	MFLU 22-0222	*N*. *cordifolia*	OP802383	OP830877	OP801760		OP830860				
*F*. *ipomoeae*	MFLUCC 22-0143	MFLU 22-0245	*P* *. grandifolia*		OP830879						OP830862	OP830868
*F*. *nirenbergiae*	MFLUCC 22-0156	MFLU 22-0249	*N. cordifolia*		OP830882						OP830865	OP830871
	MFLUCC 22-0155	MFLU 22-0250	*Asparagus setaceous*		OP830883						OP830866	OP830872
*Fusarium sulawesiense*	MFLUCC 22-0163	MFLU 22-0247	*A. nidus*		OP830878						OP830861	OP830867
	MFLUCC ^a^ 22-0153	MFLU ^b^ 22-0248	*A. nidus*		OP830881						OP830864	OP830870
*F. pernambucanum*	MFLUCC 22-0148	MFLU 22-0246	*A. setaceous*		OP830880						OP830863	OP830869
*Lasiodiplodia thailandica*	MFLUCC 22-0161	MFLU 22-0207	*A*. *nidus*	OP802384		OP801761						
	MFLUCC 22-0154	MFLU 22-0208	*N* *. cordifolia*	OP802385	OP830884	OP801762						
*Neopestalotiopsis guajavicola*	MFLUCC 22-0134	MFLU 22-0210	*Nephrolepis* sp.	OP802393	OP830892	OP801770						
*N. hydeana*	MFLUCC 22-0149	MFLU 22-0211	*Cyclosorus* sp.	OP802386	OP830885	OP801763						
*N. musae*	MFLUCC 22-0162	MFLU 22-0212	*A* *. nidus*	OP802388	OP830887	OP801765						
*N. pandanicola*	MFLUCC 22-0144	MFLU 22-0213	*Cyclosorus* sp.	OP802391	OP830890	OP801768						
*N. phangngaensis*	MFLUCC 22-0157	MFLU 22-0214	*Cyclosorus* sp.	OP802390	OP830889	OP801767						
*N* *. psidii*	MFLUCC 22-0141	MFLU 22-0215	*N. cordifolia*	OP802392	OP830891	OP801769						
**Species**	**ID**	**Fungarium Code**	**Host**	**ITS**	***tef1***	***tub2***	***act***	***cal***	***chs*** ***-1***	***gapdh***	***rpb1***	***rpb2***
*N. saprophytica*	MFLUCC 22-0139	MFLU 22-0216	*Cyclosorus* sp.	OP802389	OP830888	OP801766						
*Neopestalotiopsis* sp.	T22-0234	FT0328	*Cyclosorus* sp.	OP802387	OP830886	OP801764						
*Pestalotiopsis dracontomelon*	MFLUCC 22-0145	MFLU 22-0217	*N. cordifolia*	OP802395	OP830894	OP801772						
*P*. *hydei*	MFLUCC 22-0150	MFLU 22-0218	*Cyclosorus* sp.	OP802394	OP830893	OP801771						

New species and ex-type strain are in bold. T = type strain. ^a^ Mae Fah Luang University culture collection; ^b^ Mae Fah Luang University.

## Data Availability

Not applicable.
